# Salophen-type Organocatalysts for the Cycloaddition of CO_2_ and Epoxides under Solvent, Halide, and Metal-Free Conditions

**DOI:** 10.1021/acsomega.4c00530

**Published:** 2024-04-15

**Authors:** Daniela Fonseca-López, David Ezenarro-Salcedo, Jhon Zapata-Rivera, René S. Rojas, John J. Hurtado

**Affiliations:** † Laboratorio de Química Inorgánica, Catálisis y Bioinorgánica. Departamento de Química, Facultad de Ciencias, Universidad de los Andes, Bogotá 111711, Colombia; ‡ Departamento de Química, Facultad de Ciencias Naturales y Exactas, Universidad del Valle, Cali 760042, Colombia; § Laboratorio de Química Inorgánica, Facultad de Química y de Farmacia, Pontificia Universidad Católica de Chile, Santiago 6094411, Chile

## Abstract

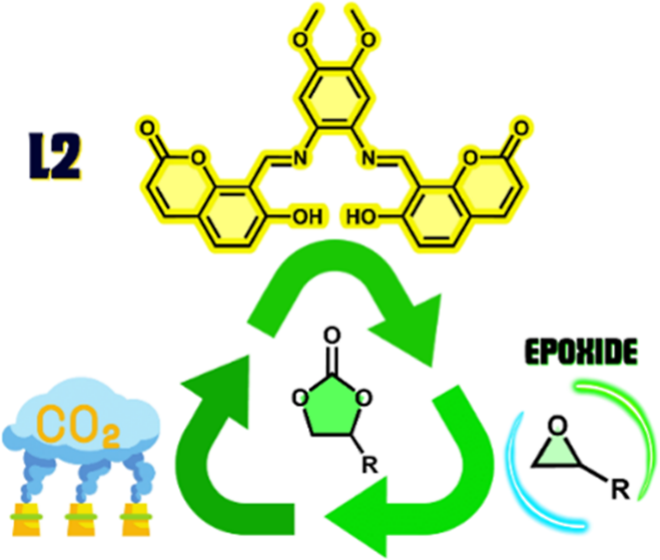

8-Formyl-7-hydroxycoumarin (**A**) and their derived salophen-type organocatalysts **L1**, **L2**, and **L3** were used for the synthesis of cyclic carbonates from carbon dioxide (CO_2_) and epoxides under solvent-, halide-, and metal-free conditions. According to previous optimization tests, **L1** and **L2** had the best catalytic activity presenting 89 and 92% conversion toward the synthesis of 3-chloropropylene carbonate (**2c**) using 8 bar CO_2_, 100 °C at 9 h. Therefore, they were used as organocatalysts to complete the catalytic screening with 11 terminal epoxides (**1a–k**) exhibiting the highest TOF values of 20 and 22 h^–1^ using **1c** and **1b**, respectively. Similarly, they were tested with an internal epoxide, such as cyclohexene oxide (**1l**) exhibiting 72% conversion, becoming the first salophen organocatalyst to obtain *cis*-cyclohexane carbonate (**2l**) in the absence of a cocatalyst. In addition, a reaction mechanism was proposed for the formation of cyclic carbonates based on experimental data and computational techniques; these contributed in establishing a probable role of CO_2_ pressure along the catalysis and the hydrogen bonds that favor the stabilization of the different intermediates of the reaction.

## Introduction

One of the strategies the circular economy uses to mitigate climate change is the capture of greenhouse gases (GHGs) from the atmosphere to transform them into other materials with industrial applications and thus try to counter global warming. Currently, a challenge in the development of methodologies based on sustainable processes for obtaining chemical products is the effective use of renewable resources. Carbon dioxide (CO_2_) is a renewable feedstock since it is a waste product of different industrial activities and is therefore cheap and available.^1,2^ Currently, the chemical industry uses less than 1% of total CO_2_ emissions as a feedstock, mainly for synthesizing urea, salicylic acid, polycarbonates, polyurethanes, and cyclic carbonates.^3,4^ Due to its high stability and low reactivity, CO_2_ requires a well-designed activation and thermodynamic driving force for efficient transformations.^5−7^ Hence, advances have been made for the activation/use of CO_2_, i.e., activation in transition-metal-mediated reactions or by reacting CO_2_ with compounds such as amines, epoxides, and molecular hydrogen.^8−11^ From the green chemistry and sustainability approach, obtaining cyclic carbonates is relevant since they present potential applications, besides being the thermodynamically favored product of this reaction.^12,13^ Cyclic carbonates have a wide use field, such as electrolytic solvents for lithium-ion batteries and environmentally friendly polar aprotic solvents and moreover providing an alternative to the use of phosgene, acting as monomers to produce polycarbonates.^12,14^

**Figure 1:**
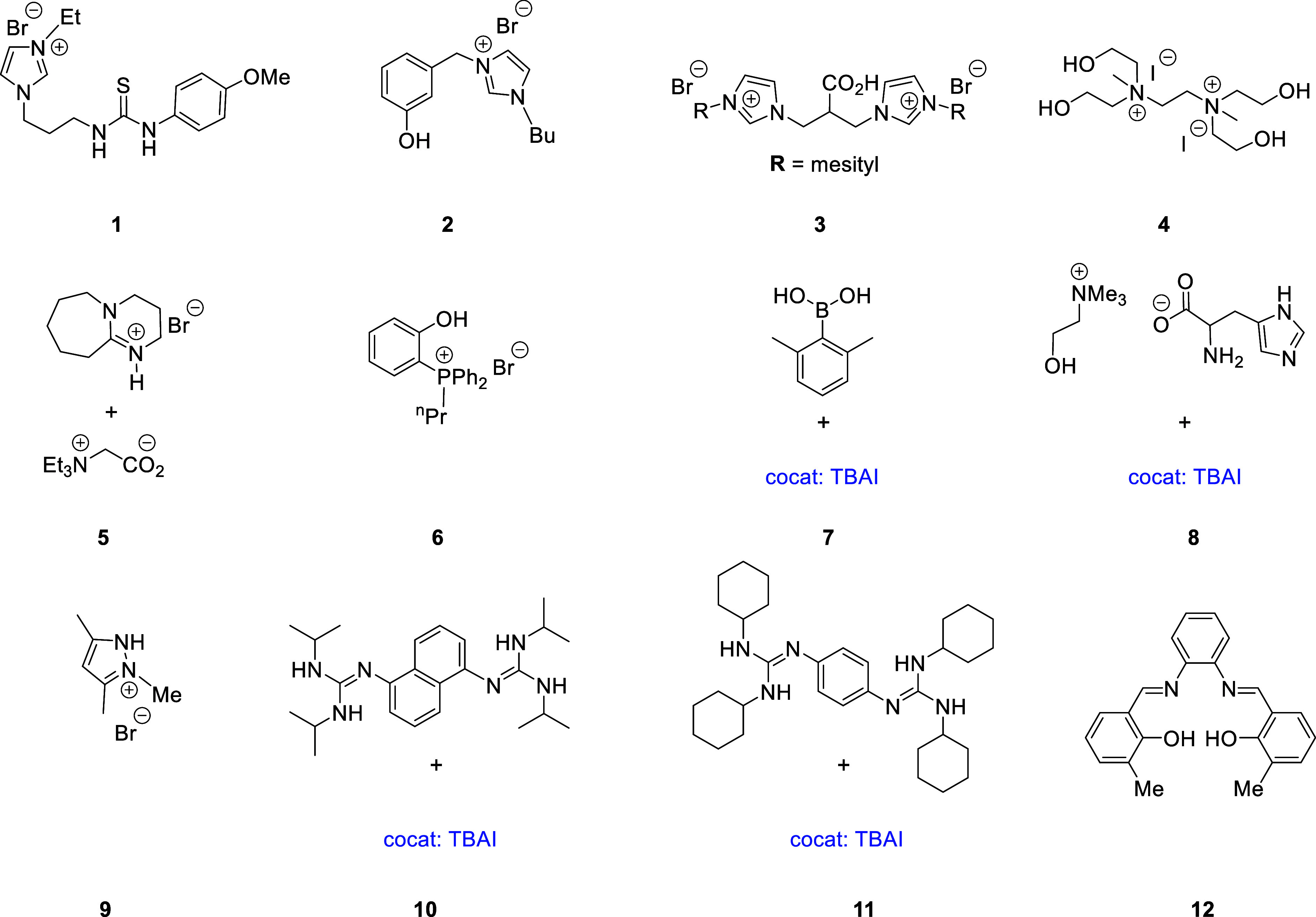
Organocatalysts reported for the synthesis of cyclic carbonates, cocat = cocatalyst.

Additionally, most catalytic systems are based on aluminum(III), chromium(III), cobalt(III), iron(III), and zinc(II), which have shown high efficiency and selectivity, as they generally employ halides (cocatalyst), which causes corrosion in reactor vessels and can be hazardous to the environment as they become waste.^15^ Also, the use of organic solvents and cocatalysts is another source of contamination as they hinder the purification of the carbonate and often prevent the possible recyclability of the catalyst. Therefore, in recent years, the synthesis of organocatalysts as halide- and metal-free catalytic systems has been an increasingly attractive idea for the conversion of CO_2_ into chemical feedstocks, especially in cyclic carbonates. These are considered a low-cost, nontoxic, and sustainable alternative to replace metal-based catalysts. For instance, those derived from ammonium salts,^16^ phosphonium salts,^12,17^ imidazolium salts,^12^ pyrazolium salts,^16^ boronic acids,^15^ guanidines,^14,18^ and Schiff bases compounds,^16^ which in comparison with transition-metal complexes, are air- and moisture-stables (Figure 1).

Accordingly, salen- and salophen-type compounds have recently been studied. In 2019, North et al. investigated an organocatalyst for the synthesis of cyclic carbonates under solvent-free conditions at 120 °C and 10 bar CO_2_ pressure,^16^ indicating that the reaction does not simply occur via the common Lewis or Brønsted acid-catalyzed reaction mechanism. Rather, the two phenolic groups of the organocatalyst salophen have different functions, one of them interacting with the epoxide, favoring its ring opening, and the other forming an intermediate carbonic acid from carbon dioxide. Based on this study, Hong Guo et al. 2021 carried out a DFT study on this mechanism of the whole catalytic cycle, confirming that the kinetically most favored pathway is based on a concerted and synergistic mechanism, where a phenolate acts as a nucleophile and phenol as a hydrogen donor in epoxide ring opening and CO_2_ addition.^19^

In this context, we focused our efforts on the synthesis of a series of organocatalytic coumarin derivatives (**L1**, **L2**, and **L3**). Although salophen-type bisphenol compounds have recently been reported as organocatalysts, coumarin-derived compounds, although they are “salophen-type bisphenol compounds”, are a completely different family of compounds and due to their specific structure, they present very particular steric and electronic characteristics,^20^ which make them suitable candidates for studies as performed in this article. These catalysts were used to carry out catalytic screening with terminal epoxides (**1a–k**) and cyclohexene oxide (**1l**). Furthermore, the reaction mechanism was studied by ^1^H NMR and complemented by computational techniques. Most importantly, the catalysts were effective for CO_2_ fixation under moderate conditions, in the absence of a cocatalyst, and hence being environmentally friendly substitutes for binary catalytic systems. This catalytic system presented equal to higher conversions through different terminal epoxides under milder conditions compared to a similar catalytic system (**12**, Figure 1). A deeper analysis will be shown below.

## Results and Discussion

### Synthesis and Characterization of L3

**Figure 2:**
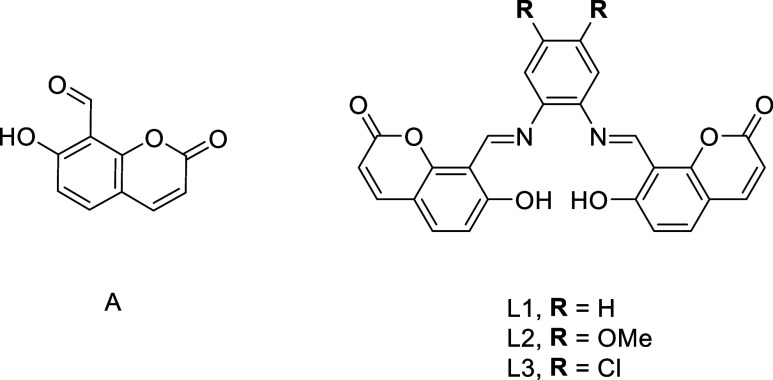
Chemical structure of **A**, **L1**, **L2**, and **L3**.

The compounds **A**, **L1**, and **L2** (Figure 2) were synthesized following previously reported methodologies,^20,21^ and **L3** had not been reported. However, it was synthesized similarly by the double condensation reaction between 4,5-dichloro-*o*-phenylenediamine and 8-formyl-7-hydroxycoumarin. In addition, **L3** was characterized by high-resolution mass spectrometry (HRMS), elemental analysis, UV–vis,^22,23^ Fourier transform infrared,^24−28^ and NMR spectroscopy.

Characterization by elemental analysis determined that the anal. found: C, 59.46; H, 2.68; and N, 5.29% was consistent with the calcd for C_26_H_14_Cl_2_N_2_O_6_: C, 59.90; H, 2.71; and N, 5.37%, thus corroborating the proposed molecular formula. Similarly, the HRMS (ESI+) spectra recorded in acetonitrile (Figure S10) showed a molecular ion peak of 543.01 (*m*/*z*), which is consistent with the **[L3+Na]**^**+**^ adduct. The ^1^H and ^13^CNMR chemical shifts were assigned with the aid of two-dimensional NMR experiments (Figures S11–S15). The ^1^H NMR spectra were recorded in CDCl_3_ (Figure S11) and showed six signals where the two downfield signals (14.2 and 9.4 ppm) are assigned to the phenolic and imine protons, respectively. The downshift of these protons is probably due to an intramolecular hydrogen bond between the hydrogen of this phenolic group and the nitrogen atom of the imine (O–H···N=C).^13^ In the aromatic region (7.8–6.2 ppm), there are four doublets and a singlet (Figure S11). The latter (7.51 ppm) can be assigned to the aromatic protons, and this is confirmed with a COSY experiment (Figure S12). The 7.67 ppm (*J* = 9.6 Hz) and 6.29 ppm (*J* = 9.6 Hz) doublets can be assigned to the protons of the α, β-unsaturated group in the heterocyclic coumarin ring since the coupling constant agrees with that expected for vinylic protons. Finally, the doublets at 7.49 ppm (*J* = 8.7 Hz) and 6.97 ppm (*J* = 8.7 Hz) were assigned to the coumarin aromatic protons (Figure S11). Extra experimental characterization details are given in the Supporting Information.

### Catalysts for the Coupling of CO_2_ and Epoxides

The three salophen compounds **L1**, **L2**, and **L3** (Figure 2) and their aldehyde precursor **A** were initially screened as catalysts for the conversion of epichlorohydrin (**1c**) and styrene oxide (**1k**) into 3-chloropropylene carbonate (**2c**) and styrene carbonate (**2k**), respectively. These epoxides were used due to their difference in reactivity and as a model substrate to compare the activity of the organocatalysts and to achieve the optimum reaction conditions, as shown in Table 1.

**Table 1:**
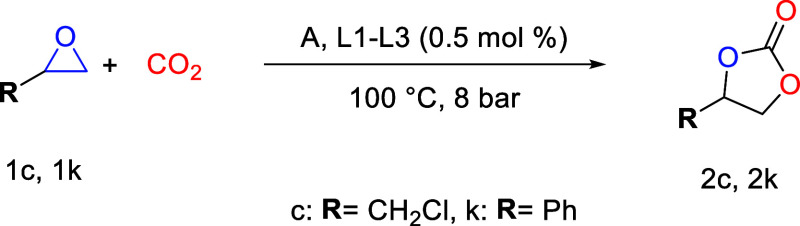
Comparison of the Catalytic Activities of **A** and **L1**-**L3** with **1c** and **1k**^a^

entry	organocatalysts (0.5 mol %)	time (h)	epoxide	conv (%)^b^	TON^c^	TOF^d^
1	**L1**	24	1c	99	198	8.2
2	**L1**	9	1c	89	178	19.8
3	**L1**	24	1k	35	70	2.9
4	**L1**	9	1k	8	16	1.8
5	**L2**	24	1c	99	198	8.2
6	**L2**	9	1c	92	184	20.4
7	**L2**	24	1k	48	96	4
8	**L2**	9	1k	12	24	2.7
9	**L3**	24	1c	99	198	8.2
10	**L3**	9	1c	62	124	13.8
11	**L3**	24	1k	30	60	2.5
12	**L3**	9	1k	6	12	1.3
13	**A**	24	1c	99	198	8.2
14	**A**	9	1c	42	84	9.3
15	**A**	24	1k	23	43	1.9
16	**A**	9	1k	5	10	1.1

a Reaction conditions: epoxide [0.11 mL, 1.40 mmol (**1c**), and 1 mmol (**1k**)], organocatalyst [7.032 and 4.80 × 10^–6^ mmol (0.5 mol %) concerning the initial amount of epoxide **1c** and **1k**, respectively], 100 °C, 8 bar CO_2_ pressure under free-solvent conditions.

b Determined by ^1^H NMR spectroscopy of the crude reaction mixture.

c TON = moles of product/mol of the catalyst.

d TOF = TON/time(h).

The four organocatalysts (**L1, L2**, **L3**, and **A**) were initially studied in the synthesis of **2c** and **2k** at 24 h, 1 mol % catalyst loading, room temperature, and 1 bar CO_2_ pressure, but no conversions were obtained (Table S1). Therefore, the temperature was increased to 100 °C and the pressure increased to 8 bar of CO_2_. Under these conditions, all catalysts showed conversions >99% toward **2c** with a TOF value of 8.2 h^–1^ (Table 1, entries 1, 5, 9, and 13). In the case of **1k**, the highest conversion was 48% with a TOF value of 4 h^–1^ (Table 1, entries 3, 7, 11, and 15). Based on these results, the reaction time was reduced to 9 h and the catalyst loading to 0.5 mol %. With these new conditions, good catalytic activities were observed for **L1**, **L2**, and **L3** (Table 1, entries 2, 6, and 10), being **L2** the one that presented the highest catalytic activity with a conversion of 92% toward obtaining **2c** with the highest TOF value of 20 h^–1^ (Table 1, entry 6). However, **A** presented a moderate activity, with a conversion of 42% to **2c**. In the case of **1k**, when the time is reduced to 9 h, the catalytic activity decreases (Table 1, entries 4, 8, 12, and 16). However, **L2** remained the most active catalyst showing a conversion of 12% toward **2k**. The reason why the results differ significantly between **1c** and **1k** is due to their reactivity, with **1c** being the most active.

**Figure 3:**
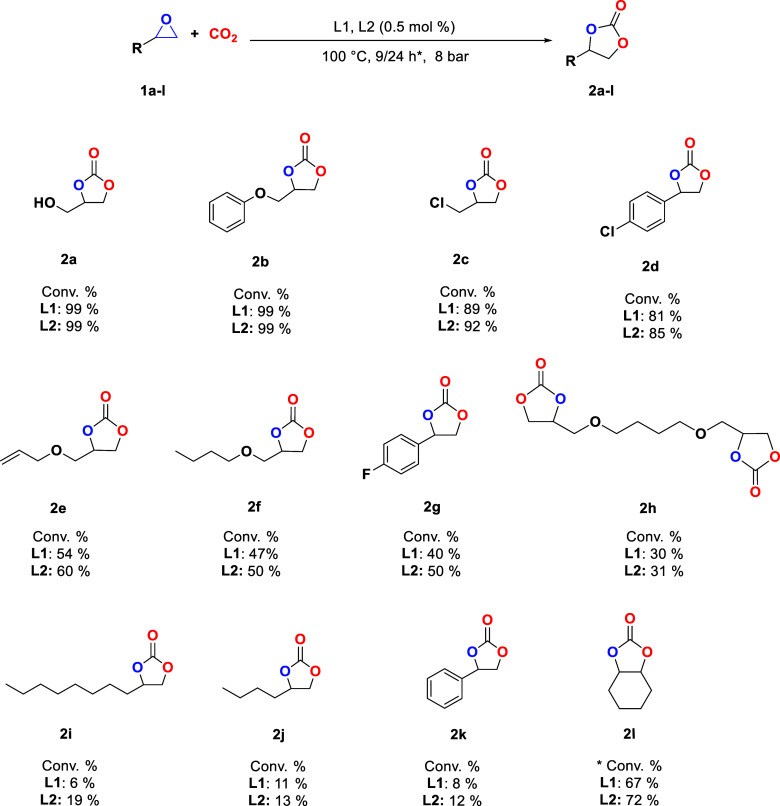
Conversion percentage toward **2a–k** using 8 bar with CO_2_ at 100 °C under solvent-free conditions for 9 h using 0.5 mol % of **L1** and **L2**. *Conversion percentage of **1l** at 24 h reaction time.

With the previous results, since **L1** and **L2** showed the highest conversion (Table 1), both were evaluated in carbonate synthesis from CO_2_ with 11 different terminal epoxides at 9 h, 100 °C, 0.5 mol % catalyst loading, and 8 bar of CO_2_ pressure. In addition, cyclohexene oxide was tested as an internal epoxide but during the 24 h reaction (Figure 3).

Under these conditions, both catalysts showed the highest activity obtaining glycerol carbonate (**2a**), phenyl glycidyl ether carbonate (**2b**), **2c**, and 4-chlorostyrene carbonate (**2d**), with conversions higher than 81%, probably because they have in their backbone electro-withdrawing groups that enhance inductively the electrophilic character of C_α_, facilitating the ring opening of the epoxide. Also, some epoxides like allyl glycidyl ether (**1e**) and butyl glycidyl ether (**1f**) present electro-withdrawing groups, but the solubility of the catalyst decreased due to the backbone chain length. The same solubility problem occurs with 4-fluorostyrene oxide (**1g**) although it has an electro-withdrawing group in its structure; the conversions obtained were 40% and 50%, with **L1** and **L2**, respectively. In the case of 1,4-butanediol diglycidyl ether oxide (**1h**), it showed moderate conversion to **2h**, considering that the catalyst loading was the same (0.5 mol %) as the one used in substrates with a single oxirane group. Therefore, probably, if the load is increased to 1 mol %, it could increase the conversion. Finally, the epoxides 1,2-decilene oxide (**1i**) and 1,2-hexylene oxide (**1j**) presented low conversions due to the presence of an aliphatic backbone (electro-donating groups) that make the ring-opening of the epoxide difficult because of the low electrophilic character of C_α_ (Figure 3).^3^ In the same way that occurred with **1k**, its reactivity could be explained by the steric hindrance of the phenyl ring near C_α_, which made the nucleophile attack difficult for the ring opening.

Because of the catalytic results with terminal epoxides, it was tried with an internal epoxide such as cyclohexene oxide (**1l**) under the same catalytic conditions but with a reaction time of 24 h showing a 72% conversion and 6 h^–1^ TOF value (Table S2) toward **2l**, according to the signals observed in the ^1^H NMR spectrum.^29−31^ The reaction time toward obtaining **2l** was longer because **1l** presents a rigid ring that hinders its activation; therefore, in common with internal epoxides, it is necessary for stronger Lewis acids such as metals. Nonetheless, in this work, the first *cis*-cyclohexane carbonate was obtained from a salophen-type organocatalyst coumarin derivative (Figure 3). From the possible byproducts (polycarbonate and polyethers), all the catalytic reactions in this work were 100% selective toward cyclic carbonates.^29,32,33^

**Scheme 1:**
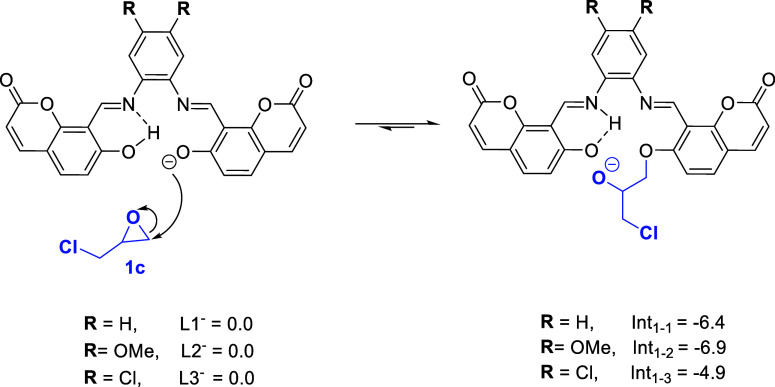
Computational Calculations Comparing Relative Energy (kcal·mol^–1^) for the Formation of the Intermediate **Int**_**1–1**_ with Catalysts **L1** and **Int**_**1–2**_ Using **L2** and **Int**_**1-3**_ with **L3**

Moreover, **L2** showed better catalytic activity than **L1** toward all cyclic carbonates except **2a** and **2b** which were obtained with a complete conversion with both organocatalysts. The better activity of **L2** over **L1** can be explained by the electro-donating behavior (mesomeric effect) of methoxy groups present in the phenyl spacer of the catalyst. In the para-position to the methoxy groups are the imine groups, which are involved in the stabilization of the intermediates of the catalytic reaction through an intramolecular hydrogen bond with the phenolic proton. In addition, the electro-withdrawing effect of the chlorine atoms in **L3** makes it easier for the electrons of nitrogen to be involved in the electronic resonance of the phenyl ring, instead of reinforcing the hydrogen bond with the phenolic proton. We have performed some DFT-based calculations to sustain our assumptions about the influence of the substituents in the aromatic ring spacer of the catalysts (see the Experimental Section for computational details). The Mülliken charges indicate that the methoxy groups enhance the nucleophilicity of the nitrogen of the imine groups (the average Mülliken charges for the two N atoms are −0.269, −0.275, and −0.265 for **L1**, **L2**, and **L3**, respectively) strengthening the N···H···O hydrogen-bond formation, which hence increases the nucleophilicity of phenolic oxygens (the average Mülliken charges for the two O atoms are −0.459, −0.462, and −0.455, respectively), favoring the further nucleophilic attack to epoxides. As depicted in Scheme 1, our computational calculations comparing relative energy for the formation of the intermediate **Int**_**1**–**1**_, the one formed after the nucleophilic attack to epoxide with catalysts **L1**, **L2**, and **L3**, showed that the formation of **Int**_**1–2**_ (using **L2** as catalyst) is the most favorable process (−6.9 kcal·mol^–1^), followed by **Int**_**1–1**_ and **Int**_**1–3**_ (−6.4 and −4.9 kcal·mol^–1^, respectively) in agreement with our prior assumption.

On the other hand, the catalytic activity of **L2** was compared with homogeneous Schiff base-type organocatalysts (**I**) in the literature (Table 2). North et al. used catalyst **I** in the CO_2_ and epoxide coupling for cyclic carbonate synthesis, mainly with terminal epoxides.^16^

**Table 2:**
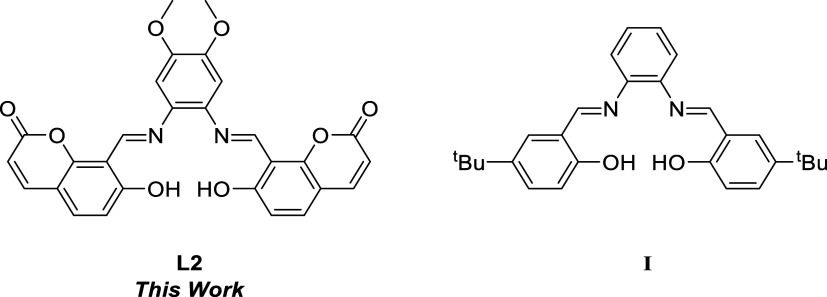
Comparison of the Catalytic Activities of **L2** and **I**^a^

entry	organocat. (mol %)	time (h)	epoxide	conv. (%)^a^	TOF (h^–1^)^a^
1	**I** (1%)^d^	3.5	**1c**	84	24
			**1b**	94	10
2	**L2** (0.5%)^e^	9	**1c**	92	20
			**1b**	99	22
3	**I** (5%)^d^	24	**1i**	71	0.6
			**1d**	96	0.8
4	**L2** (0.5%)^e^	9	**1i**	20	3.2
			**1d**	85	19
5	**I** (10%)^d^	24	**1k**	99	0.4
			**1j**	20	0.1
6	**L2** (0.5%)^e^	9	**1k**	12	2.7
			**1j**	13	4.2
7	**I** (10%)^f^	72	**1l**	-	-
8	**L2** (0.5%)^e^	24	**1l**	72	6

a Organocatalysts: **L2** [8,8′-((1E,1′E)-((4,5-dimethoxy-1,2-phenylene)bis(azaneylylidene))bis(methaneylylidene))bis(7-hydroxy-2H-chromen-2-one)] and **I** [*N*,*N*′-phenylenebis(5-*tert*-butylsalicylideneimine)].

b Determined by ^1^H NMR spectroscopy of the crude reaction mixture.

c TOF = (mol of product)/[(mol of catalyst) time].

d 120 °C and 10 bar CO_2_ (Table S2).

e 100 °C and 8 bar CO_2_.

f 2-Methyl tetrahydrofuran (2-MeTHF) as the solvent.

When initially comparing catalyst **L2** with **I**, **2c** and **2b** were obtained using catalyst **I** with 84 and 94% conversion, respectively (Table 2, entry 1). In this work, **L2** achieved higher conversions for the same cyclic carbonates **2c** (92%) and **2b** (99%) but with half of the catalyst loading (0.5 mol %) (Table 2, entry 2). Additionally, with **1i** and **1d**, catalyst **I** showed conversions of 71 and 96%, respectively, but using longer reaction times, such as 24 h and a catalyst loading of 5 mol %. By contrast, **L2** exhibited lower conversion rates in the synthesis of the same cyclic carbonates, **1i** (20%) and **1d** (85%), but using 0.5 mol % at 9 h. Moreover, **L2** exhibited TOF values higher since the reaction time was shorter (Table 2, entry 1 vs 2) (Table S2). In addition, the reaction of **I** with **1k** and **1j** showed excellent and moderated conversions (Table 2, entry 5). In contrast, **L2** displayed low conversion (Table 2, entry 6). However, it is important to highlight that the catalyst loading of **L2** was 0.5% rather than 10% as required by catalyst **I**. Finally, catalyst **I** did not report any catalytic activity toward internal epoxides such as cyclohexene oxide, even a high-loading catalyst, 72 h of reaction time, and using a solvent. On the contrary, **L2** is the only Schiff base-type organocatalyst that has exhibited CO_2_ and cyclohexene oxide coupling for the cyclohexene carbonate synthesis, showing a conversion of 72% at 24 h and with a low catalyst load (Table 2, entry 7 vs 8).

### Catalyzed Reaction Mechanism toward Cyclic Carbonate

**Figure 4:**
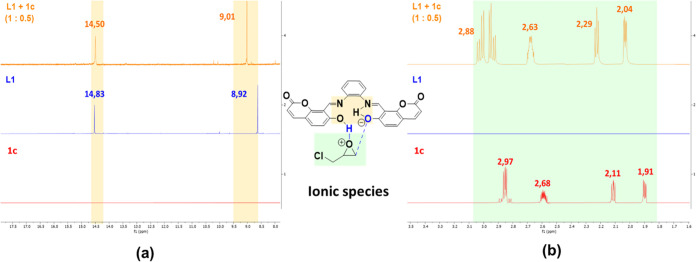
^1^H NMR spectra of **1c** (bottom), **L1** (middle), and **L1**: **1c (1:0.5)** (top), in C_6_D_6_ at 80 °C, in the (a) imine and phenolic region (orange) and in the (b) epoxide **1c** region (green).

Using **L1** as the catalyst, a reaction mechanism for the cycloaddition between CO_2_ and epoxide **1c** has been proposed from computational experiments and NMR measurements; the energy profile of the reaction has been estimated. We carried out some stoichiometric reactions in NMR tubes to detect and characterize the main reaction intermediates. Thus, 1 eq. of **1c** was added to an NMR tube that had previously 1 eq. of **L1** in C_6_D_6_ at 80 °C, and then an ^1^H NMR measure was performed. No significant shifts in the phenolic (14.83–14.81 ppm) and imine (8.92–8.94 ppm) signals were observed (Figure S44b). Therefore, the stoichiometry was modified from 1:1 to 1:0.5 (**L1**:**1c**) in order to detect some changes in both the **L1** and **1c** signals, respectively. First, in the downfield region, the phenolic signal presented an upfield shift from 14.83 to 14.50 ppm (Figure 4). Second, the imine proton signal displayed a slight downfield shift from 8.90 to 9.01 ppm. Finally, at the epoxide region (3.50–2.00 ppm), all the signals showed a downfield shift with respect to the free epoxide spectrum (Figure 4). This fact is presumably due to interactions of phenolic and imine groups with epoxide **1c** rearranging the intramolecular hydrogen bonding to form new intermolecular hydrogen bonds. This epoxide activation is a known phenomenon when the interaction with this kind of organocatalyst occurs.^12^

**Figure 5:**
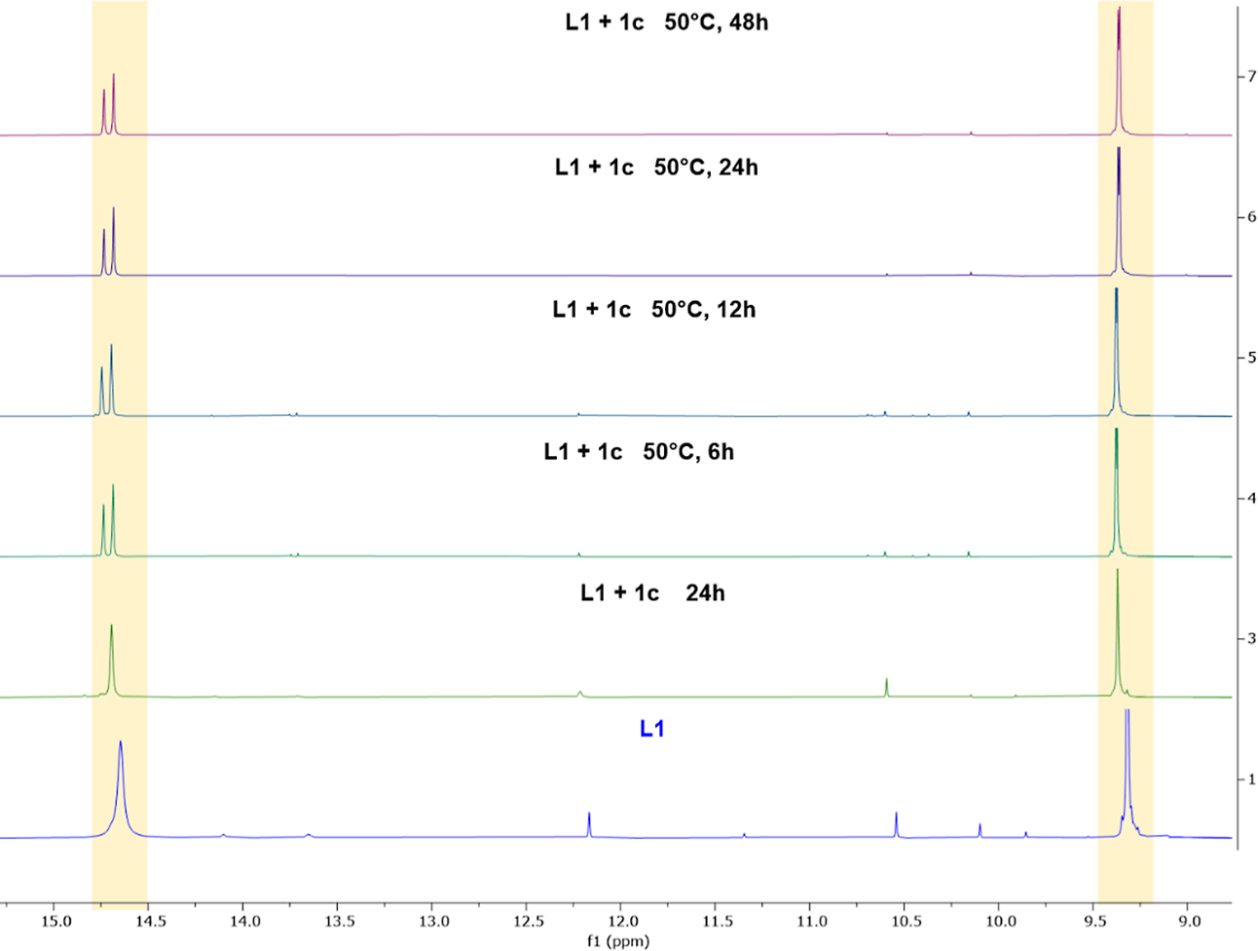
^1^H NMR spectra of **L1**: **1c (1:1)** in CDCl_3_ at 50 °C for 48 h. These changes were not observed in C_6_D_6_, not even with the addition of CO_2_ to the reaction mixture (Figure S47).

In addition, a splitting in the methylene (Cl–CH_2_–CH) protons signal of **1c** (Figure 4) was observed. Overall, all changes in the resonances can be attributed to the synergistic interaction between epoxide **1c** and the catalyst **L1**, i.e., the phenolic oxygen anion interacts with the less-substituted methylene carbon atom of epoxide and the equivalent hydroxyl hydrogen atom interacts with the O atom of epoxide (as in the **1c**^**+**^ and **L1**^**–**^ species depicted in Figure 4). It is worth mentioning that, by exploratory calculations, we have considered two different possibilities for acid ionization of **L1**: (1) proton transfer to **1c** and (2) hydronium formation by a water molecule (Figure S55). Those calculations indicate that the formation of the **1c**^**+**^ + **L1**^**–**^ species is most favored thermodynamically (−6.1 kcal·mol^–1^); in fact, a p*K*_a1_ value of 22.2 was estimated for the first acid ionization of **L1**. The second acid ionization was also calculated (p*K*_a2_ = 26.8) suggesting that the species **L1**^**–2**^ would have low involvement in the reaction. The formation of the **1c**^**+**^ + **L1**^**–**^ ionic pair is also explained by the remarkable deviation in the chemical shifts obtained with the ^1^H NMR experiments when the stoichiometry is 1:0.5 for **L1**:**1c** (Figure 4), namely, at low concentration of epoxide. This ionic pair is expected to be ionized at equivalent (1:1) and high concentrations of **1c** epoxide; no major displacements were observed in their chemical shifts (Figure 5), likely because of the small concentration of **1c**^+^.

**Scheme 2:**
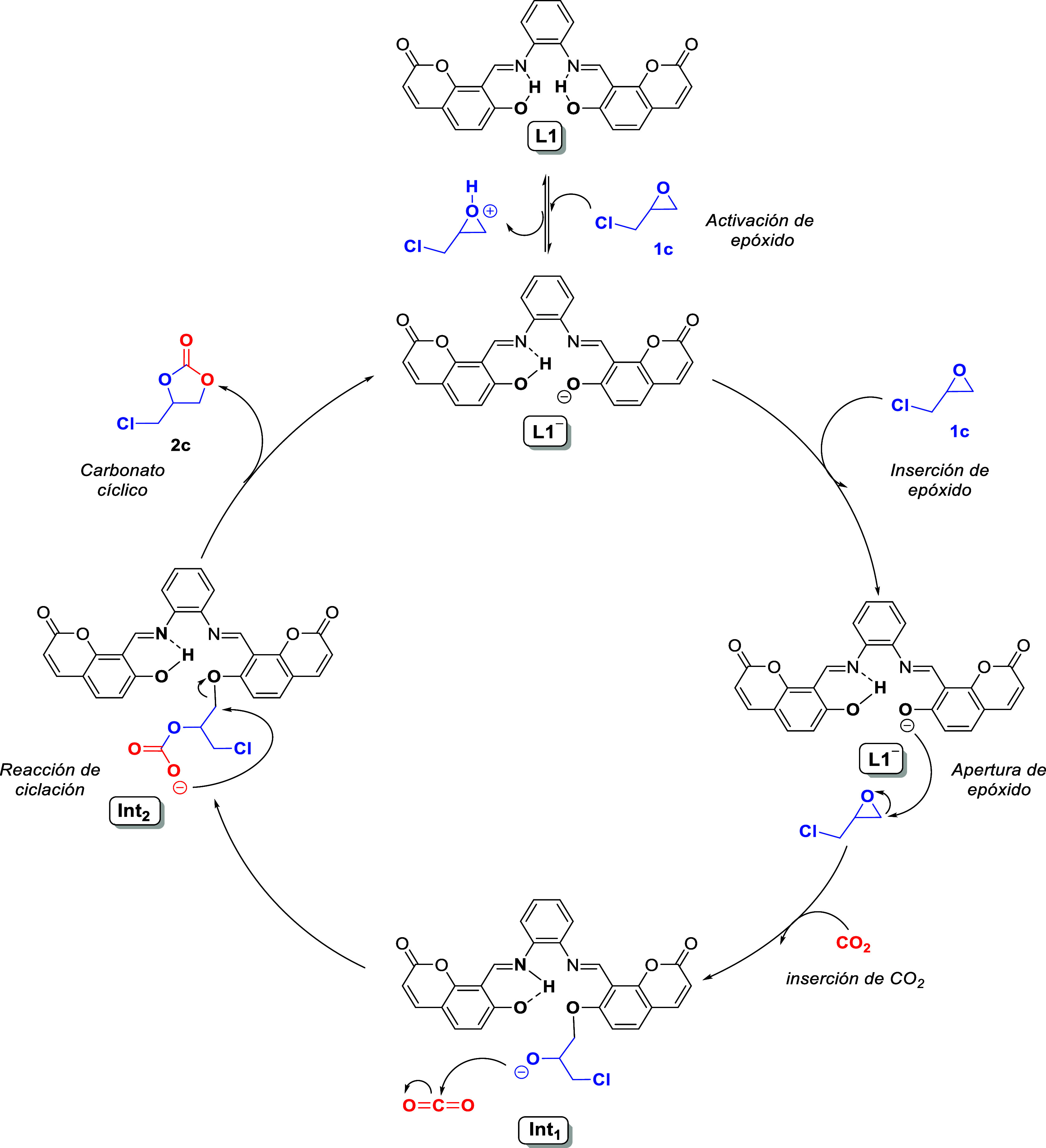
Proposed Reaction Mechanism to Reach **2c** from **1c** and CO_2_ Using **L1** as a Catalyst

In that regard, we have proposed a reaction mechanism starting with the anionic form of the catalyst (**L1**^**–**^) and the nonionized epoxide **1c** in line with that proposed by Guo et al.,^19^ where the abstraction of a hydrogen from a phenolic group in **L1** leads to a more active catalyst and shortened the reaction barriers. This kind of anionic pathways have been also reported for comparable reactions.^34,35^ On the basis of a 1:1 (**L1**: **1c**) stoichiometry for the reaction between **L1** and **1c**, at 50 °C in CDCl_3_, the deviation and growth of the doublet-like signal of phenolic and imine protons were observed (14.70 and 9.37 ppm, respectively; Figure 5), which support the formation of an intermediate with the intramolecular N–H···O hydrogen bond. This dipole–dipole interaction has also been found in the optimized geometry of **Int**_**1**_, in line with formation of the first intermediate of the reaction (Scheme 2).

**Figure 6:**
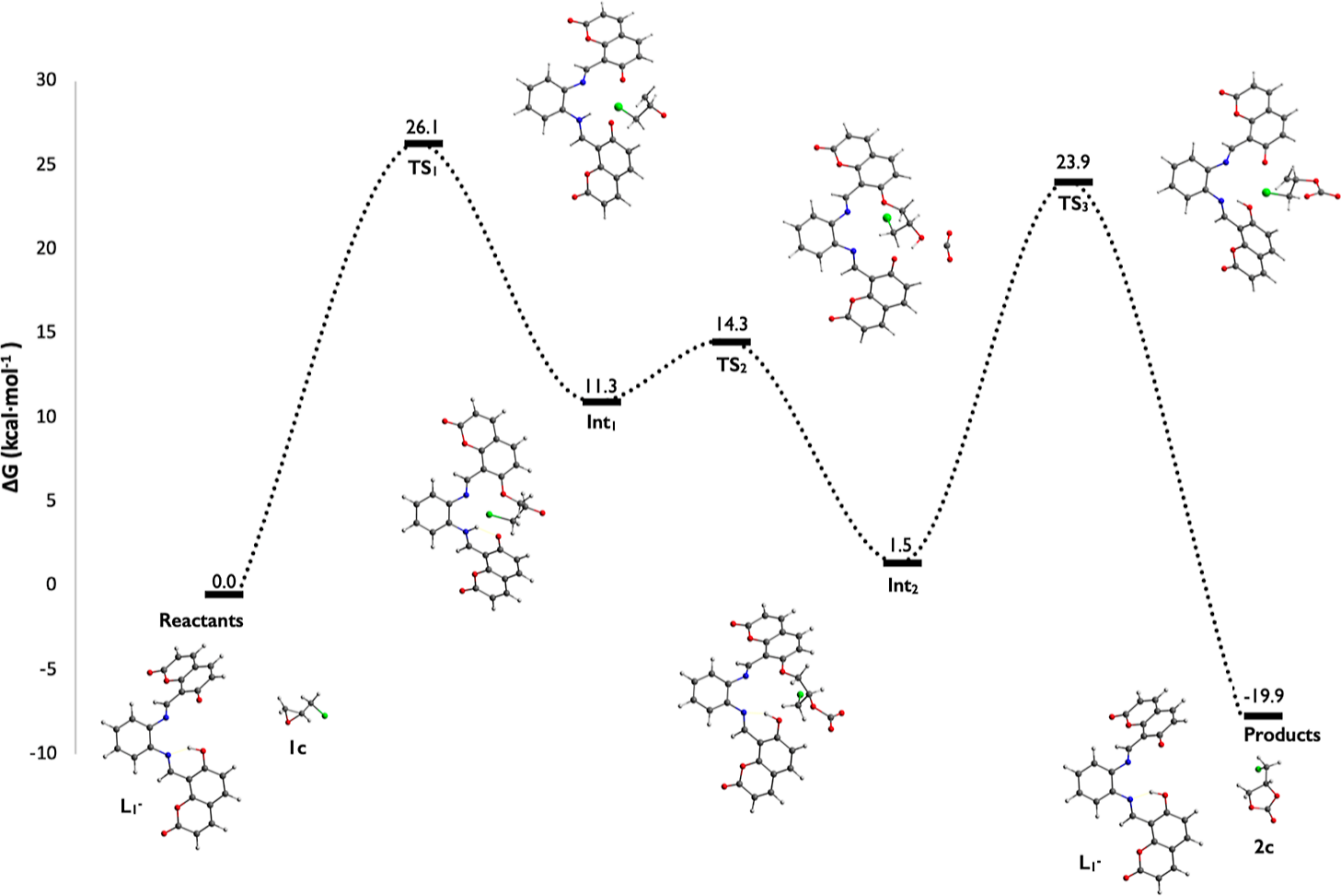
Calculated reaction free energy profile for the formation of **2c** from **1c** and CO_2_ using **L1**^**–**^ as a catalyst.

Our computational estimations of the ^1^H NMR chemical shifts (15.06 and 9.39 ppm, respectively) on **Int**_**1**_ agree with the NMR experimental resonances. As shown in Figure 6, the energy barrier to reach **Int**_**1**_ is Δ*G*_TS1_ = 26.1 kcal·mol^–1^ which is associated with the formation of a first transition state (**TS1**) with a negative frequency of −451.4 cm^–1^. This frequency involves a vibrational normal mode showing the formation of the O–C_β_ bond between **L1**^**–**^ and **1c** (*d*_O–C_ = 2.00 Å) with the concomitant ring opening of **1c**. The large barrier for this step suggests a nonspontaneous process under STP conditions, i.e., a slight heating for the reaction initiation is required. Such a proposal is in line with our experimental procedure; the reaction mixture was heated at about 50 °C. This last statement helped to elucidate the importance of the temperature in the catalysis because it is necessary to start the reaction at a higher temperature (100 °C) as was shown in the initial catalytic essays (Table S1), especially in the activation of the epoxide as it was observed in the mechanism analysis.

The ring opening of **1c** was also confirmed by the appearance of a doublet and triplet at 3.70 and 4.05 ppm, respectively, in the ^1^H NMR spectrum (Figure S52). These signals can be assigned to the alkoxide species when **1c** is attacked by C_β_, corroborating the formation of an **Int**_**1**_-like intermediate. A COSY-NMR experiment was performed to confirm the coupling between these two signals (Figure S53). Afterward, the nucleophilic O atom of **Int**_**1**_ binds to the C atom of a CO_2_ molecule to obtain intermediate **Int**_**2**_ at 1.5 kcal·mol^–1^. This step passes through a lower barrier (Δ*G*_TS2_ = 14.3 kcal·mol^–1^) owing to the formation of **TS2** with a negative frequency of −353.8 cm^–1^; the corresponding vibrational normal mode exhibits the stretching of the O–C bond length (*d*_O–C_ = 1.92 Å). The computed ^1^H NMR chemical shifts of phenolic and imine protons in **Int**_**2**_ showed notorious deviation regarding those of **Int**_**1**_ (14.70 and 9.37 ppm, respectively), in agreement with the changes in the chemical shifts observed in the ^1^H NMR spectra of the reaction mixture after CO_2_ addition (Figure S54). In Figure S54b, the depletion of one of the resonance was observed at about 14.7 ppm after bubbling CO_2_, which is re-established by just heating in a CO_2_ atmosphere. Such a behavior suggests that the catalyst is able to return to the phenol/imine adduct (as **Int**_**1**_) regardless of whether the CO_2_ pressure is not high enough. Also, the signals of the alkoxide intermediate showed a slight shift, likely as a result of the CO_2_ insertion. The latter analysis helped to deduce why at 1 bar of CO_2_ pressure some catalytic essays (Table S1) did not end in product synthesis. Higher pressure is important to proceed with the reaction probably to enhance the CO_2_ solubility in the epoxide and therefore form the carbonate unit.

Following the mechanism, as depicted in Scheme 2 and Figure 6, a ring closing from **Int**_**2**_ takes place to release catalyst **L1**^**–**^ and cyclic carbonate **2c** at −19.9 kcal·mol^–1^. This energy value indicates an exergonic process and a thermodynamically favored reaction. For this stage, we have found a transition state **TS3** (frequency of −497.8 cm^–1^) at 23.9 kcal·mol^–1^, which involves the cleavage of the O–C bond (*d*_O–C_ = 1.99 Å) in the phenolic site with concurrent C–O binding (*d*_C–O_ = 1.97 Å) to a distal O atom in the carbonate moiety (Figure 6). This stage was unable to be followed by ^1^H NMR because it required a higher CO_2_ pressure.

## Conclusions

Organocatalysts **L1**, **L2**, and **L3** were tested toward the synthesis of cyclic carbonate using CO_2_ and 11 terminal epoxides (**1a**–**k**) of which the best conversions were obtained with **1c**, **1i**, **1b**, and **1g**, probably attributed to the presence of electron-withdrawing substituent groups in the epoxide. **L2** displayed the best catalytic activity owing to the high electronic density contribution from the methoxy groups to the imine nitrogen lone pair. As a result, it was found that the substitutions of the phenyl ring (H, OMe, and Cl) by electron-withdrawing or electron-donating groups have a great influence on the catalytic activity. Also, **L2** was tested with an internal epoxide (**1l**) toward the synthesis of **2l** exhibiting a conversion of 72%. A mechanistic study was conducted using NMR and computational techniques. The proposed catalytic cycle starts with the epoxide activation, followed by the formation of the anionic catalyst form (**L1**^**–**^), which is the active catalytic species as it was corroborated by computational studies. A three-step mechanism has been proposed with the formation of two intermediates, **int**_**1**_ and **int**_**2**_, which have also been characterized (only **int**_**1**_ could be corroborated by NMR because **int**_**2**_ requires a higher CO_2_ pressure to result in cyclic carbonate).

## Experimental Section

### General Procedure for Catalyst Screening

The epoxides **1a**–**l** (0.11 mL) and catalyst **A** and **L1**–**L3** (7.032 × 10^–6^ mmol) were placed in a stainless-steel reactor with a magnetic stirrer bar. The reactor was sealed, loaded with 0.5 mol % of organocatalysts under solvent-free conditions, and pressurized to 8 bar with CO_2_ at 100 °C. Finally, the reaction was stopped, and the autoclave was allowed to cool to room temperature. The crude reaction mixture was analyzed by ^1^H NMR spectroscopy to determine the conversion of **1a**–**l** into **2a**–**l**. It is important to highlight that **L1**-**L3** and **A** are air-stable, which allows easier handling when carrying out catalytic tests. Finally, the catalyst did not need the presence of cocatalysts or solvents in any of the catalytic tests.

### Computational Details

All geometry optimization calculations were carried out with the B3LYP functional^36^ and the Ahlrichs def2-TZVP basis set, as implemented in the ORCA 5.0.3 package.^37^ The resolution of identity approximation has been used for the computation of both coulomb and correlation integrals with the def2-TZVP/C auxiliary basis sets.^38−40^ It has also included the long-range interaction by the Grimme dispersion correction (the D3BJ approximation)^41^ and the solvent effects by the conductor-like polarizable continuum model.^42^ Epichlorohydrin has been used as an implicit solvent with values of dielectric constants and refractive indexes of 22.6 and 2.1, respectively. The threshold for the energy convergence in the self-consistent field procedure was set at 10^–8^ au. In addition, at the same level of theory, analytical frequency calculations on the optimized geometries of the stationary points were performed, showing no negative frequencies. However, for each transition-state structure, a large negative frequency was found, which involves a vibrational normal mode associated with the formation/cleavage of specific bonds (see the main manuscript). To predict the uncoupled ^1^H NMR chemical shifts, the NMR shielding tensor was calculated with the gauge including atomic orbital approach (the EPRNMR module in ORCA).^43^ p*K*_a_ values have been estimated according to the procedure developed by Truhlar and coworkers.^44^ A Lorentzian fitting with a line width of 0.05 ppm was used for plotting the peaks in the NMR spectrum. The Avogadro visualization tools have been used for plotting geometries.^45^

## Supporting Information Available

Details on the characterization of the new compound L3 by elemental analysis, FT-IR, UV–vis, ^1^H NMR, ^13^C NMR, two-dimensional NMR spectra, and ^1^H and ^13^C NMR spectra for the cyclic carbonates **2a**-**2l** (PDF)
