# Supplementary material for: Salophen-type Organocatalysts for the Cycloaddition of CO2 and Epoxides under Solvent, Halide, and Metal-Free Conditions
Source: ACS Omega. 2024 Apr 15;9(17):19385–94. doi: 10.1021/acsomega.4c00530 (PMC11064168; doi:10.1021/acsomega.4c00530)
Supplement: Supplementary file 1 — ao4c00530_si_001.pdf [file ao4c00530_si_001.pdf]

Salophen-Type Organocatalysts for the Cycloaddition of CO_2 and Epoxides under Solvent, Halide and Metal-Free Conditions

*Daniela Fonseca-López [a], David Ezenarro-Salcedo [a], Jhon Zapata-Rivera [b], René S. Rojas [c] and John J. Hurtado [a]**

[a] Laboratorio de Química Inorgánica, Catálisis y Bioinorgánica. Departamento de Química, Facultad de Ciencias, Universidad de los Andes, Bogotá 111711, Colombia.

[b] Departamento de Química, Facultad de Ciencias Naturales y Exactas, Universidad del Valle, Cali 760042, Colombia.

[c] Laboratorio de Química Inorgánica, Facultad de Química y de Farmacia, Pontificia Universidad Católica de Chile, Santiago 6094411, Chile.

Contents

1. Experimental 3
   - 1.1 Compounds (L1-L3) and precursor (A) synthesis 3
     - 1.1.1 Synthesis of 8-formyl-7-hydroxicumarin (A) 3
     - 1.1.2 Synthesis of 8,8'-((1E,1'E)-(1,2-phenylenebis(azaneylylidene))bis(methaneylylidene))bis(7-hydroxy-2H-chromen-2-one) (L1) 3
     - 1.1.3 Synthesis of 8,8'-((1E,1'E)-((4,5-dimethoxy-1,2-phenylene)bis(azaneylylidene))bis(methaneylylidene))bis(7-hydroxy-2H-chromen-2-one) (L2) 4
     - 1.1.4 Synthesis of 8,8'-((1E,1'E)-((4,5-dichloro-1,2-phenylene)bis(azaneylylidene))bis(methaneylylidene))bis(7-hydroxy-2H-chromen-2-one) (L3) 4
2. Characterization of A, L1, L2 and L3 5
3. Catalytic results 14
   - 3.1. Optimization of reaction conditions (A, L1-L3) 14
   - 3.2. TON and TOF catalytic results 15
   - 3.3. Characterization of cyclic carbonates by ^{1}H and ^{13}C NMR 16
   - 3.4. ^{1}H and ^{13}C NMR carbonate cyclic spectra 17
4. Catalyzed Reaction Mechanism Toward Cyclic Carbonate 30
5. References 55

1. Experimental

All chemicals were purchased from commercial suppliers (Merck and Aldrich) and used as received unless noted otherwise. 7-Hydroxycoumarin, trifluoroacetic acid, acetic anhydride, sodium bicarbonate, o-phenylenediamine, 4,5-dichloro- O-phenylenediamine, Hexamine, Catechol, methyl iodide, Pd/C 10 wt %, hydrazine monohydrate. Commercial CO_2 was obtained from Gas-Lab and used without further purification. The commercially available epoxide substrates glycidol (**1a**), phenyl glycidyl ether (**1b**), epichlorohydrin (**1c**), 2-(4-chlorophenyl)oxirane (**1d**), Allyl glycidyl ether (**1e**), butyl glycidyl ether (**1f**), 2-(4-fluorophenyl)oxirane (**1g**), 1,4-butanediol diglycidyl ether (**1h**), 1,2-epoxidecane (**1i**), 1,2-epoxyhexane (**1j**), styrene oxide (**1k**) and cyclohexene oxide (**1l**) (99.8%) were purchased from Sigma Aldrich. Elemental analysis of compounds (C, H, and N) was performed on a Thermo Scientific ™ FLASH 2000 CHNS/O analyzer. Melting points were recorded with a capillary Mel-Temp® 1101D Electrothermal apparatus in open capillary tubes (Staffordshire, UK). Fourier transforms infrared (FTIR) spectra were obtained on a Shimadzu IR Tracer-100 spectrometer using a single-reflection ATR accessory. UV–Vis spectra were measured on a Variant Cary 100 spectrophotometer (Agilent Technologies) from 200 to 800 nm in a quartz cuvette with a path length of 1 cm. High-resolution Mass Spectrometry (HRMS) was carried out using a Micromass Quattro Q-TOF LC/MS using electrospray ionization (ESI^+). The NMR spectra were recorded on a Bruker AV-400 spectrometer and referenced to the residual NMR solvent signals.

1.1 Compounds (L1-L3) and precursor (A) synthesis

1.1.1 Synthesis of 8-formyl-7-hydroxicumarin (A)

A mixture of Acetylated 7-hydroxycoumarin (1 g, 6.16 mmol) and trifluoroacetic acid (10 mL) was stirred in an ice bath for 30 min. Then it was added hexamine (HTMA) (1 g, 7.13 mmol) and the reaction was refluxed for 16 h. After cooling to room temperature, the quench was performed with 50 mL 6 M HCl in a vigorous stirring at r.t. The pale-yellow precipitate formed is filtered and washed with 100 ml of cold distilled water. Yield: 1.00 g (60 %). Mp: 187-188 °C. ^{1}H NMR ($CDCl_3$): δ 12.20 (s, 1H), 10.59 (s, 1H), 7.65 (d, *J* = 9.5 Hz, 1H), 7.59 (d, *J* = 8.7 Hz, 1H), 6.88 (d, *J* = 8.7 Hz, 1H), 6.32 (d, *J* = 9.5 Hz, 1H). ^{13}C{1H} NMR: δ 192.8, 165.4,158.6, 156.6, 143.2, 135.8, 114.5, 113.3, 110.7, 108.5. MS (ESI+) *m/z* Calcd. for $[C_{10}H_6O_4+H]^+$: 191.0339; found: 191.0343 $[M+H]^+$.

1.1.2 Synthesis of 8,8'-((1E,1'E)-(1,2-phenylenebis(azaneylylidene))bis(methaneylylidene))bis(7-hydroxy-2H-chromen-2-one) (L1)

This procedure was slightly modified from the literature.[1] A solution of *o*-phenylenediamine (142 mg, 1.31 mmol) in ethanol (15 mL) was added dropwise to a solution of 8-formyl-7-hydroxicumarin (500 mg, 2.62 mmol) in heated ethanol (20 mL). This reaction mixture was stirred at 60°C for 2h. The compound precipitated from the solution as an orange solid. Then, the reaction was cooled to room temperature, the solid was filtered, washed with cold ethanol, and dried under reduced pressure. Yield: 547.4 mg (92%). Mp.: 241-242 °C. FTIR (ATR, cm^{-1}): ʋ 3421w, 3078w, 2164w. 1732vs, 1612vs, 1589vs, 1485s, 1454w, 1404w, 1350w, 1311s, 1230vs, 1180s, 1157w, 1099vs, 999vs, 887s, 833vs, 771vs, 752vs, 640s, 582w, 540s, 478vs, 416w. Anal. Calcd. For ($C_{26}H_{16}N_2O_6$): C, 69.03; H, 3.56; N, 6.19 %. Found: C, 69.15; H, 3.60; N, 6.05 %. ^{1}H NMR ($CDCl_3$): δ 14.67 (s, 2H), 9.39 (s, 2H), 7.64 (d, *J* = 9.5 Hz, 2H), 7.45 (d, *J* = 8.8 Hz, 2H), 7.43 (m, 4H), 6.95 (d, *J* = 8.8 Hz, 2H), 6.26 (d, *J* = 9.5 Hz, 2H). ^{13}C{1H} NMR: δ 166.9, 160.3, 157.5, 155.4, 144.2, 141.3, 132.9, 128.9, 119.9,

115.7, 112.3, 110.3, 107.3. MS (ESI +) *m/z* Calcd. for $[C_{26}H_{16}N_2O_6+H]^+$: 453.1081; found 453.1120 $[M+H]^+$. UV/Vis (EtOH, nm ($mol^{-1}dm^3cm^{-1}$)): λ_{max} (ε)=328 (37153), 464 (1995).

1.1.3 Synthesis of 8,8'-((1E,1'E)-((4,5-dimethoxy-1,2-phenylene)bis(azaneylylidene))bis(methaneylylidene))bis(7-hydroxy-2H-chromen-2-one) (L2)

This procedure was slightly modified from the literature.[2] A solution of 1,2-dimethoxy-4,5-diaminobenzene (222.04 mg, 1.316 mmol) in EtOH (15 mL) under stirring and refluxing, was added dropwise to a solution of 8-formyl-7-hydroxicumarin (500 mg, 2.62 mmol) dissolved in EtOH (15 mL). The reaction mixture was refluxed for 2 h. The precipitate obtained was filtered and washed with cold EtOH (9 x 10 mL) and diethyl ether (1 x 10 mL) to give the product a reddish-orange. Yield: 0.333 g (78 %). Mp: 261-263 °C. FTIR (ATR, cm^{-1}): υ. 3591w, 3460w, 3062vw, 3005vw, 2939vw, 2839vw, 2360vw, 1743vs, 1716vs, 1612vs, 1508vs, 1462s, 1404w, 1354w, 1311w, 1269s, 1238s, 1188w, 1157w, 1122s, 1010s, 918w, 837vs, 775w 709vw, 640vw, 617vw, 547vw, 478w, 424vw, 366vw. Anal. Calcd. For ($C_{28}H_{20}N_2O_8$): C, 65.62; H, 3.93; N, 5.47 %. Found: C, 65.68; H, 4.01; N, 5.43 %. ^{1}H NMR ($CDCl_3$) δ 14.86 (s, 2H), 9.32 (s, 2H), 7.65 (d, *J* = 9.5 Hz, 2H), 7.43 (d, *J* = 8.8 Hz, 2H), 6.94 (d, *J* = 8.8 Hz, 2H), 6.92 (s, 2H), 6.25 (d, *J* = 9.5 Hz, 2H), 4.04 (s, 6H). $^{13}C\{^1H\}$ NMR: δ 166.7, 160.5, 155.2, 150.0, 144.3, 134.3, 132.6, 115.7, 112.1, 110.3, 107.4, 102.5. MS (ESI+) *m/z* Calcd. for $[C_{28}H_{20}N_2O_8+H]^+$: 513.1298; found: 513.1294 $[M+H]^+$. UV/Vis (DMSO, nm ($mol^{-1}dm^3cm^{-1}$)): λ_{max} (ε)=333 (41686), 258 (33884).

1.1.4 Synthesis of 8,8'-((1E,1'E)-((4,5-dichloro-1,2-phenylene)bis(azaneylylidene))bis(methaneylylidene))bis(7-hydroxy-2H-chromen-2-one) (L3)

8-formyl-7-hydroxycoumarin (0.20 g, 1.052 mmol) was dissolved in 25 mL EtOH and added to a solution of 4,5-dichloro-*o*-phenylenediamine (0.06 g, 0.525 mmol) in EtOH (10 mL). After 4 h, the reaction crude was washed with cold EtOH (5x10 mL) and ethyl acetate (2x10 mL) to give the product as a reddish brown solid. Yield: 0.33 g (90 %). M.p.: 261–263 °C. FTIR (ATR, cm^{-1}): υ 3437w, 3082w, 1732vs, 1612vs, 1473s, 1404w, 1346w, 1311w, 1234s, 1107w, 1002s, 898vs, 837vw, 775vw; Anal. Calcd. for $C_{26}H_{14}Cl_2N_2O_6$: C, 59.90; H, 2.71; N, 5.37 %. Found: C, 59.46; H, 2.68; N, 5.29 %. ^{1}H NMR ($CDCl_3$): δ 14.17 (s, 2H), 9.36 (s, 2H), 7.67 (d, *J* = 9.5 Hz, 2H), 7.51 (s, 2H) 7.49 (d, *J* = 8.7 Hz, 2H), 6.97 (d, *J* = 8.7 Hz, 2H), 6.29 (d, *J* = 9.6 Hz, 2H); $^{13}C\{^1H\}$ NMR: 165.8, 160.0, 158.5, 155.2, 143.9, 141.0, 133.3, 132.3, 121.3, 115.2, 112.6, 110.6, 107.1; HRMS (ESI+) m/z Calcd. for $[C_{26}H_{14}Cl_2N_2O_6+Na]^+$: 543.0127. Found: 543.0197 $[M+Na]^+$. UV/Vis (DMSO, nm ($mol^{-1}dm^3cm^{-1}$)): λ_{max} (ε)=348 (66069), 260 (36307).

2. Characterization of A, L1, L2 and L3

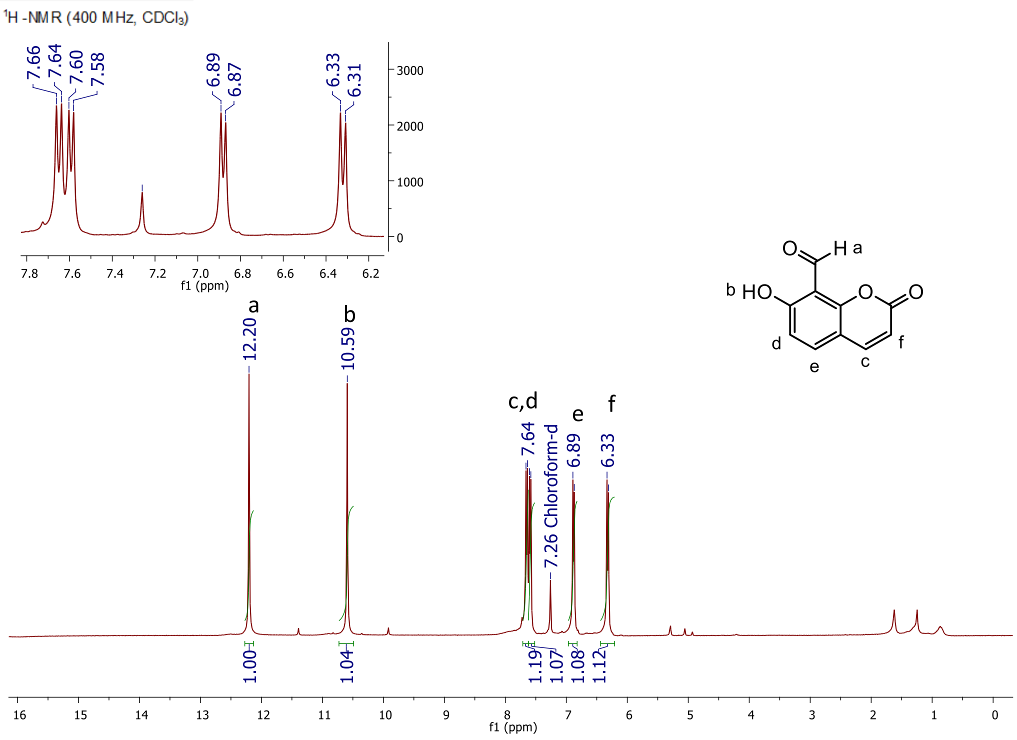

Figure S1.^{1}H RMN spectrum of **A** in $CDCl_3$.

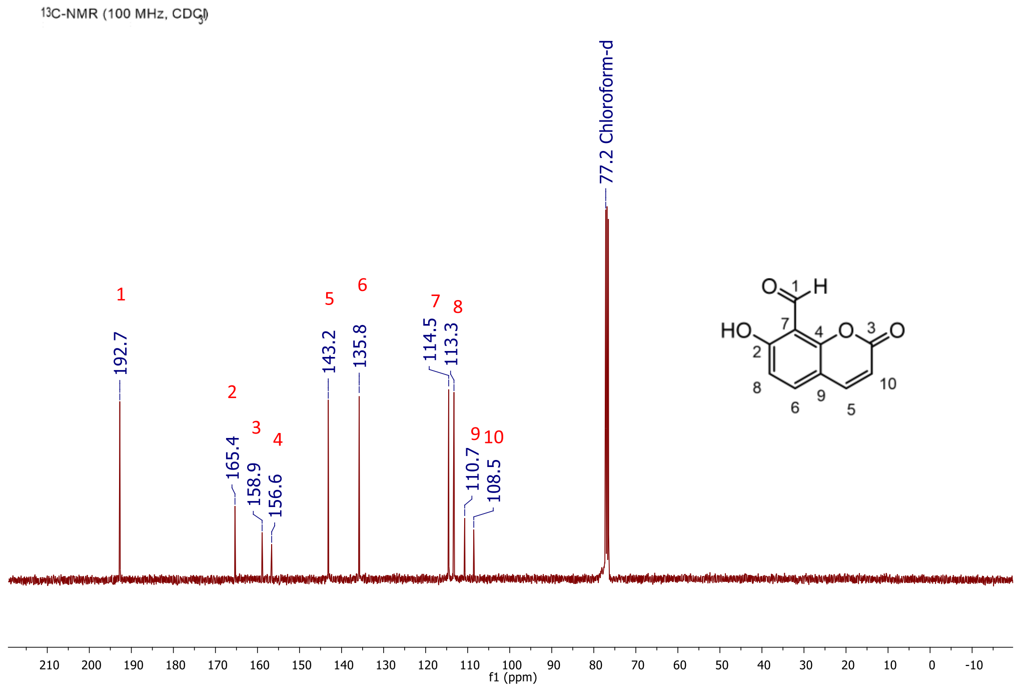

Figure S2.[13] C RMN spectrum of **A** in $CDCl_3$.

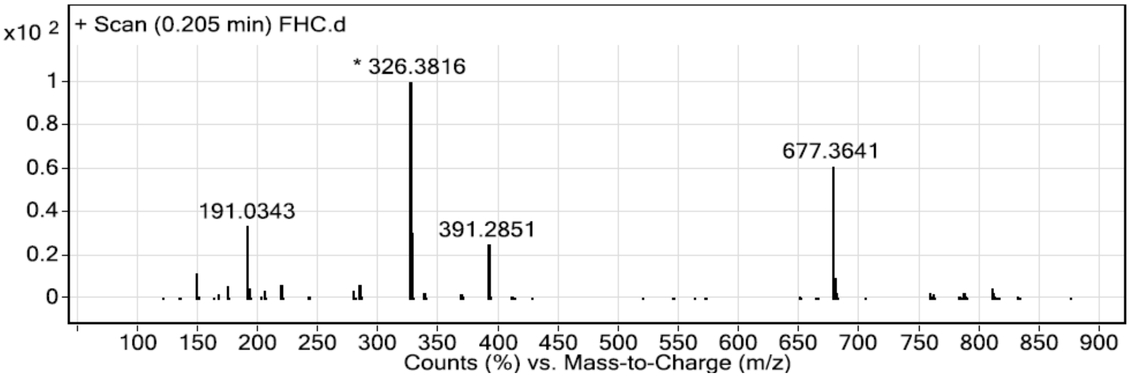

Figure S3. High-resolution mass spectrum (HRMS) of **A** (solvent: acetonitrile).

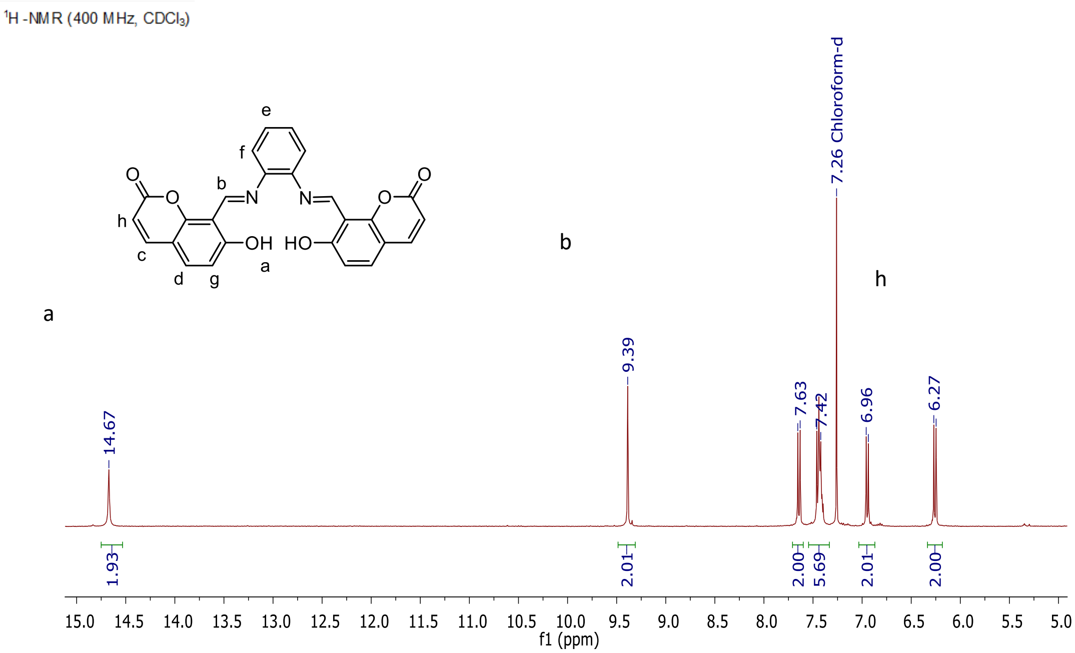

Figure S4. ^{1}H RMN spectrum of **L1** in $CDCl_3$.

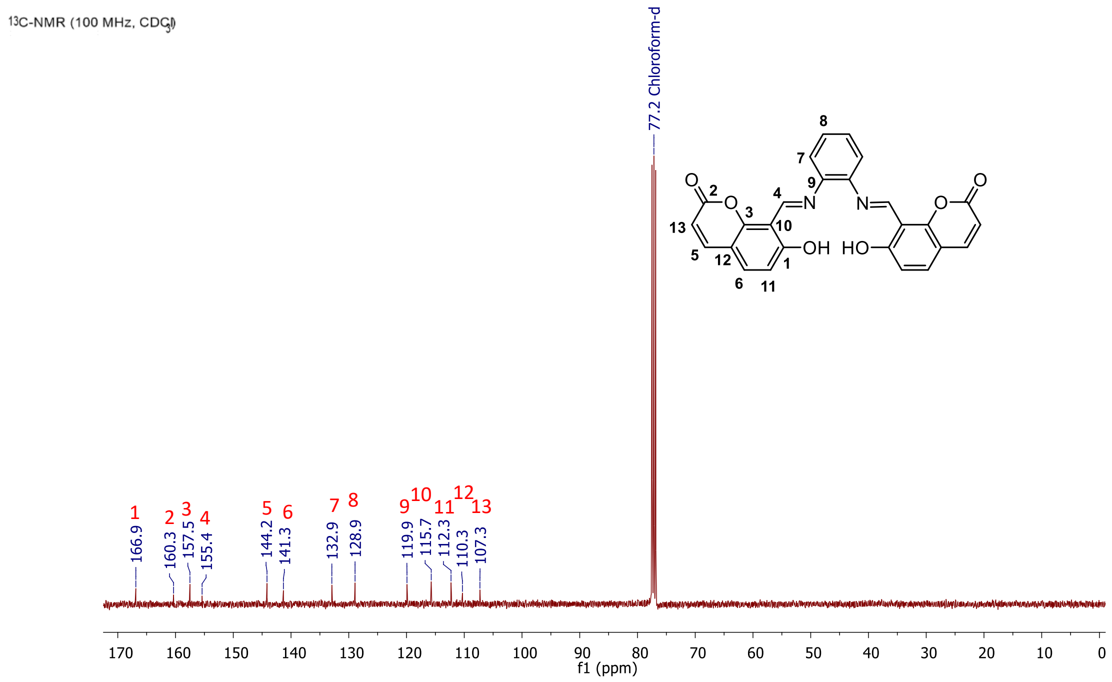

Figure S5. ^{13}C RMN spectrum of **L1** in $CDCl_3$.

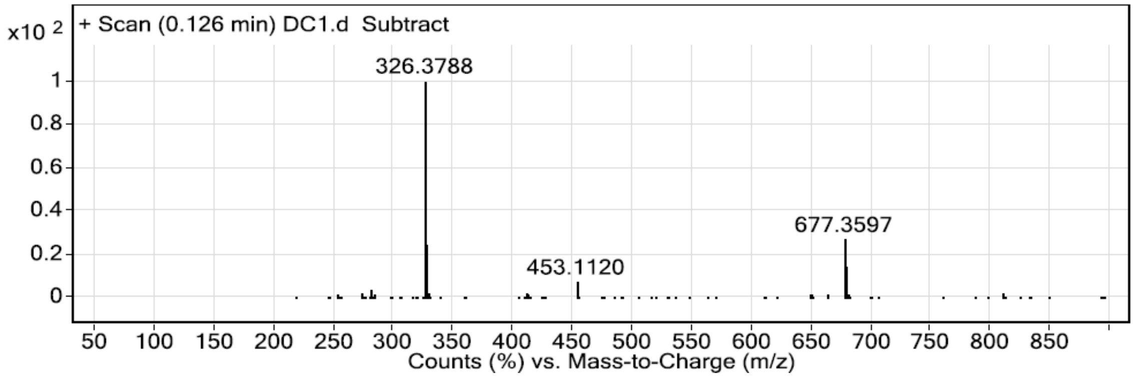

Figure S6. High-resolution mass spectrum (HRMS) of **L1** (solvent: acetonitrile).

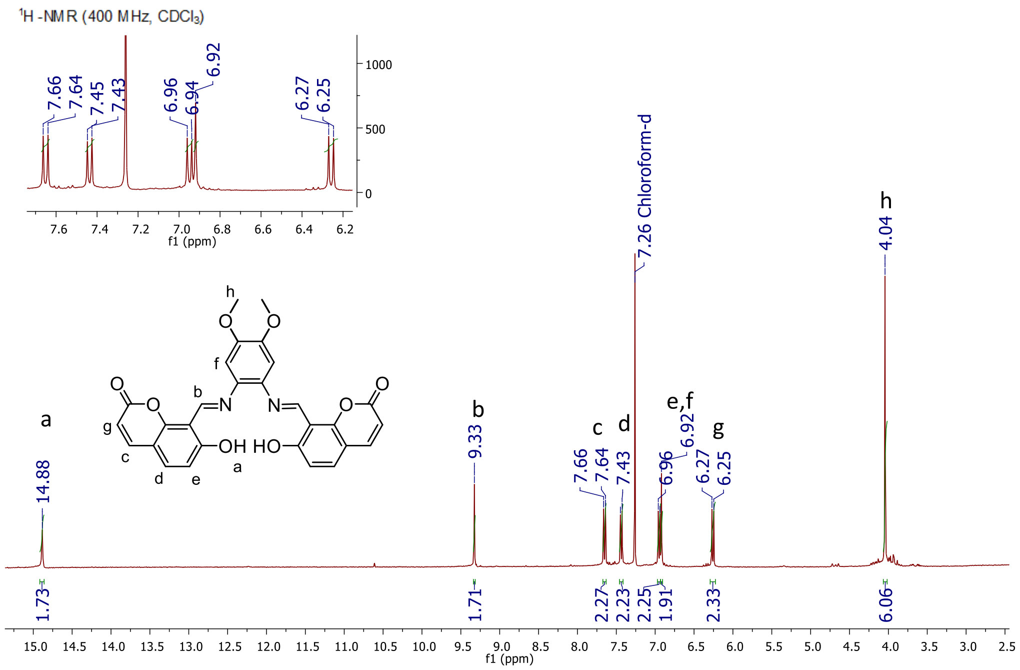

Figure S7. ^{1}H RMN spectrum of **L2** in $CDCl_3$.

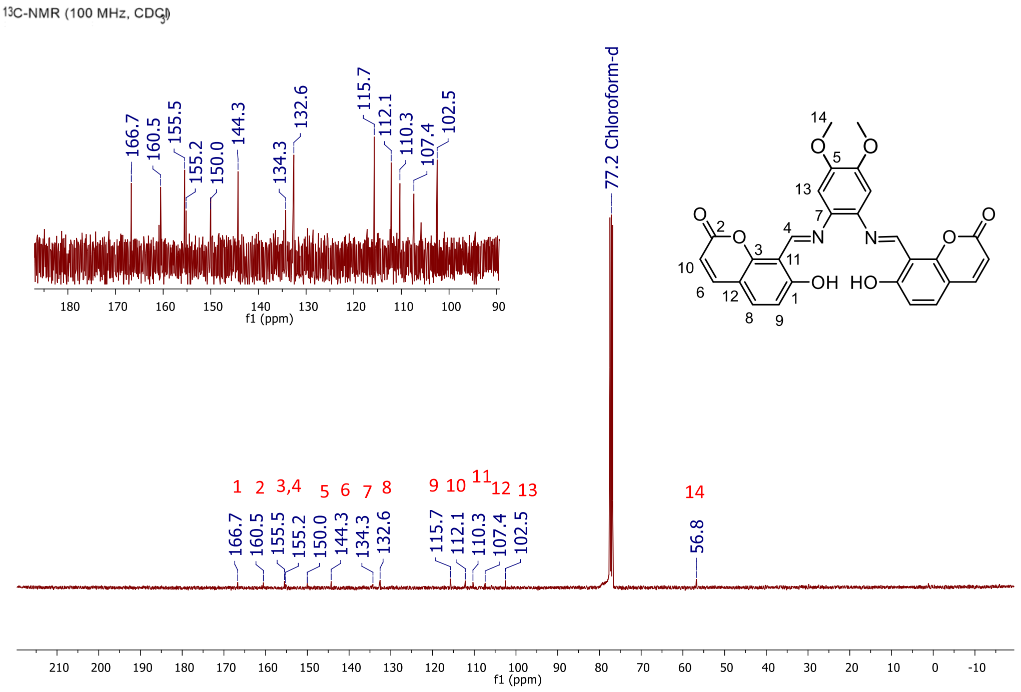

Figure S8. ^{13}C RMN spectrum of **L2** in $CDCl_3$.

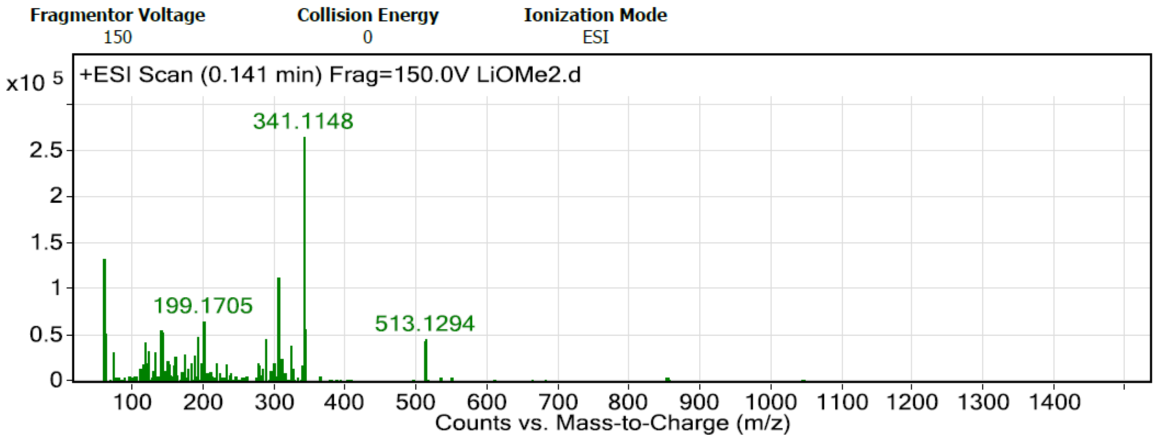

Figure S9. High-resolution mass spectrum (HRMS) of **L2** (solvent: acetonitrile).

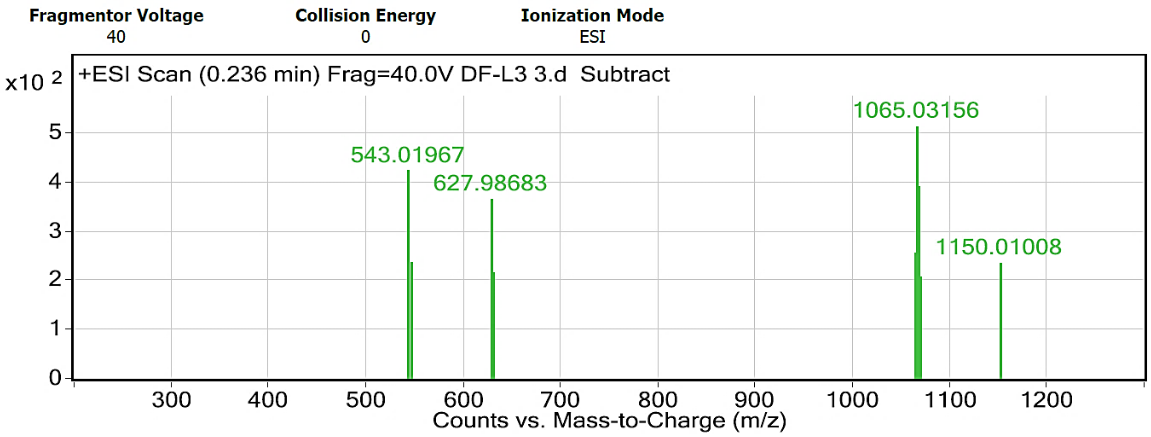

Figure S10. High-resolution mass spectrum (HRMS) of **L3** (solvent: acetonitrile).

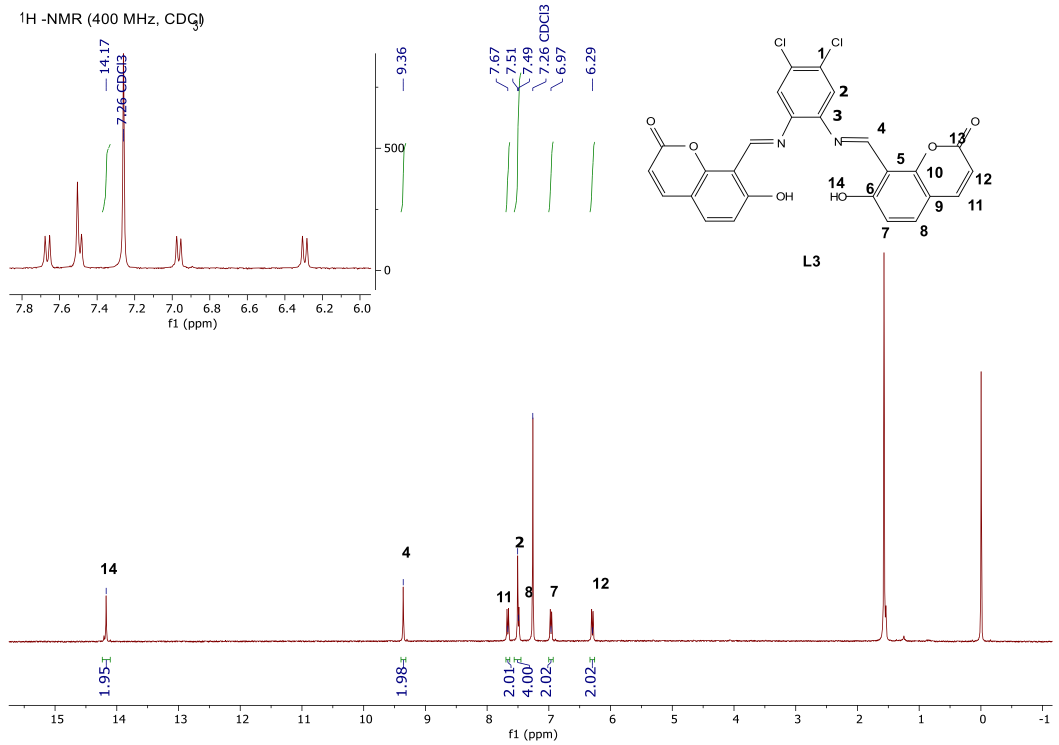

Figure S11. ^{1}H RMN spectrum of **L3** in $CDCl_3$.

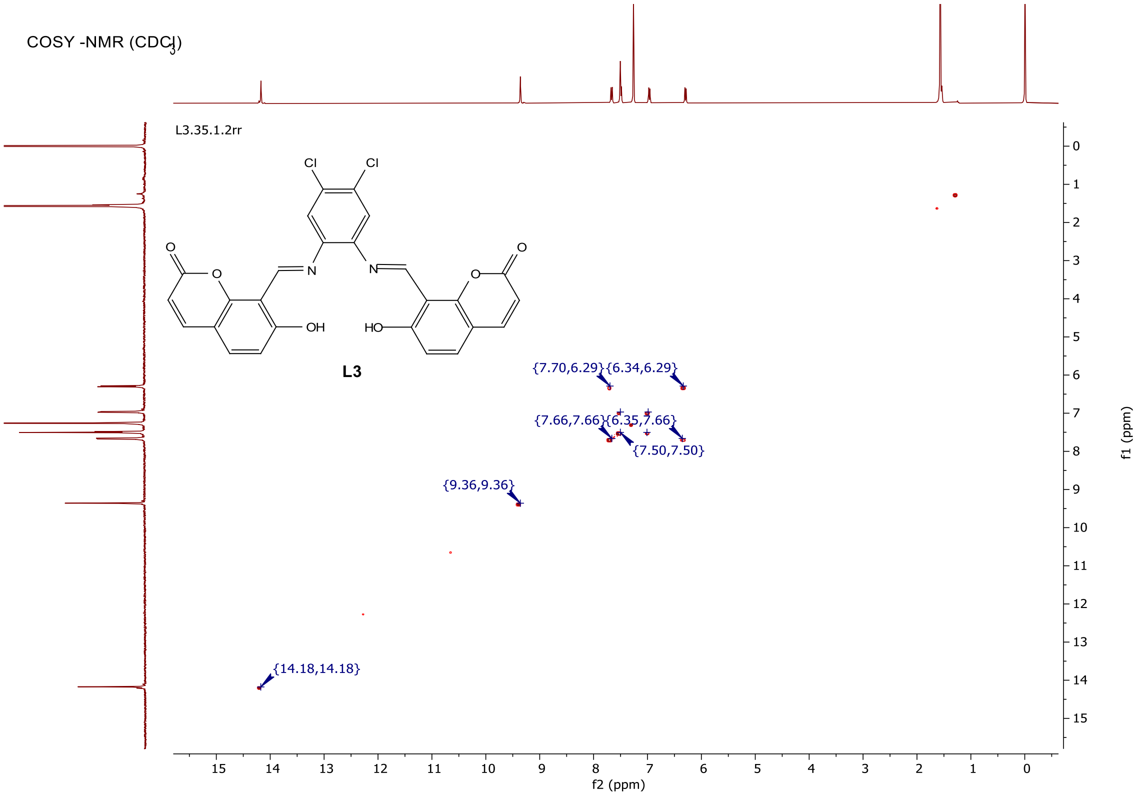

Figure S12. COSY spectrum of **L3** in $CDCl_3$.

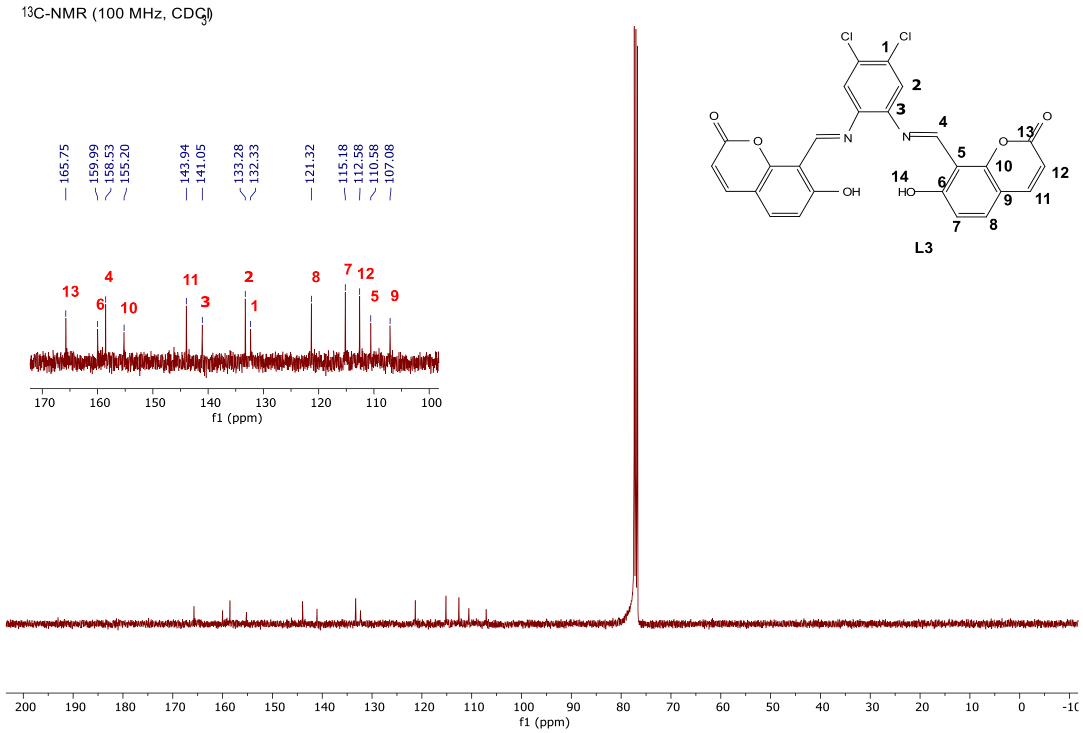

Figure S13 ^{13}C RMN spectrum **L3** in $CDCl_3$.

Since all the protons were initially assigned, the HSQC experiment (**Figure S14**) was used to determine the corresponding carbon to each proton, hence the 112.6, 115.2, 121.3, 133.3, 143.9, and 158.5 ppm signals can be associated with the carbons C12, C7, C8, C2, C11, and C4, respectively. Finally, the DEPT-135 (**Figure S15**) experiment was made to identify and assign quaternary carbons to the corresponding carbons (C9 = 107.1 ppm, C5 = 110.6 ppm, C1 = 132.3 ppm, C3 = 141.0 ppm, C10 = 155.2 ppm, C6 = 160.0 ppm, and C13 = 165.8 ppm).

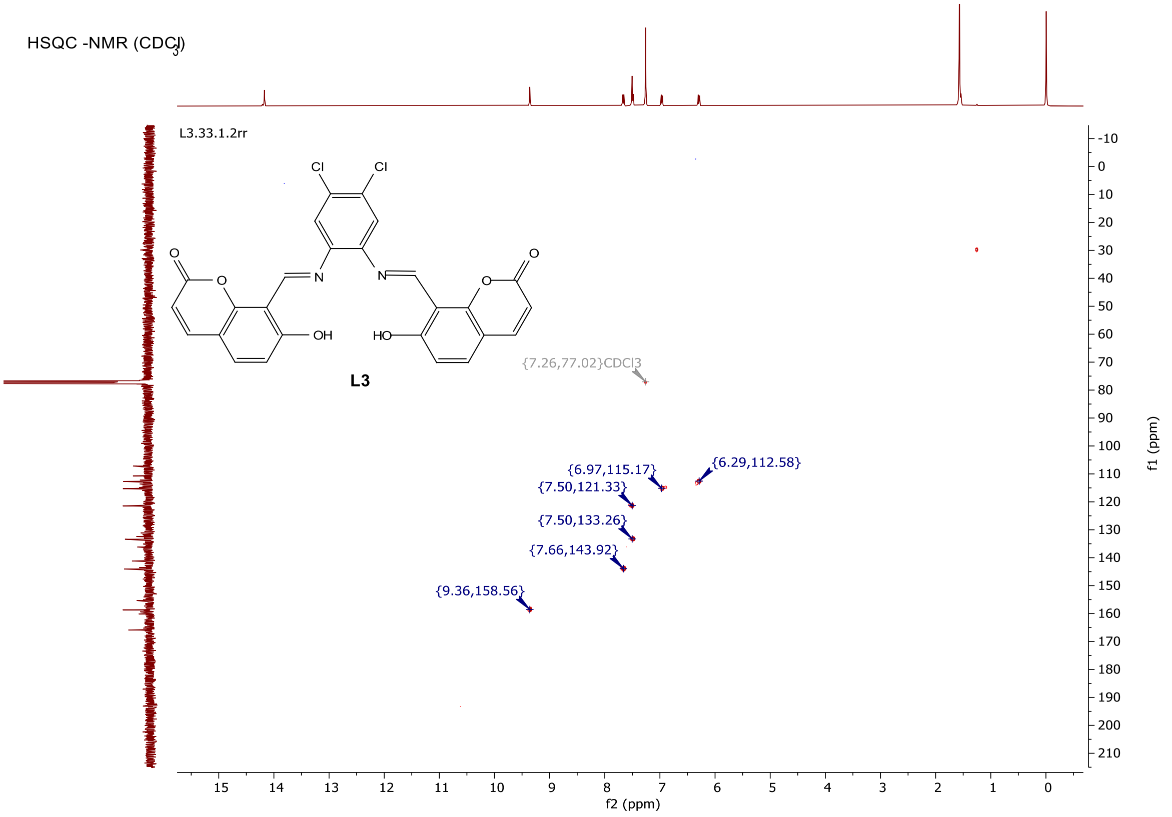

Figure S14. HSQC spectrum of **L3** in $CDCl_3$.

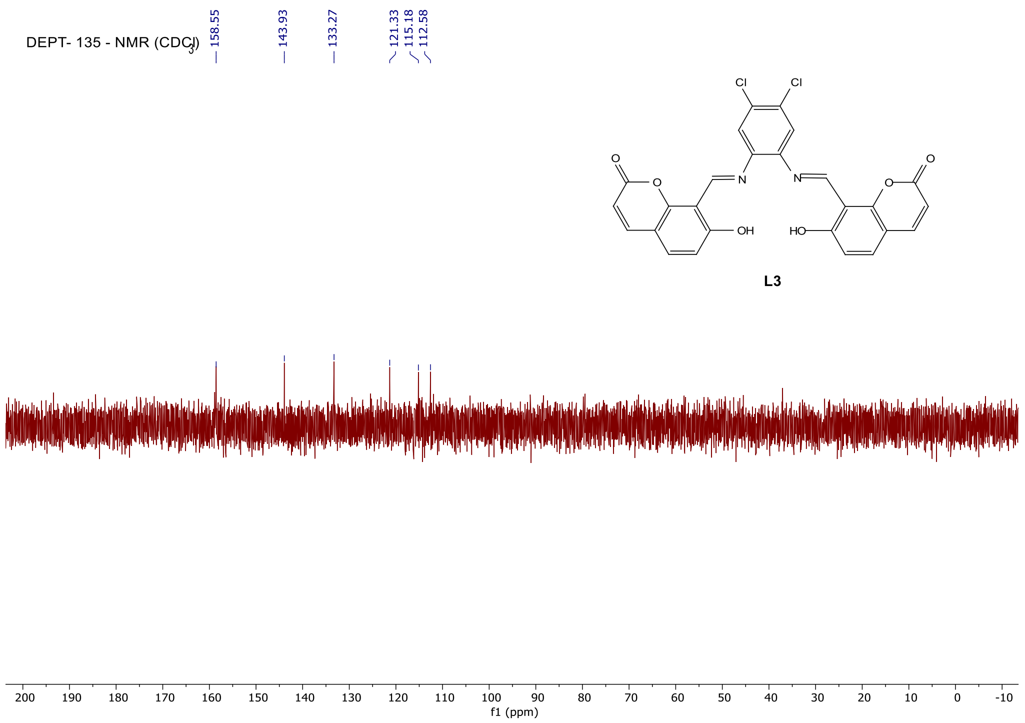

Figure S15. DEPT-135 spectrum of **L3** in $CDCl_3$.

The FTIR spectrum indicated a broad band between 3577 and 3350 cm^{-1} attributed to the υ(O–H) vibration of the phenyl group.[3,4] The carbonyl band υ(C=O) was observed in 1732 [5,6] and the υ(C=N) band at 1612 cm^{-1}.[7]

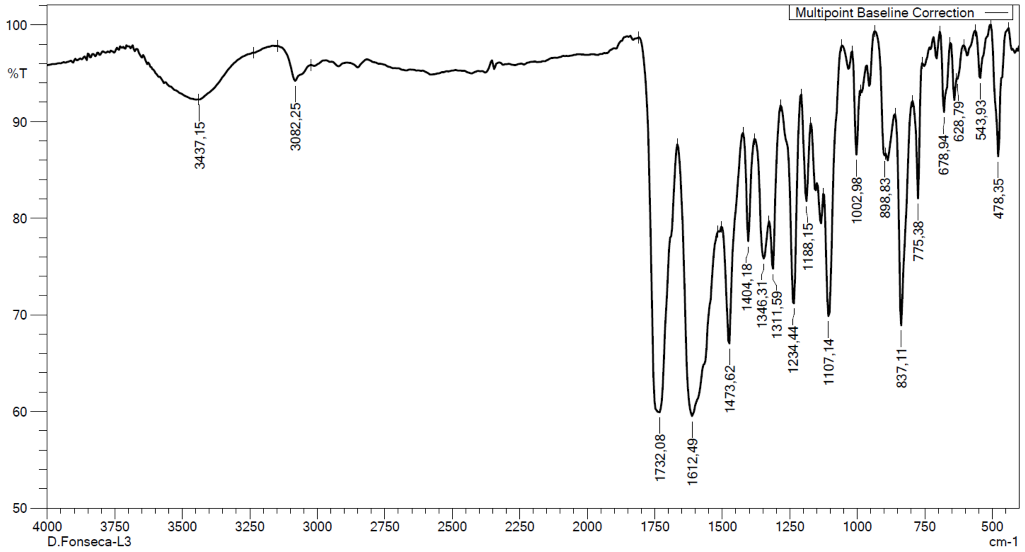

Figure S16. FTIR spectrum of **L3**.

The **L3** absorption electronic spectrum was obtained in DMSO (**Figure S17**). The band at 258 nm was assigned to the π-π* electronic transitions of the compound (C=C), i.e. intraligand transitions.[2,4] Another band observed in 316-350 nm as a shoulder was attributed to the π-π* electronic transition of the C=N portion. [8] Additionally, another shoulder band at 403-446 nm (C=O) corresponds to the promotion of an unpaired electron from the oxygen atom to an anti-bonding π orbital of the carbonyl group, corresponding to an n-π* electronic transition.[9]

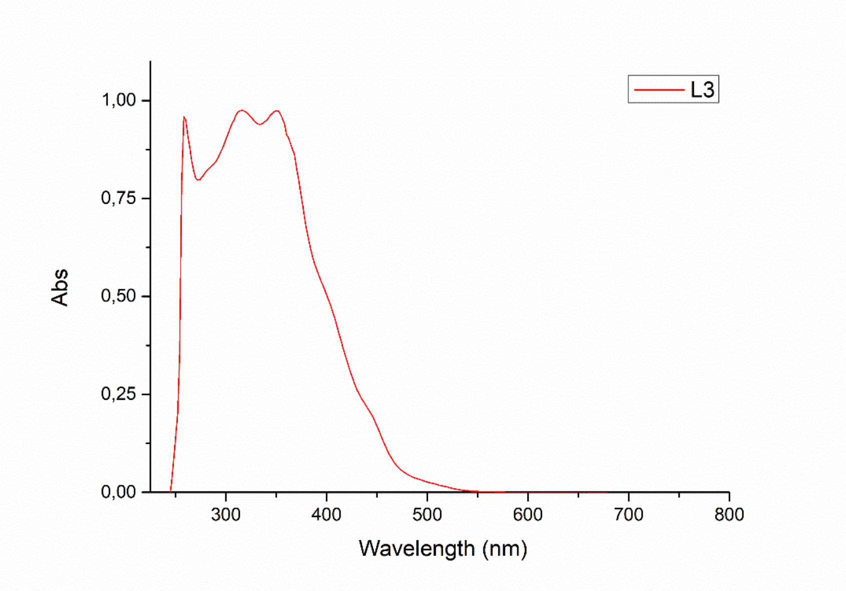

Figure S17. Absorption spectrum of **L3** in DMSO**.**

3. Catalytic results

3.1. Optimization of reaction conditions (A, L1-L3)

Table S1. Optimization of reaction conditions

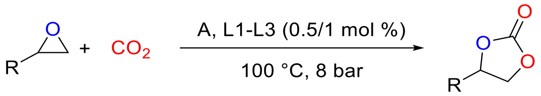

1c, 1k

2c, 2k

c: R= CH_2Cl, k: R= Ph

Organocatalysts		Entry	Time (h)	Epoxide	Catalyst (mol %)[c]	Conversion (%)[d]
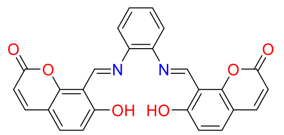	**L1**	1	24 [a]	**1c**	1	-
		2	24 [a]	**1k**	1	-
		3	24 [b]	**1c**	1	>99
		4	24 [b]	**1k**	1	62
		5	24 [b]	**1k**	0.5	35
		6	9 [b]	**1c**	1	>99
		7	9 [b]	**1c**	0.5	89
		8	9 [b]	**1k**	0.5	8
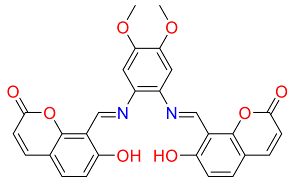	**L2**	9	24 [a]	**1c**	1	-
		10	24 [a]	**1k**	1	-
		11	24 [b]	**1c**	1	>99
		12	24 [b]	**1k**	1	74
		13	24 [b]	**1k**	0.5	48
		14	9 [b]	**1c**	1	>99
		15	9 [b]	**1c**	0.5	92
		16	9 [b]	**1k**	0.5	12
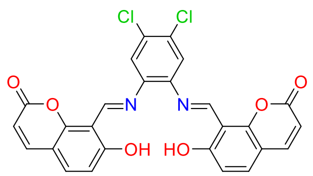	**L3**	17	24 [a]	**1c**	1	-
		18	24 [a]	**1k**	1	-
		19	24 [b]	**1c**	1	>99
		20	24 [b]	**1k**	1	70
		21	24 [b]	**1k**	0.5	30
		22	9 [b]	**1c**	1	>99
		23	9 [b]	**1c**	0.5	62
		24	9 [b]	**1k**	0.5	6
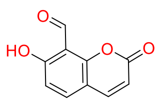	**A**	25	24 [a]	**1c**	1	-
		26	24 [a]	**1k**	1	-
		27	24 [b]	**1c**	1	>99
		28	24 [b]	**1k**	1	56
		29	24 [b]	**1k**	0.5	23
		30	9 [b]	**1c**	1	>99
		31	9 [b]	**1c**	0.5	42
		32	9 [b]	**1k**	0.5	5

[a] r.t, 1 bar CO_2 pressure under free-solvent conditions. [b] 100 °C, 8 bar CO_2 pressure under free-solvent conditions. [c] Epoxide (0.11 mL, 1.40 mmol (**1c**) and 1 mmol (**1k**)), catalyst (1.406 x10^{-5} mol (1 %) and 7.032 x10^{-6} mmol (0.5 mol %) concerning the initial amount of epoxide). [d] Determined by ^{1}H NMR spectroscopy of the crude reaction mixture.

3.2. TON and TOF catalytic results

Table S2. TON and TOF catalytic results.

Entry	Epoxide	Organocat.	Conv. (%)	TON	TOF (h^{-1})
**1a**	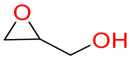	**L1**	99	112	12
		**L2**	99	112	12
**1b**	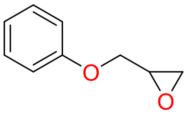	**L1**	99	198	22
		**L2**	99	198	22
**1c**	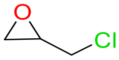	**L1**	89	178	20
		**L2**	92	184	20
**1d**	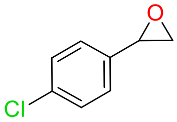	**L1**	81	162	18
		**L2**	85	170	19
**1e**	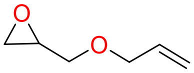	**L1**	54	108	12
		**L2**	60	120	13.3
**1f**	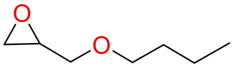	**L1**	47	94	10.4
		**L2**	50	100	11.1
**1g**	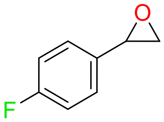	**L1**	40	56	6.2
		**L2**	50	70	7.8
**1h**	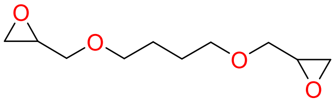	**L1**	30	60	6.7
		**L2**	31	62	6.9
**1i**	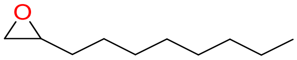	**L1**	11	25	2.7
		**L2**	13	29	3.2
**1j**	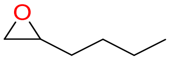	**L1**	6	12	1.3
		**L2**	20	38	4.2
**1k**	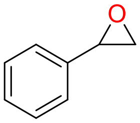	**L1**	8	16	1.2
		**L2**	12	24	2.7
**1l**	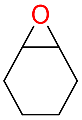	**L1**	67	134	5.6
		**L2**	72	144	6

3.3. Characterization of cyclic carbonates by ^{1}H and ^{13}C NMR

Glycerol carbonate (2a). Obtained as a colorless liquid. **^{1}H NMR** (400 MHz, $CDCl_3$) 4.84–4.76 (1H, m, OCH), 4.50 (1H, t, *J* = 8.3 Hz, CH_2O), 4.29 (1H, dd, *J* = 10,2, 5,1 Hz, CH_2O), 3.70–3.64 (1H, m, CH_2OH), 3.54–3.48 (1H, m, CH_2OH); **^{13}C NMR** (400 MHz, $CDCl_3$) δ 155.30, 76.60, 65.85, 61.86.

Phenylglycidylether Carbonate (2b). Purification by flash column chromatography with hexane: EtOAc (8:2). **^{1}H NMR** (400 MHz, $CDCl_3$) δ 7.35 – 7.28 (m, 2H, 2OArH), 7,02 (t, *J*= 7,5 Hz, 1H, ArH), 6.94–6.88 (m, 2H, 2OArH), 5.07 – 4.98 (m, 1H, OCH), 4,61 (t, *J*= 8.5 Hz, 1H, OCH2), 4,53 (dd, *J*= 8.5, 5.9 Hz, 1H, OCH_2), 4,24 (dd, *J*= 10.6, 4.3 Hz, 1H, CH2OPh), 4.15 (dd, *J*= 10.6, 3.6 Hz, 1H, CH_2OPh); **^{13}C NMR** (400 MHz, $CDCl_3$) δ 157.58, 154.45, 129.52, 121.83, 114.44, 73.91, 66.71, 66.06.

3-Chloropropylene Carbonate (2c). Purification by flash column chromatography with hexane: EtOAc (6:4). **^{1}H NMR** (400 MHz, $CDCl_3$) δ 5.01-4.93 (1H, m, OCH), 4.57 (1H, t, J = 8.5 Hz, OCH_2), 4.42 (1H, t, J = 8.5, OCH_2), 3.70-3.81 (2H, m, CH_2Cl); **^{13}C NMR** (400 MHz, $CDCl_3$) δ 154.41, 74.45, 67.00, 43.96.

4-Chlorostyrene Carbonate (2d). Purification by flash column chromatography with hexane: EtOAc (6:4). **^{1}H NMR** (400 MHz, $CDCl_3$) δ 7.45 – 7.42 (m, 2H, ArH), 7.38 – 7.33 (m, 2H, ArH), 5.67 (t, *J* = 8.0 Hz, 1H, OCH), 4.79 (t, *J* = 8.4 Hz, 1H, OCH), 4.37 – 4.29 (t, *J* = 8.4 Hz, 1H, OCH_2); **^{13}C NMR** (400 MHz, $CDCl_3$) δ 154.93, 135.91, 129.80, 129.31, 125.97, 78.08, 71.25.

Allyl glycidyl ether Carbonate (2e). Purification by flash column chromatography with hexane: EtOAc (8:2). **^{1}H NMR** (400 MHz, $CDCl_3$)δ 5.94 – 5.80 (m, 1H, CH), 5.32 – 5.18 (m, 2H, CH_2), 4.81 (ddd, *J* = 6.4, 3.6, 1.8 Hz, 1H, CH), 4.53 – 4.44 (m, 1H, CH), 4.43 – 4.35 (m, 1H, CH), 4.06 (ddt, *J* = 5.8, 3.1, 1.5 Hz, 2H, CH_2), 3.74 – 3.55 (m, 2H, CH_2); **^{13}C NMR** (400 MHz, $CDCl_3$) δ 155.06, 133.80, 118.12, 75.16, 72.77, 68.99, 66.42.

Butyl glycidyl ether Carbonate (2f). Purification by flash column chromatography with hexane: EtOAc (8:2). **^{1}H NMR** (400 MHz, $CDCl_3$)δ 4.80 (ddq, *J* = 8.1, 6.1, 4.3 Hz, 1H, CH), 4.48 (t, *J* = 8.3 Hz, 1H, CH), 4.38 (dd, *J* = 8.3, 6.1 Hz, 1H, CH), 3.70 – 3.56 (m, 2H, CH_2), 3.55 – 3.43 (m, 2H, CH_2), 1.55 (dq, *J* = 8.5, 6.8 Hz, 2H, CH_2), 1.45 – 1.28 (m, 2H, CH_2), 0.91 (t, *J* = 7.3 Hz, 3H, CH_3); **^{13}C NMR** (400 MHz, $CDCl_3$) δ 155.17, 75.28, 71.91, 69.68, 66.38, 31.56, 19.18, 13.86.

4-Fluorostyrene Carbonate (2g). Purification by flash column chromatography with hexane: EtOAc (8:2). **^{1}H NMR** (400 MHz, $CDCl_3$) δ 7.45 – 7.36 (m, 2H, ArH), 7.33 – 7.27 (m, 2H, ArH), 5.65 (t, *J* = 7.9 Hz, 1H, OCH), 4.80 (t, *J* = 8.4 Hz, 1H, OCH), 4.34 – 4.21 (t, *J* = 8.4 Hz, 1H, OCH_2). **^{13}C NMR** (400 MHz, $CDCl_3$) δ 154.47, 135.66, 134.24, 129.41, 127.21, 76.66, 70.91.

1,4-butanediol diglycidyl ether Carbonate (2h). Purification by flash column chromatography with hexane: EtOAc (6:4). **^{1}H NMR** (400 MHz, $CDCl_3$)δ 4.79 (ddt, *J* = 8.0, 6.0, 3.9 Hz, 2H, 2 x CH), 4.49 (t, *J* = 8.3 Hz, 2H, CH_2), 4.39 (dd, *J* = 8.3, 6.1 Hz, 2H, CH_2), 3.68 – 3.59 (m, 4H, 2 x CH_2), 3.51 (t, *J* = 6.5 Hz, 4H, 2 x CH_2), 1.36 (dq, *J* = 14.6, 7.3 Hz, 4H, 2 x CH_2);); **^{13}C NMR** (400 MHz, $CDCl_3$) δ 155.21, 75.43, 71.36, 69.58, 66.21, 25.91.

1,2-Decylene Carbonate (2i). Purification by flash column chromatography with hexane: EtOAc (6:4). **^{1}H NMR** (400 MHz, $CDCl_3$) δ 4.71−4.68 (1H, m, OCH), 4.52 (1H, t, J = 8.0 Hz, OCH2), 4.06 (1H, t, J = 8.0 Hz, OCH_2), 1.84 – 1.64 (m, 2H, CH_2), 1.47−1.26 (12H, m, 6 × CH_2), 0.91 – 0.85 (m, 3H, CH_3). **^{13}C NMR** (400 MHz, $CDCl_3$) δ 155.20, 77.19, 69.52, 34.04, 32.01, 29.66, 29.58, 29.28, 24.50, 22.80, 14.23.

1,2-Hexylene Carbonate (2j). Purification by flash column chromatography with hexane: EtOAc (6:4). ^{1}H NMR (400 MHz,) δ 4.70 (qd, *J* = 7.5, 5.4 Hz, 1H, CH), 4.52 (t, *J* = 8.2 Hz, 1H, CH), 4.06 (dd, *J* = 8.5, 7.1 Hz, 1H, CH), 1.89 – 1.62 (m, 2H, CH_2), 1.48 – 1.29 (m, 4H, 2 X CH_2), 0.92 (t, *J* = 7.1 Hz, 3H, , CH_3). **^{13}C NMR** (400 MHz, $CDCl_3$) δ 154.95, 76.94, 69.18, 33.08, 26.09, 21.88, 13.39.

Styrene Carbonate (2k). Purification by flash column chromatography with hexane: EtOAc (6:4). **^{1}H NMR** (400 MHz, $CDCl_3$) δ 7.41-7.29 (5H, m, Ph), 5.68 (t, 1H, J = 8.5 Hz, OCH), 4.80 (t, 1H, J = 8.5, OCH_2), 4.35 (t, 1H, J = 8.5, OCH_2); **^{13}C NMR** (400 MHz, $CDCl_3$) δ 154.66, 135.74, 134.39, 129.51, 127.39, 77.33, 71.07.

***cis*-1,2-cyclohexane carbonate (2l).** Purification by flash column chromatography with hexane: EtOAc (6:4). **^{1}H NMR** (400 MHz, $CDCl_3$)δ 4.55 (s, 1H, CHO), 1.94–1.85 (m, 2H, 2 X CH_2), 1.68–1.57 (m, 1H, CH_2), 1.46–1.37 (m, 1H, CH_2); **^{13}C NMR** (400 MHz, $CDCl_3$) δ 155.01, 75.42, 2620, 18.63.

3.4. ^{1}H and ^{13}C NMR carbonate cyclic spectra

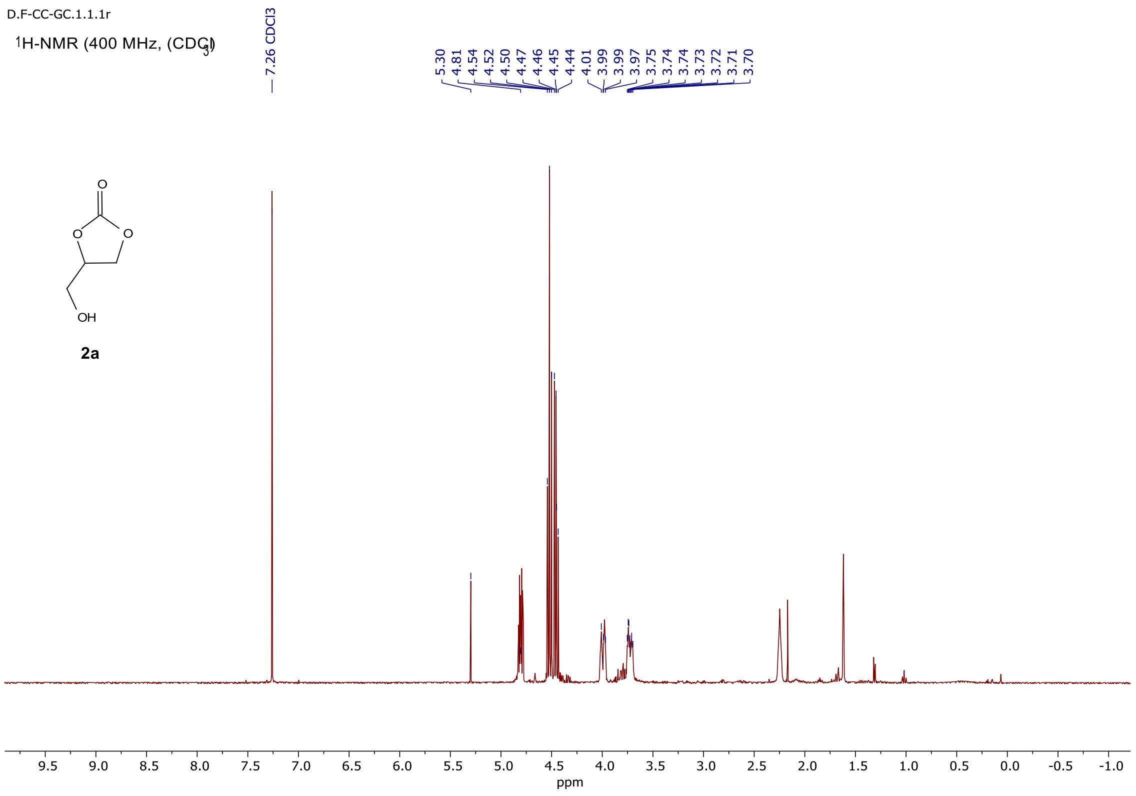

Figure S18. Glycerol carbonate **2a**: ^{1}H NMR spectrum in $CDCl_3$.

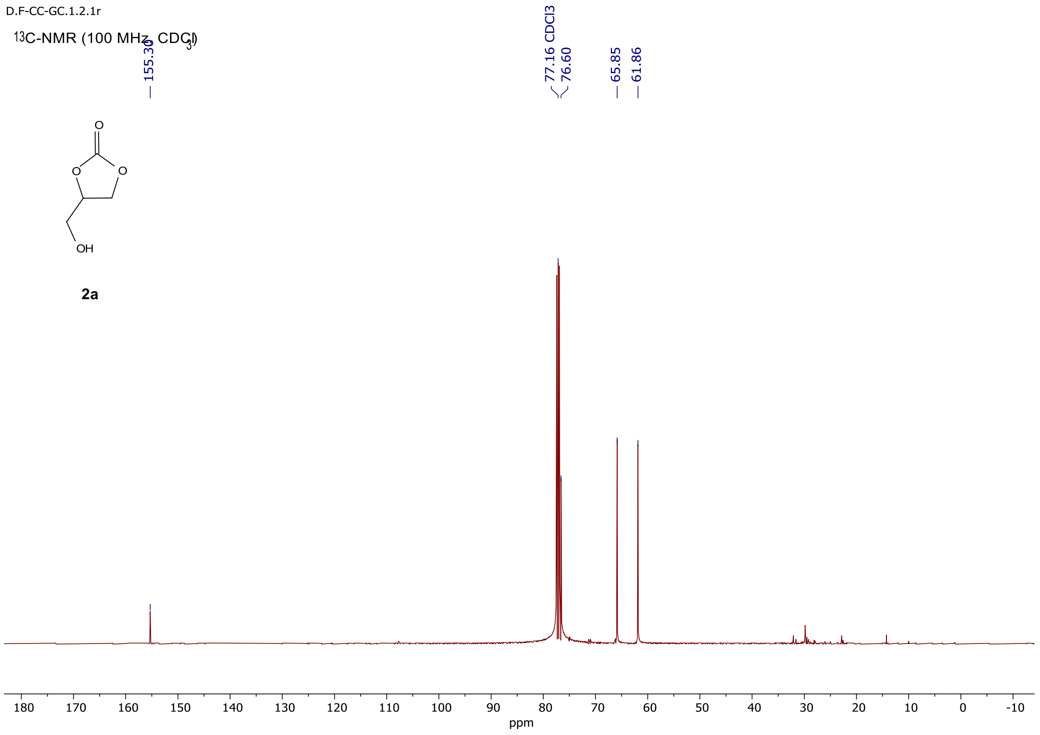

Figure S19. Glycerol carbonate **2a**: ^{13}C NMR spectrum in $CDCl_3$

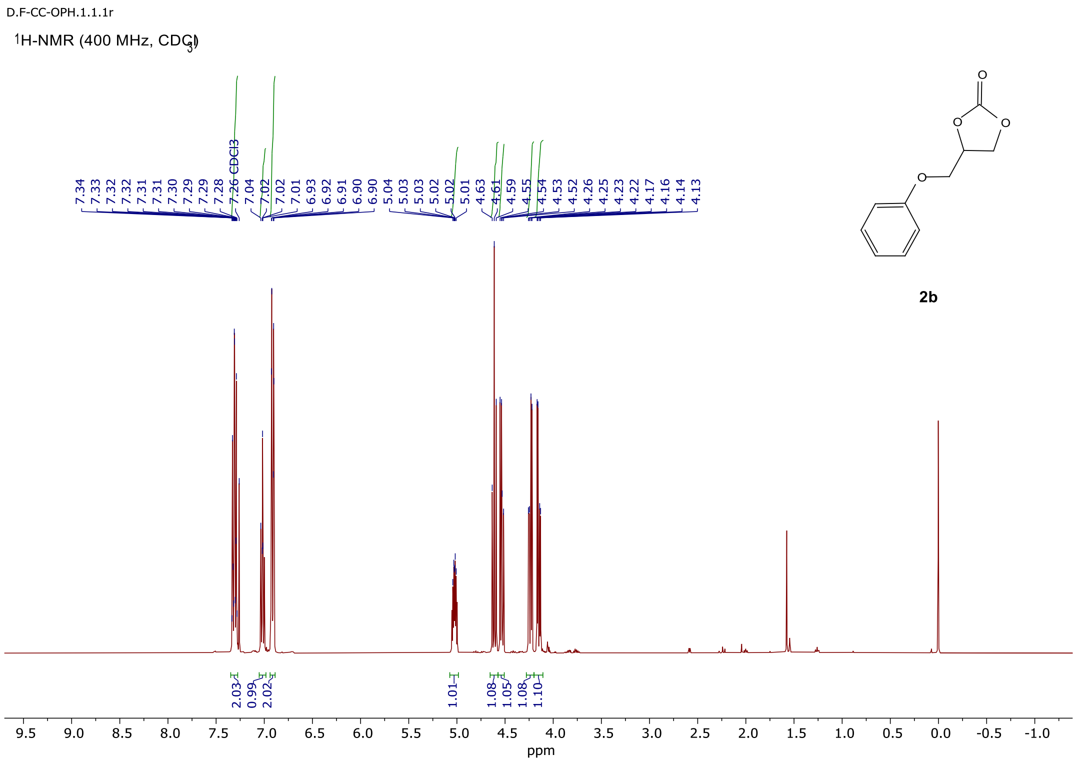

Figure S20. Phenyl glycidyl ether carbonate **2b**: ^{1}H NMR spectrum in $CDCl_3$.

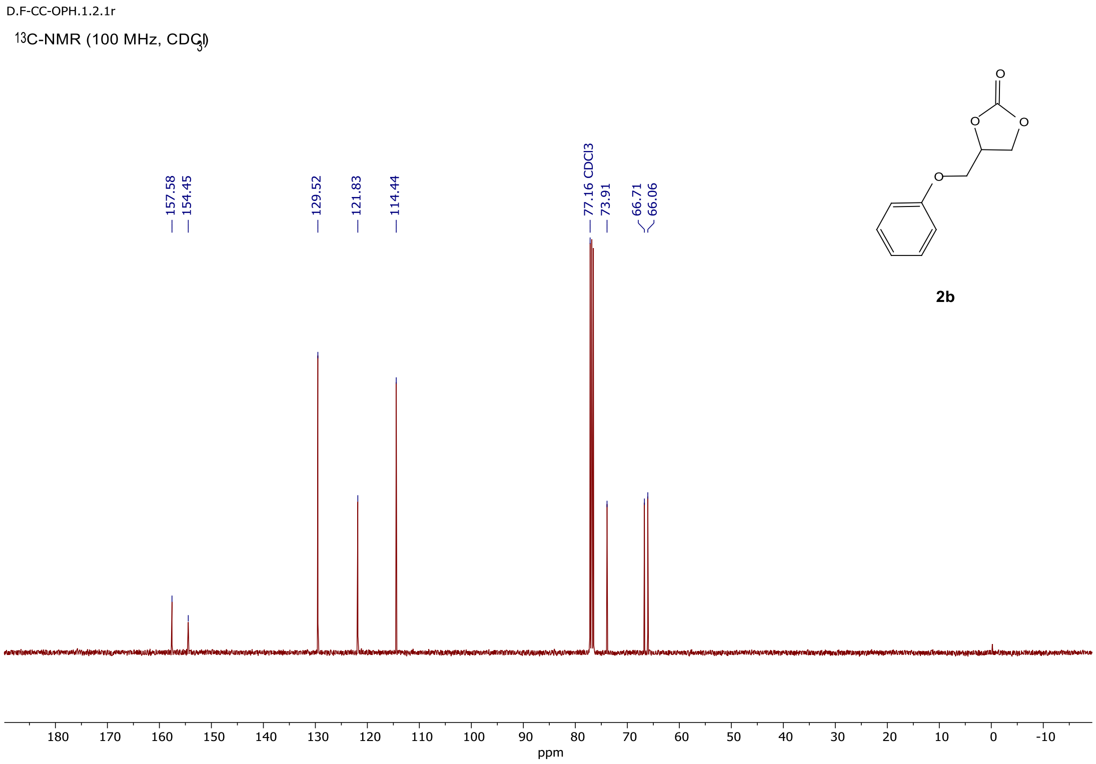

Figure S21. Phenyl glycidyl ether carbonate **2b**: ^{13}C NMR spectrum in $CDCl_3$.

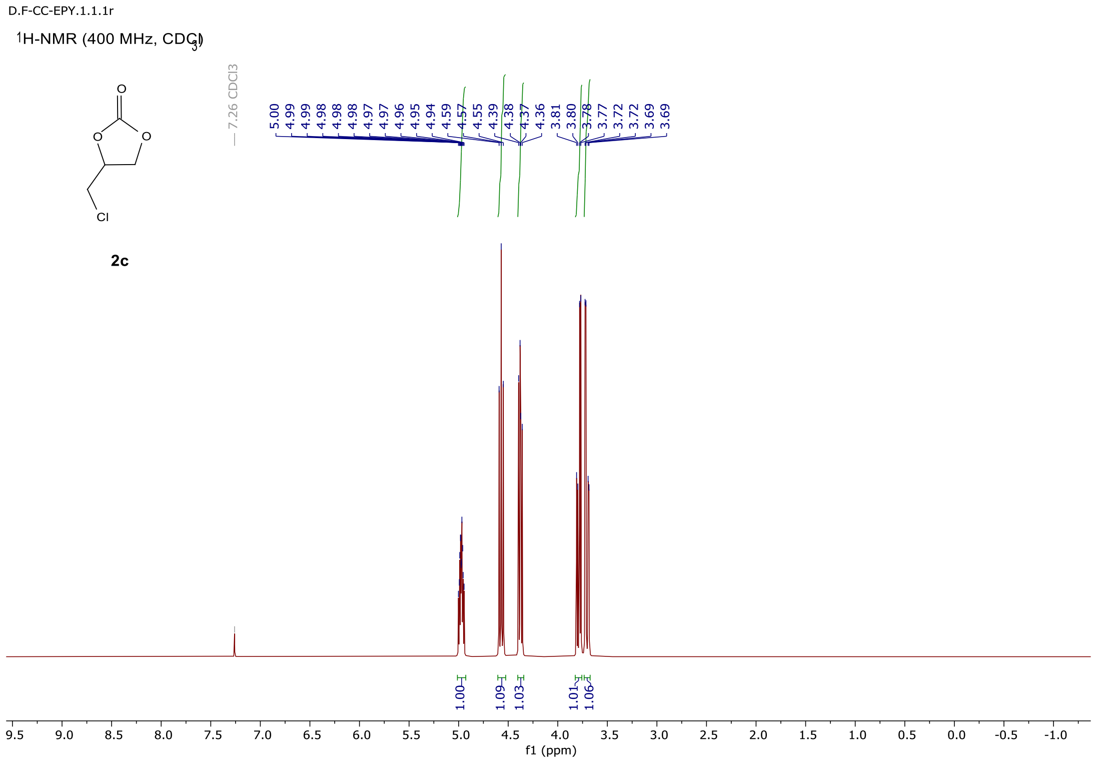

Figure S22. 3-Chloropropylene carbonate **2c**: ^{1}H NMR spectrum in $CDCl_3$.

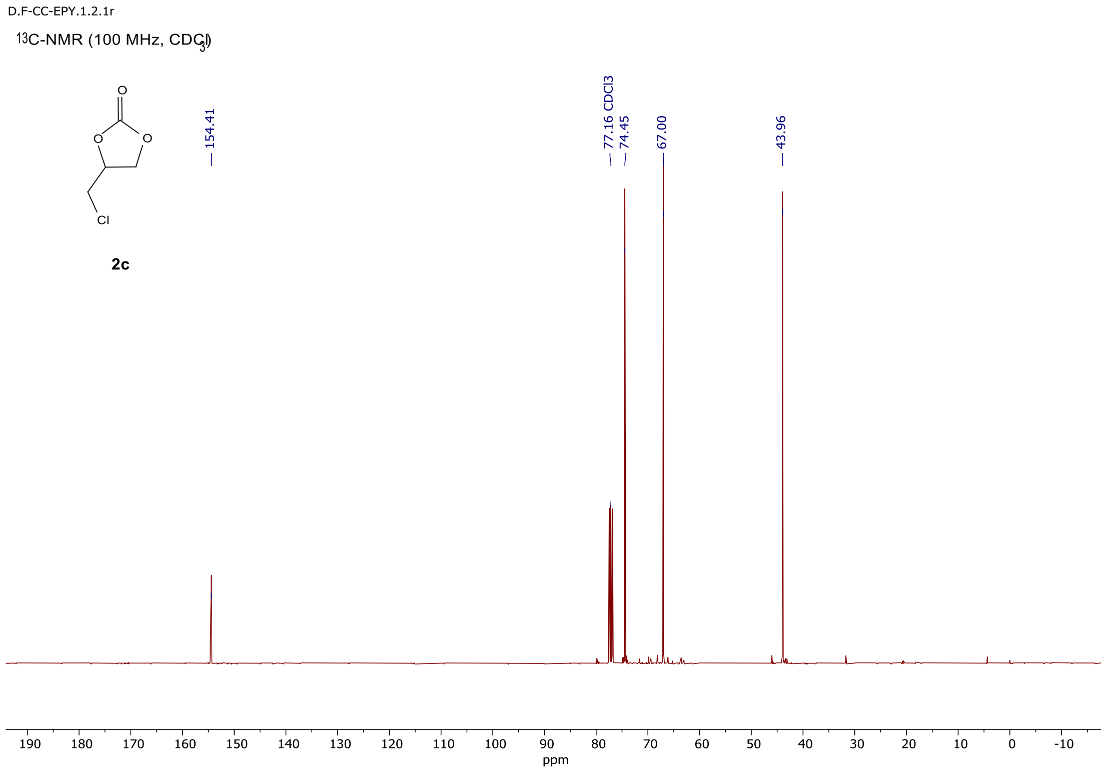

Figure S23. 3-Chloropropylene carbonate **2c**: ^{13}C NMR spectrum in $CDCl_3$.

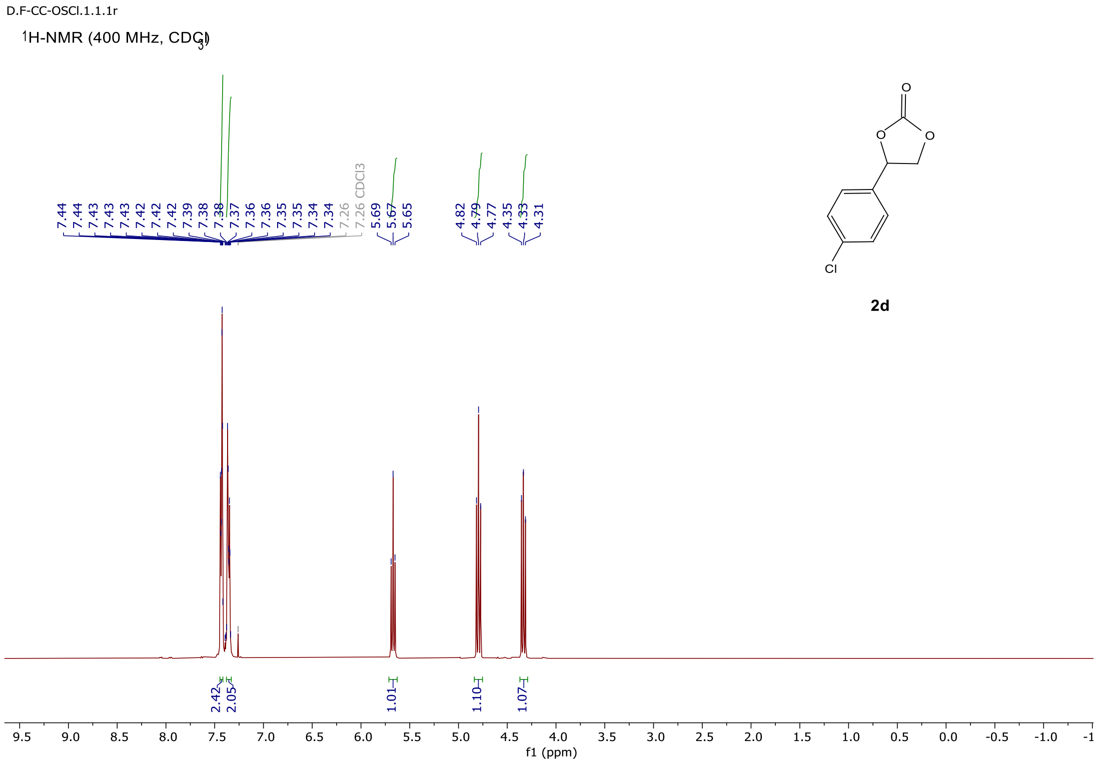

Figure S24. 4-Chlorostyrene carbonate **2d**: ^{1} H NMR spectrum in $CDCl_3$.

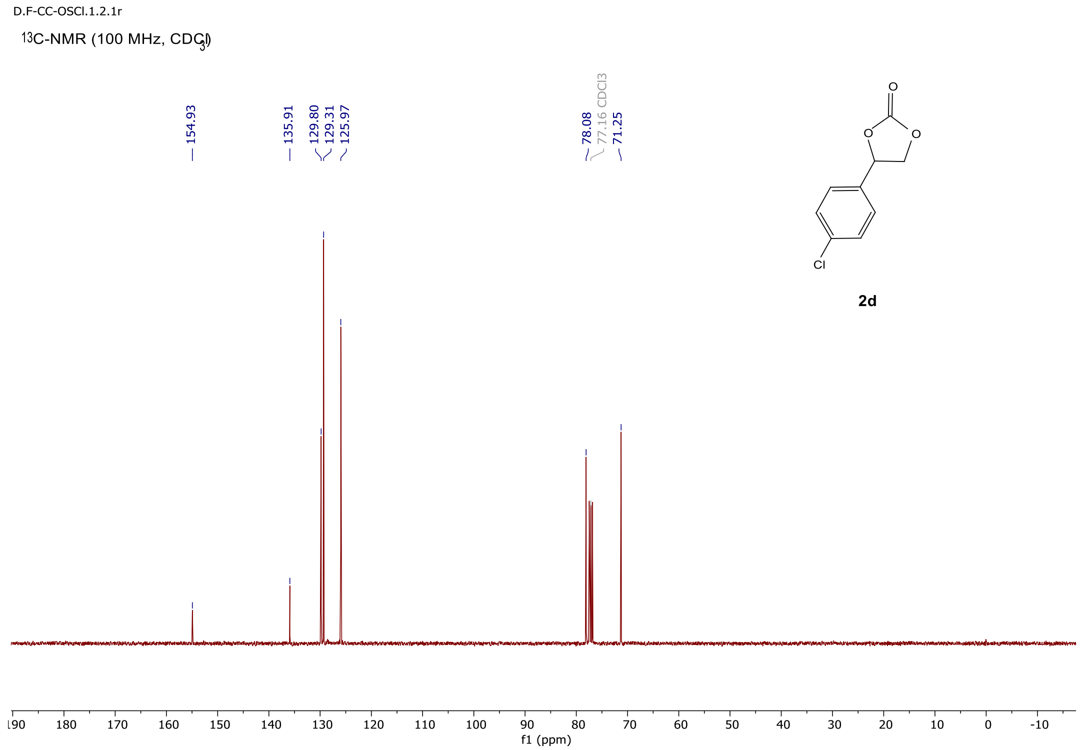

Figure S25. 4-Chlorostyrene carbonate **2d**: ^{13} C NMR spectrum in $CDCl_3$.

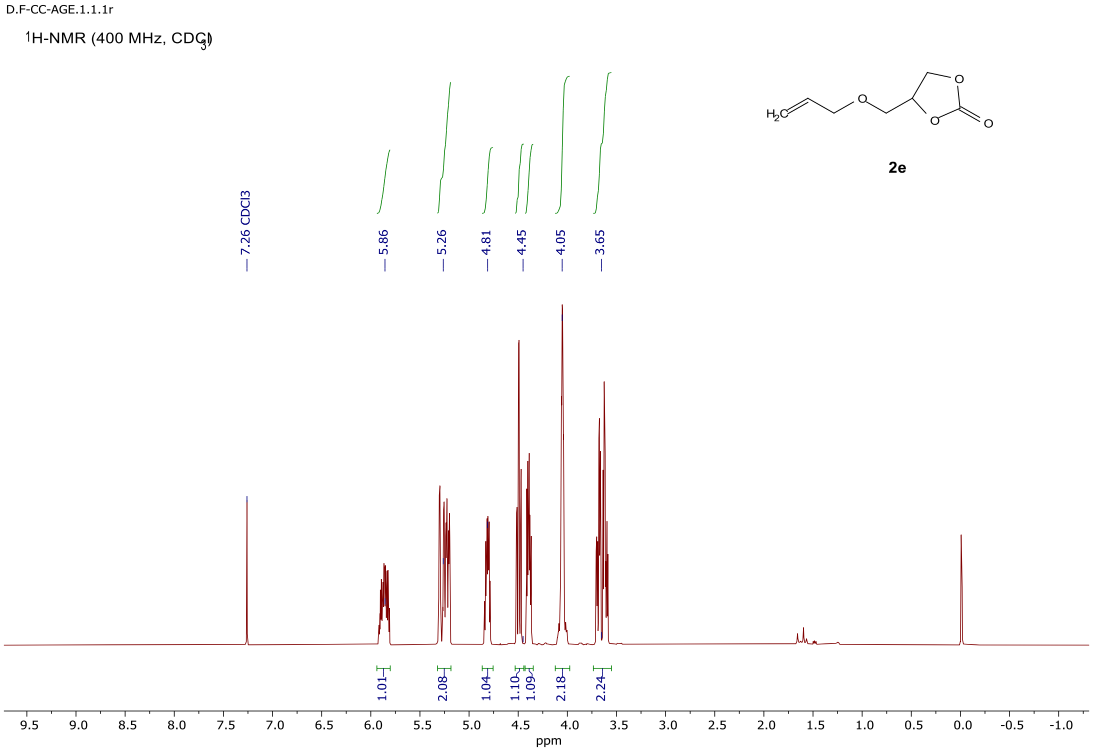

Figure S26. Allyl glycidyl ether carbonate **2e**: ^{1}H NMR spectrum in $CDCl_3$.

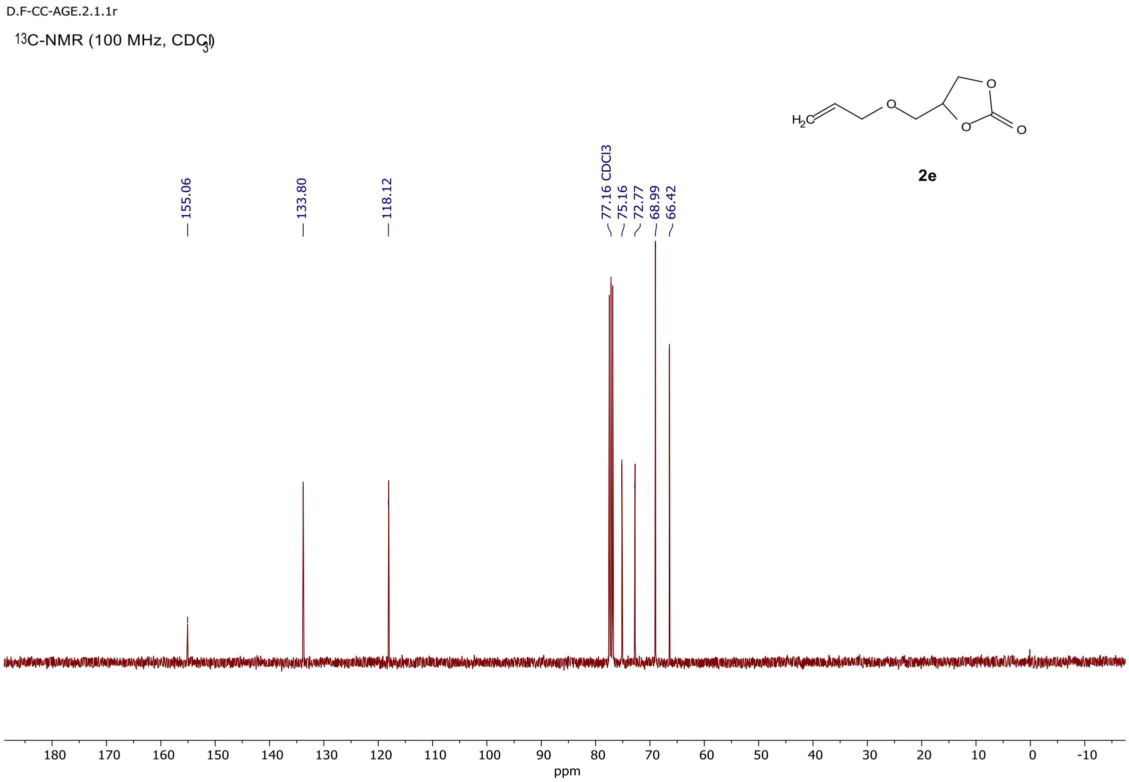

Figure S27. Allyl glycidyl ether carbonate **2e**: ^{13}C NMR spectrum in $CDCl_3$.

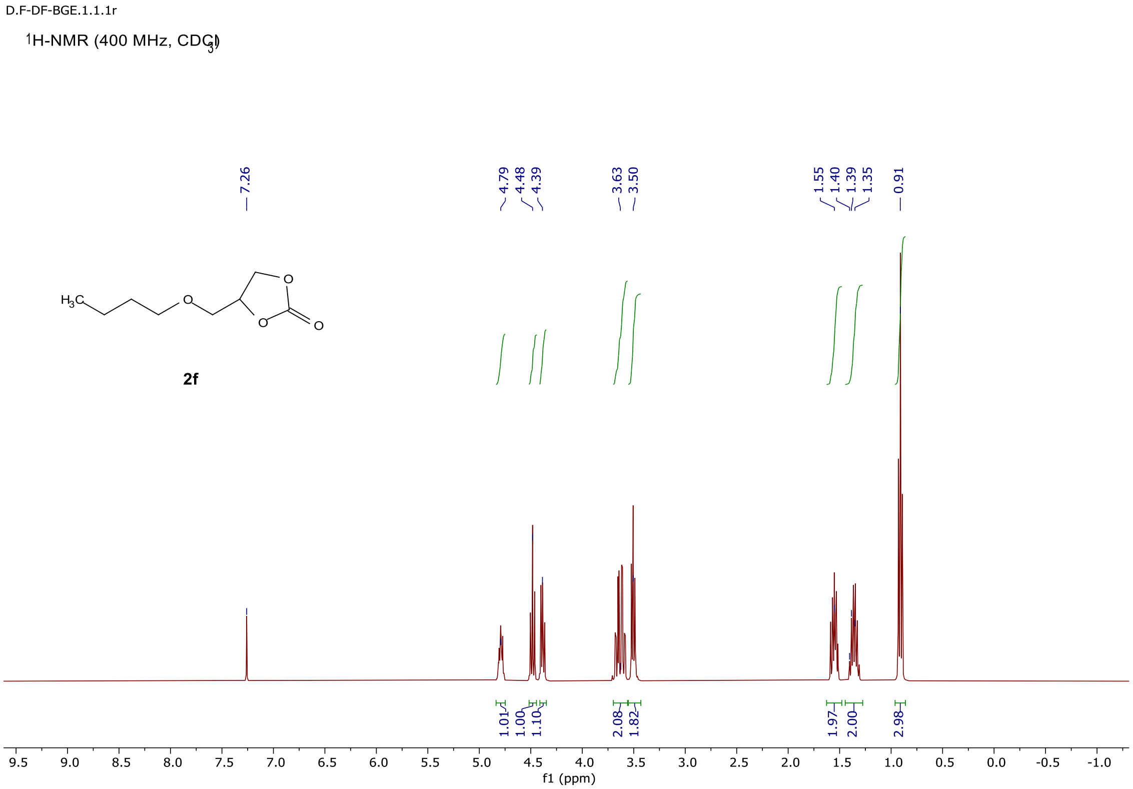

Figure S28. Butyl glycidyl ether carbonate **2f**: ^{1}H NMR spectrum in $CDCl_3$.

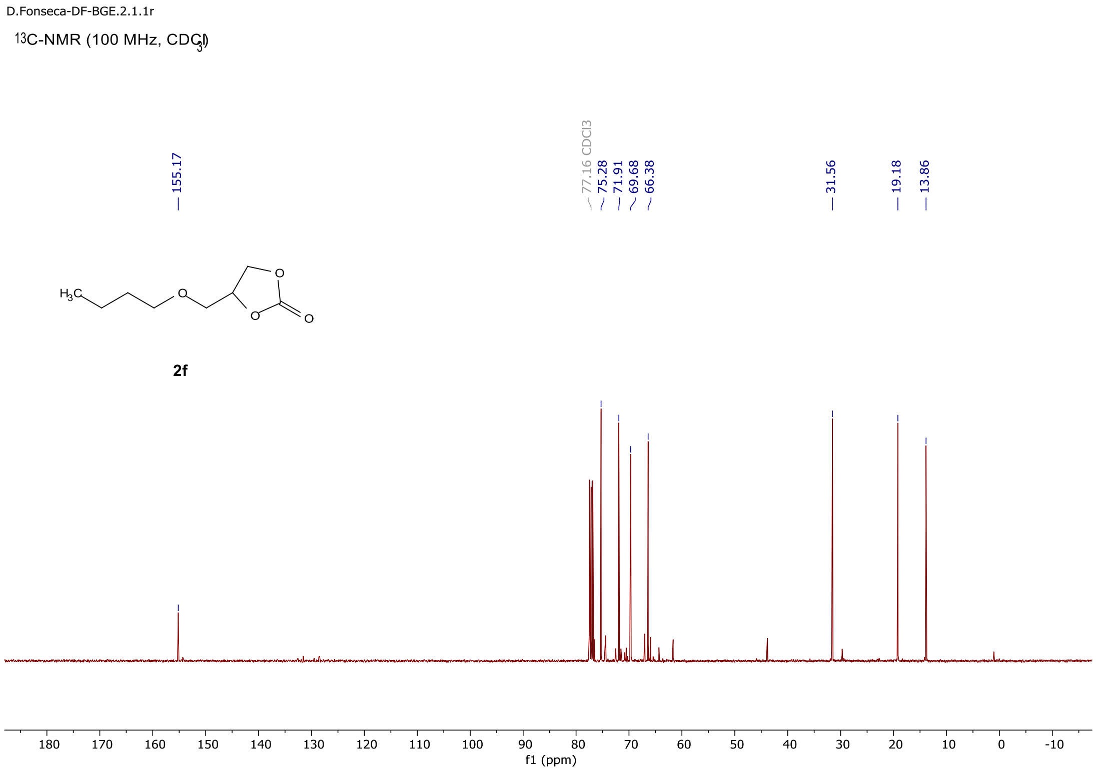

Figure S29. Butyl glycidyl ether carbonate **2f**: ^{13}C NMR spectrum in $CDCl_3$.

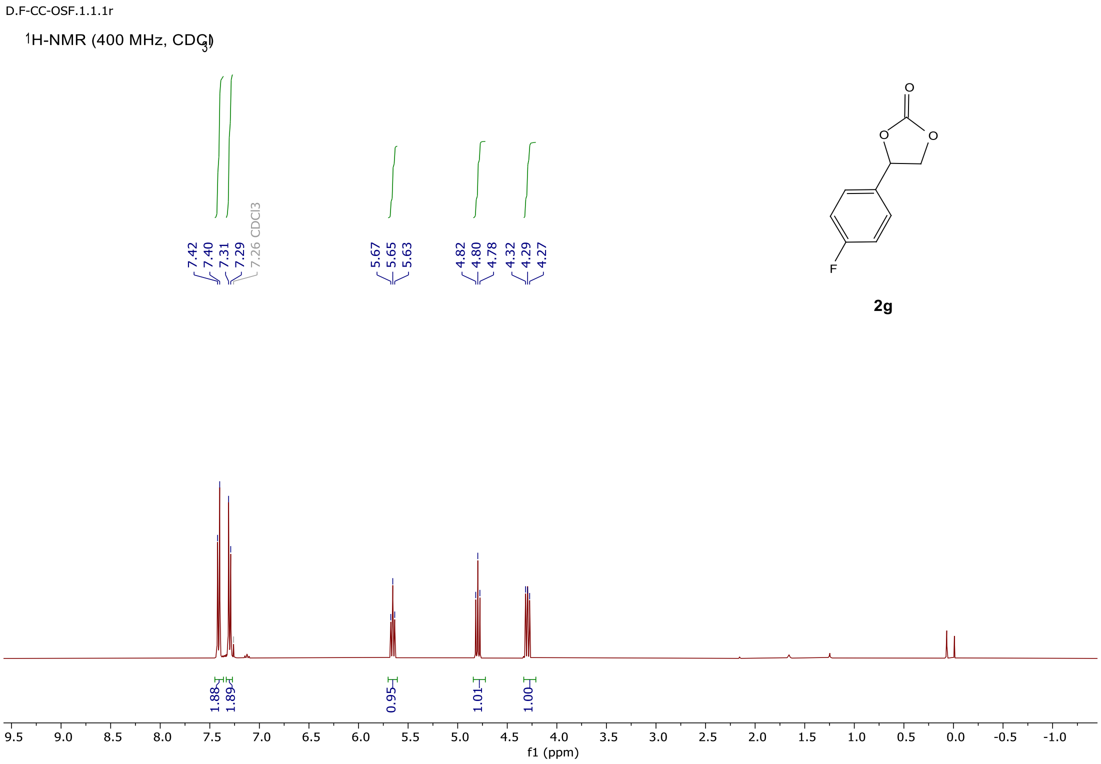

Figure S30. 4-Fluorostyrene carbonate **2g**: ^{1}H NMR spectrum in $CDCl_3$.

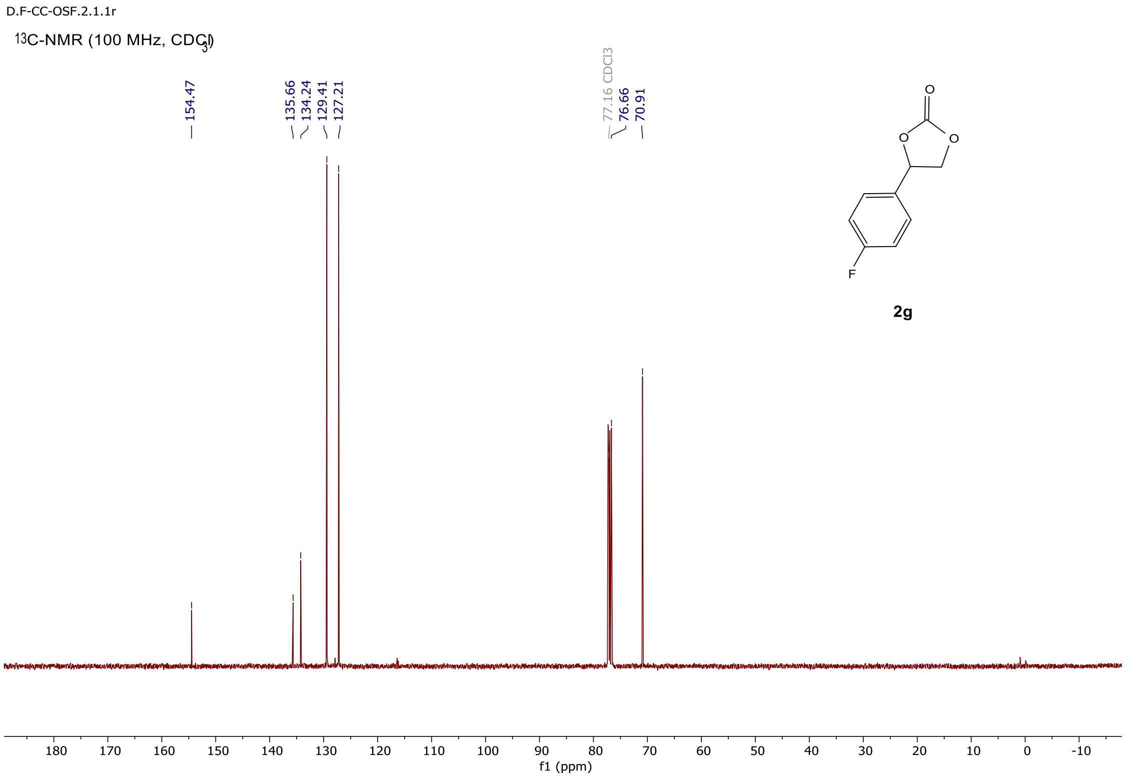

Figure S31. 4-Fluorostyrene carbonate **2g**: ^{13}C NMR spectrum in $CDCl_3$**.**

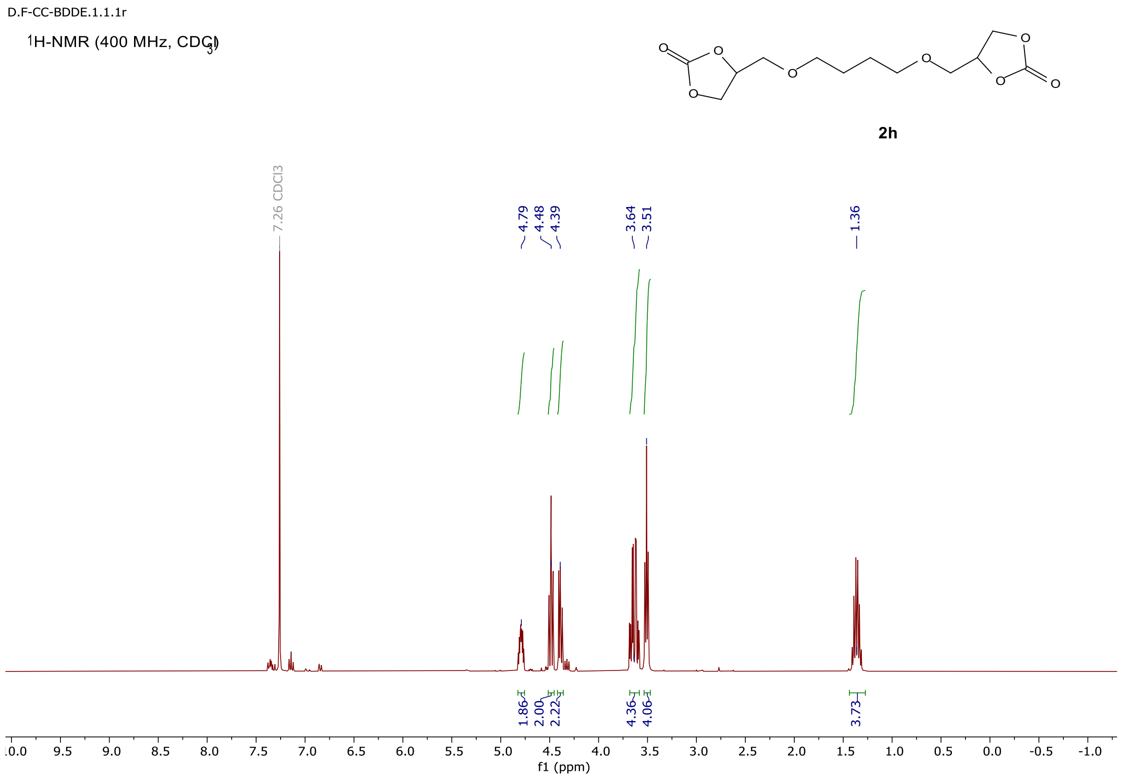

Figure S32. 1,4-butanediol diglycidyl ether carbonate **2h**: ^{1}H NMR spectrum in $CDCl_3$.

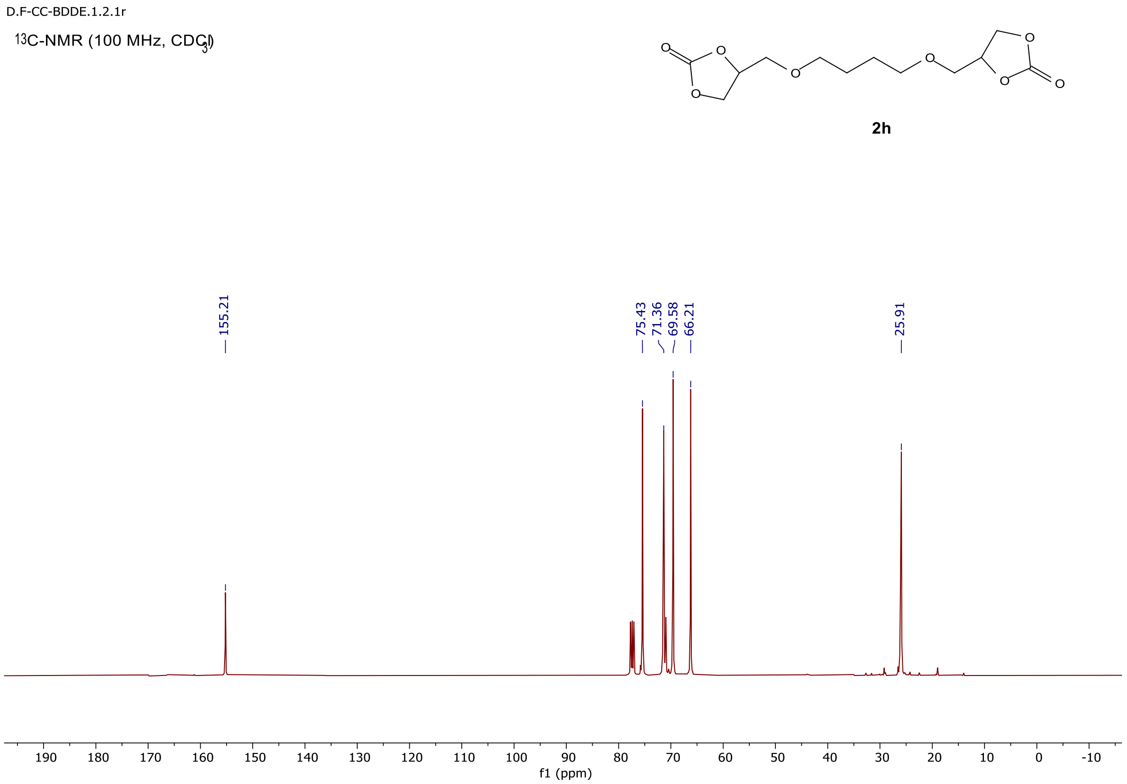

Figure S33. 1,4-butanediol diglycidyl ether carbonate **2h**: ^{13}C NMR spectrum in $CDCl_3$.

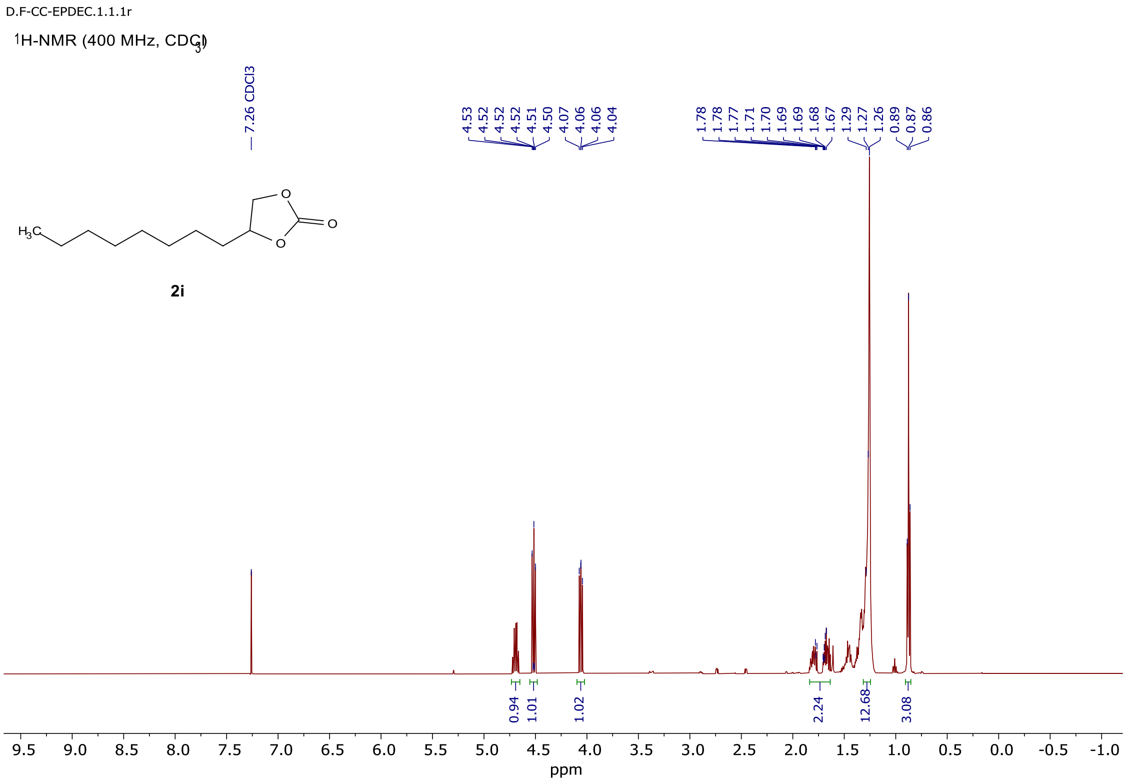

Figure S34. 1,2-Decilene carbonate **2i**: ^{1}H NMR spectrum in $CDCl_3$.

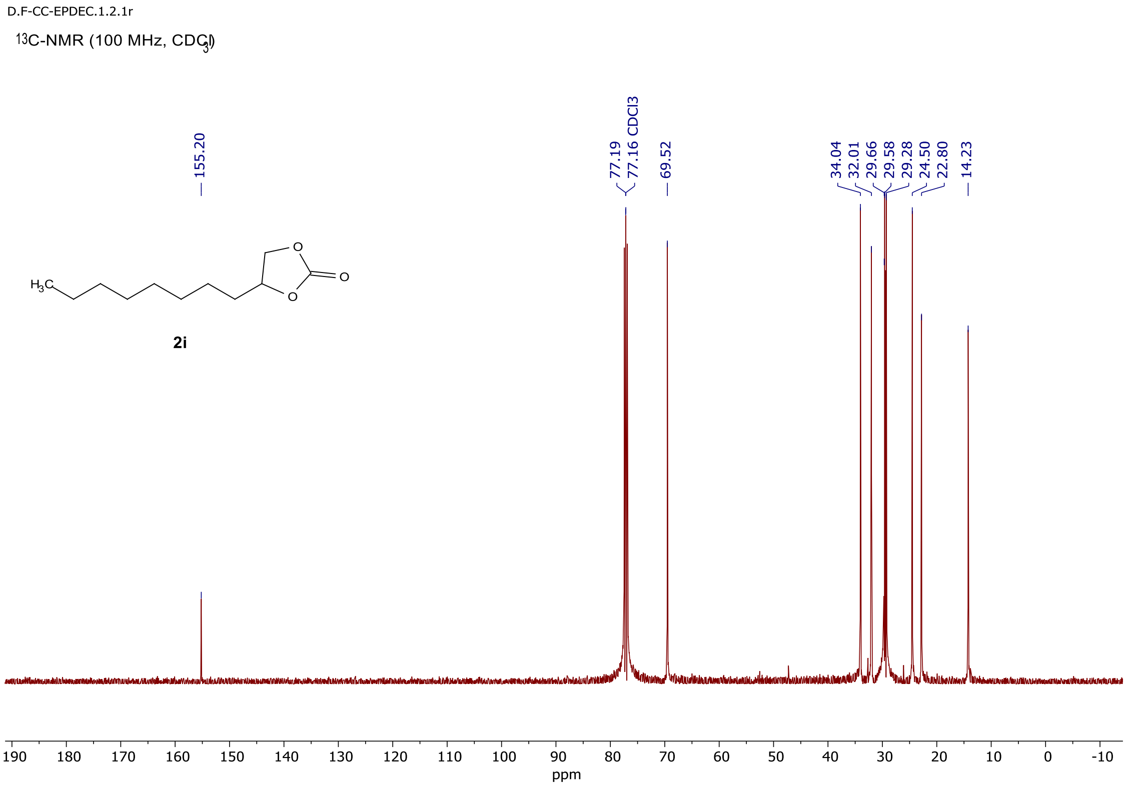

Figure S35. 1,2-Decilene carbonate **2i**: ^{1}H NMR spectrum in $CDCl_3$.

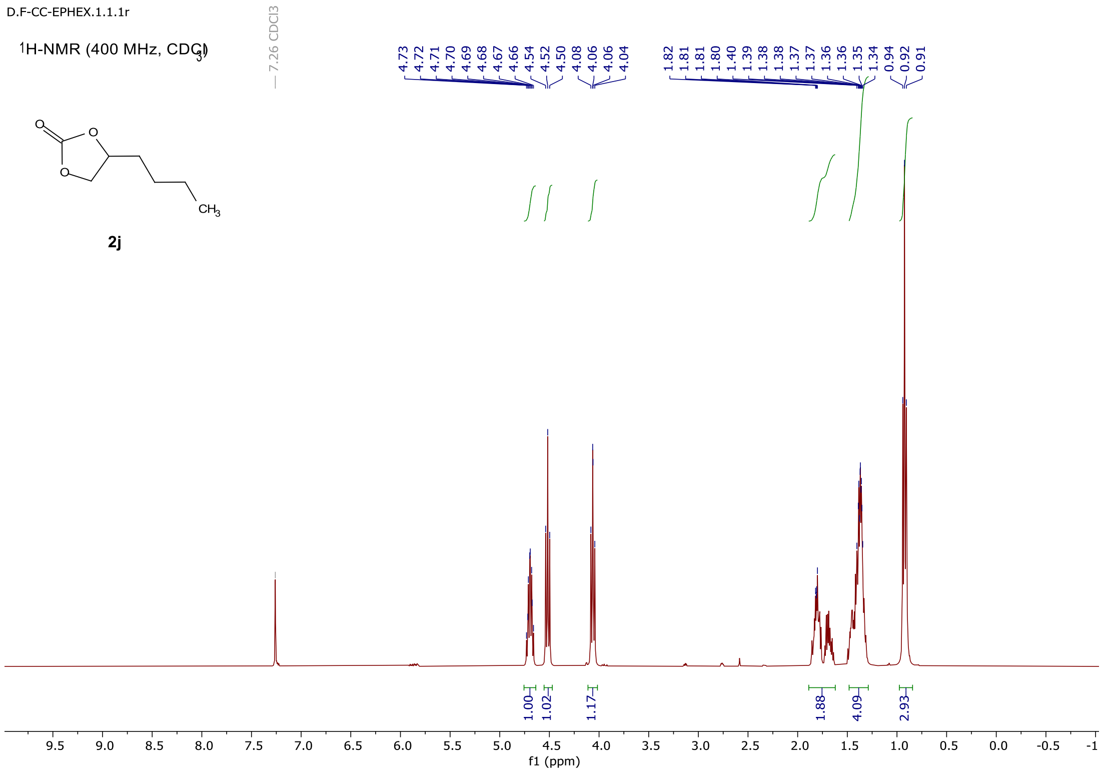

Figure S36. 1,2-Hexylene carbonate **2j**: ^{1}H NMR spectrum in $CDCl_3$.

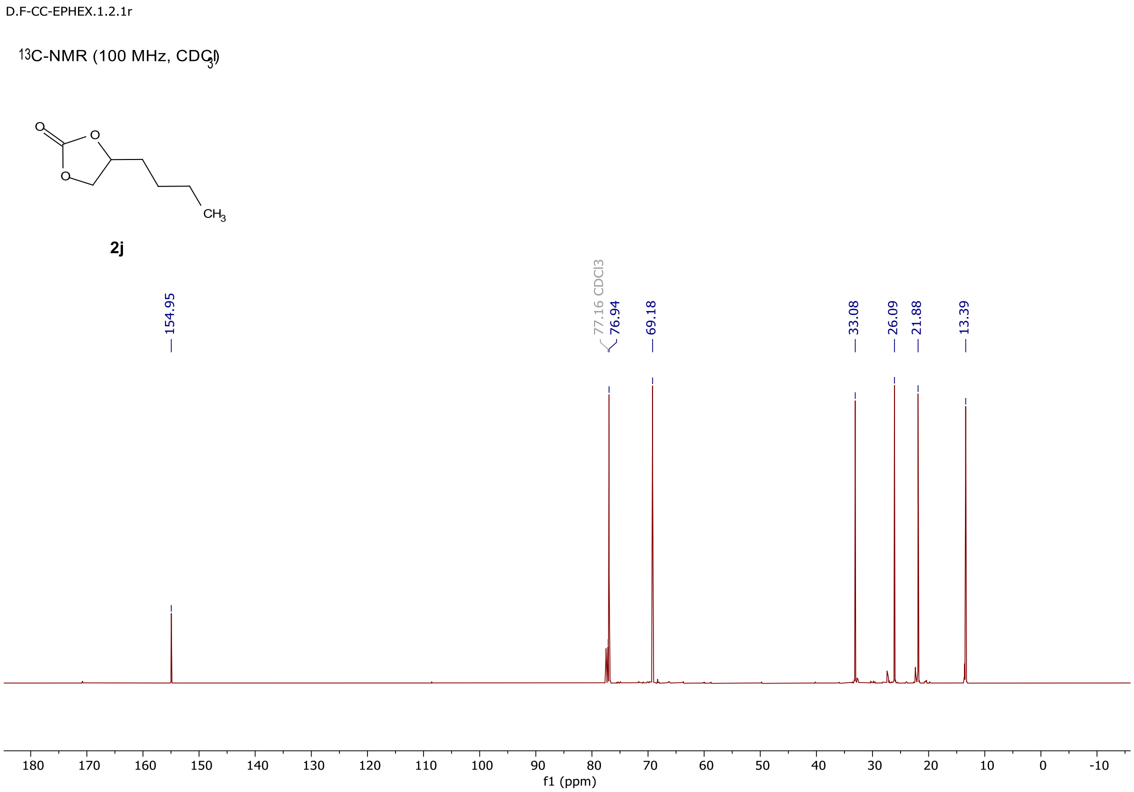

Figure S37. 1,2-Hexylene carbonate **2j**: ^{13}C NMR spectrum in $CDCl_3$.

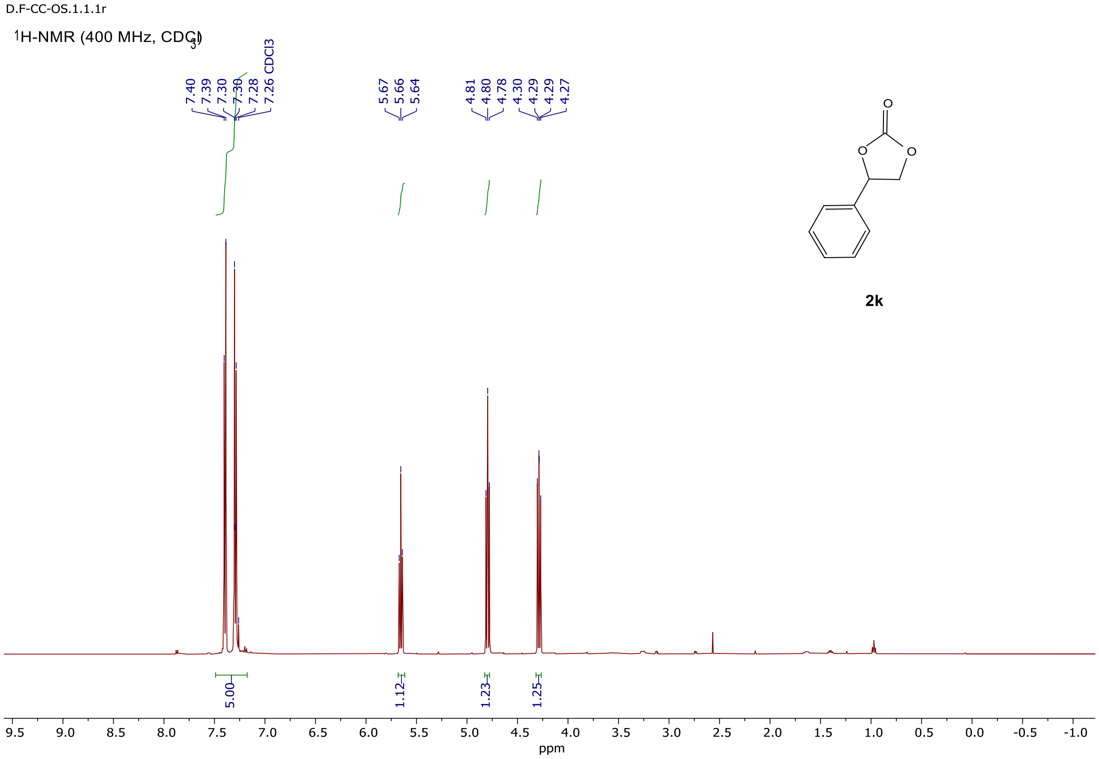

Figure S38. Styrene carbonate **2k**: ^{1}H NMR spectrum in $CDCl_3$.

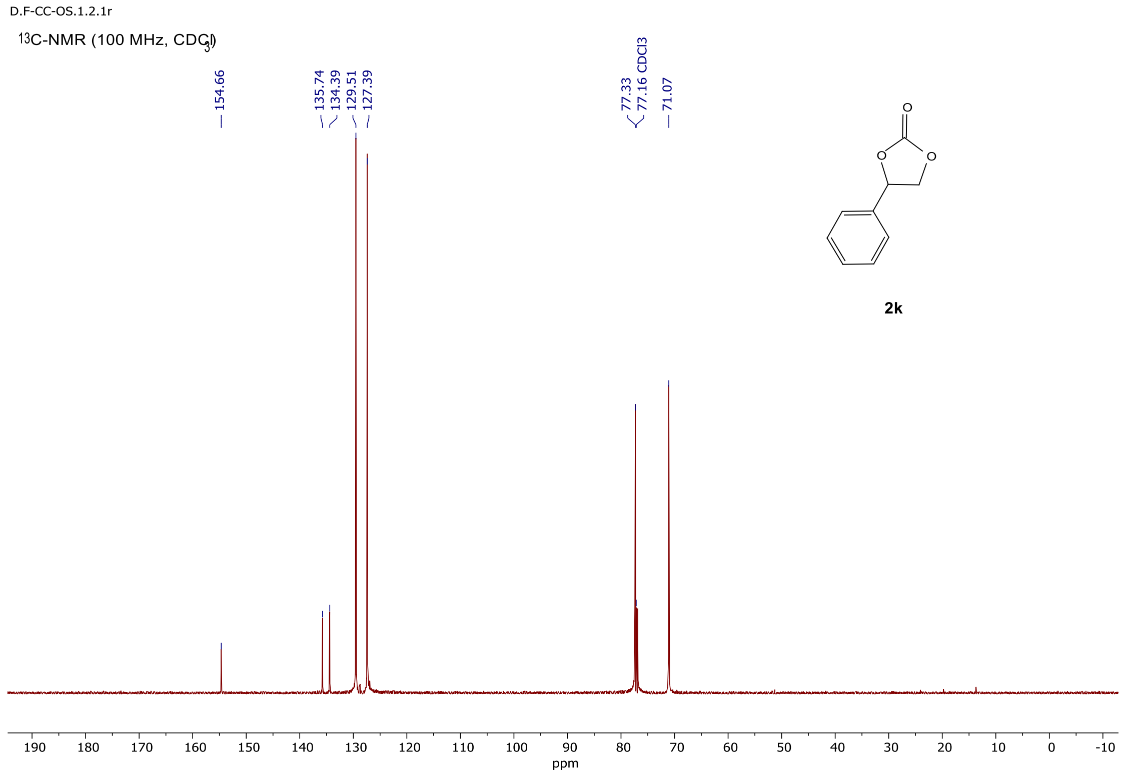

Figure S39. Styrene carbonate **2k**: ^{13}C NMR spectrum in $CDCl_3$.

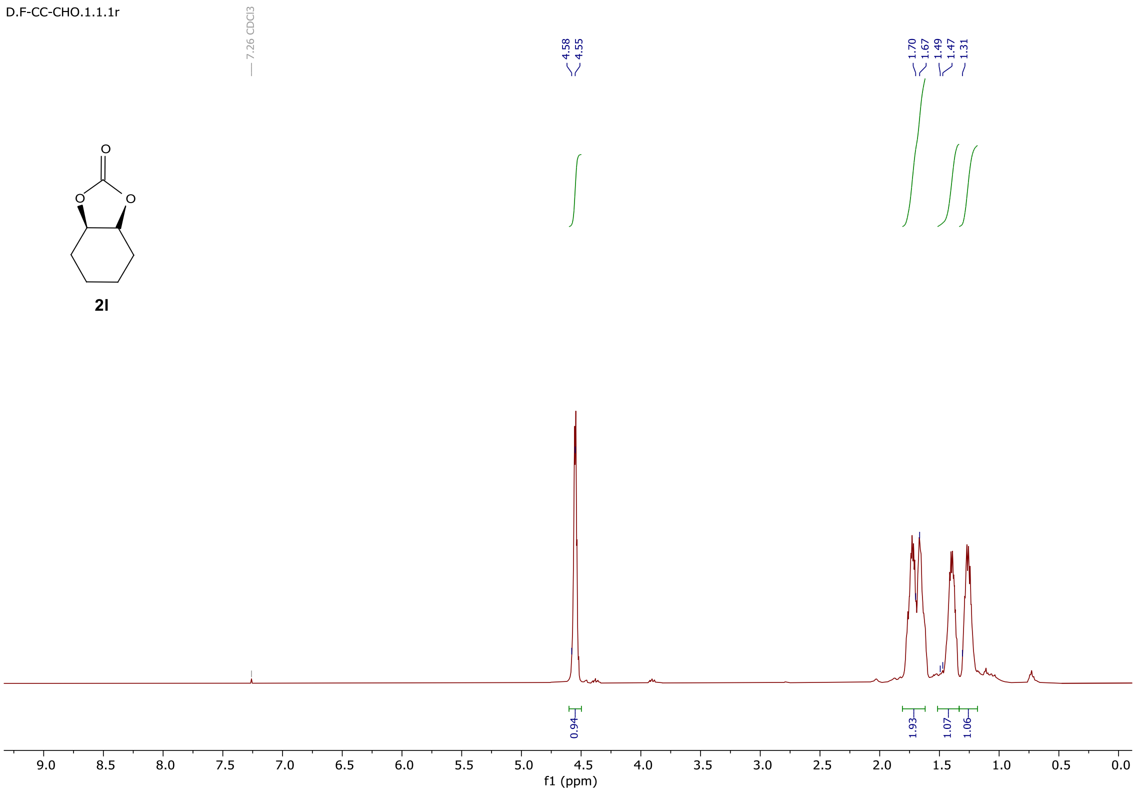

Figure S40. cis-1,2-cyclohexane carbonate **2l**: ^{1}H NMR spectra in $CDCl_3$.

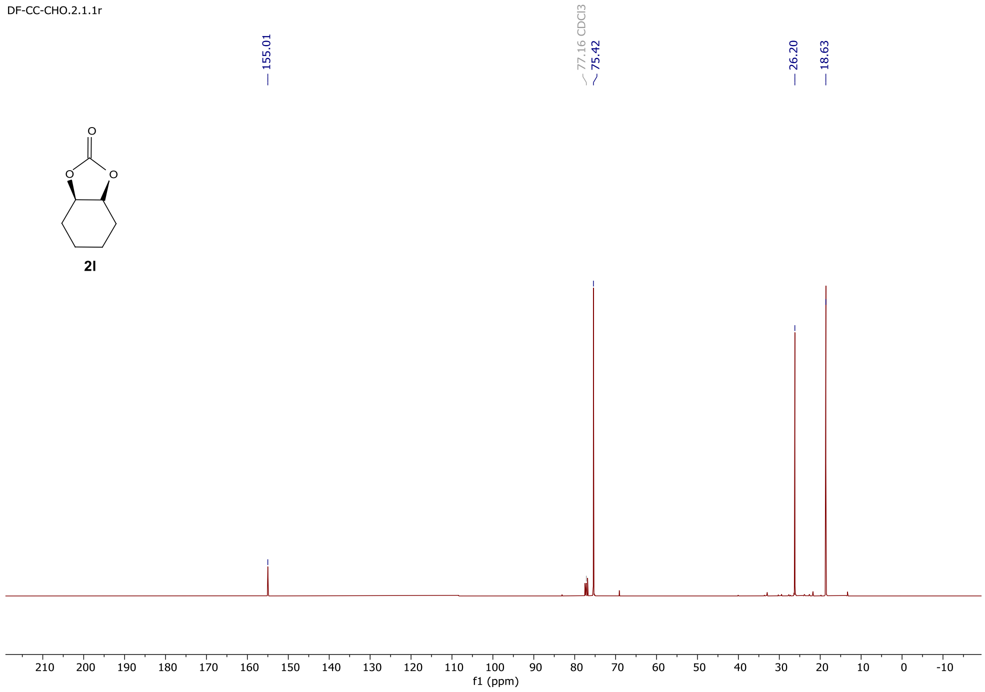

Figure S41. cis-1,2-cyclohexane carbonate **2l**: ^{13}C NMR spectra in $CDCl_3$.

4. Catalyzed Reaction Mechanism Toward Cyclic Carbonate

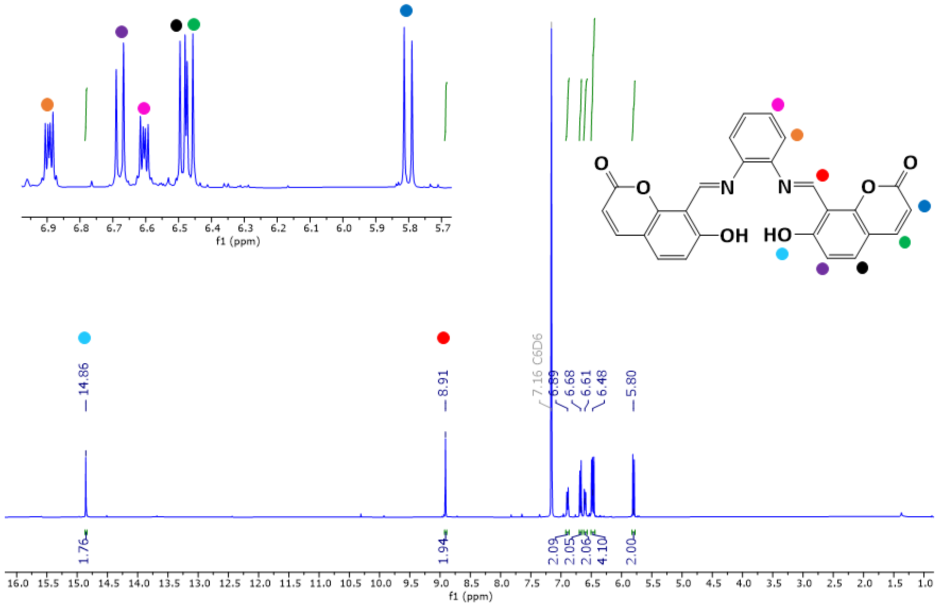

Figure S42. ^{1}H-NMR of **L1** in C_6D_6.

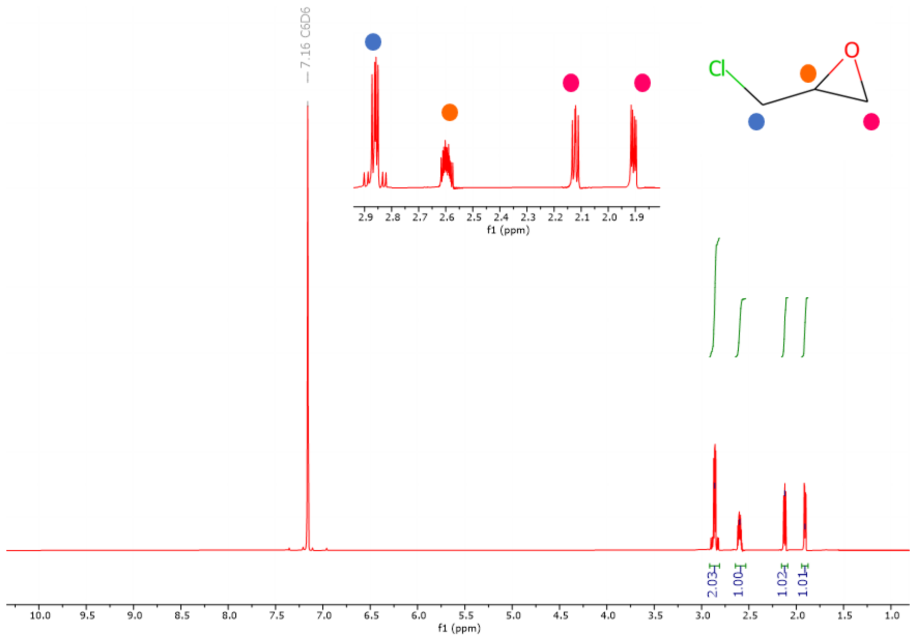

Figure S43. ^{1}H-NMR of **1c** in C_6D_6.

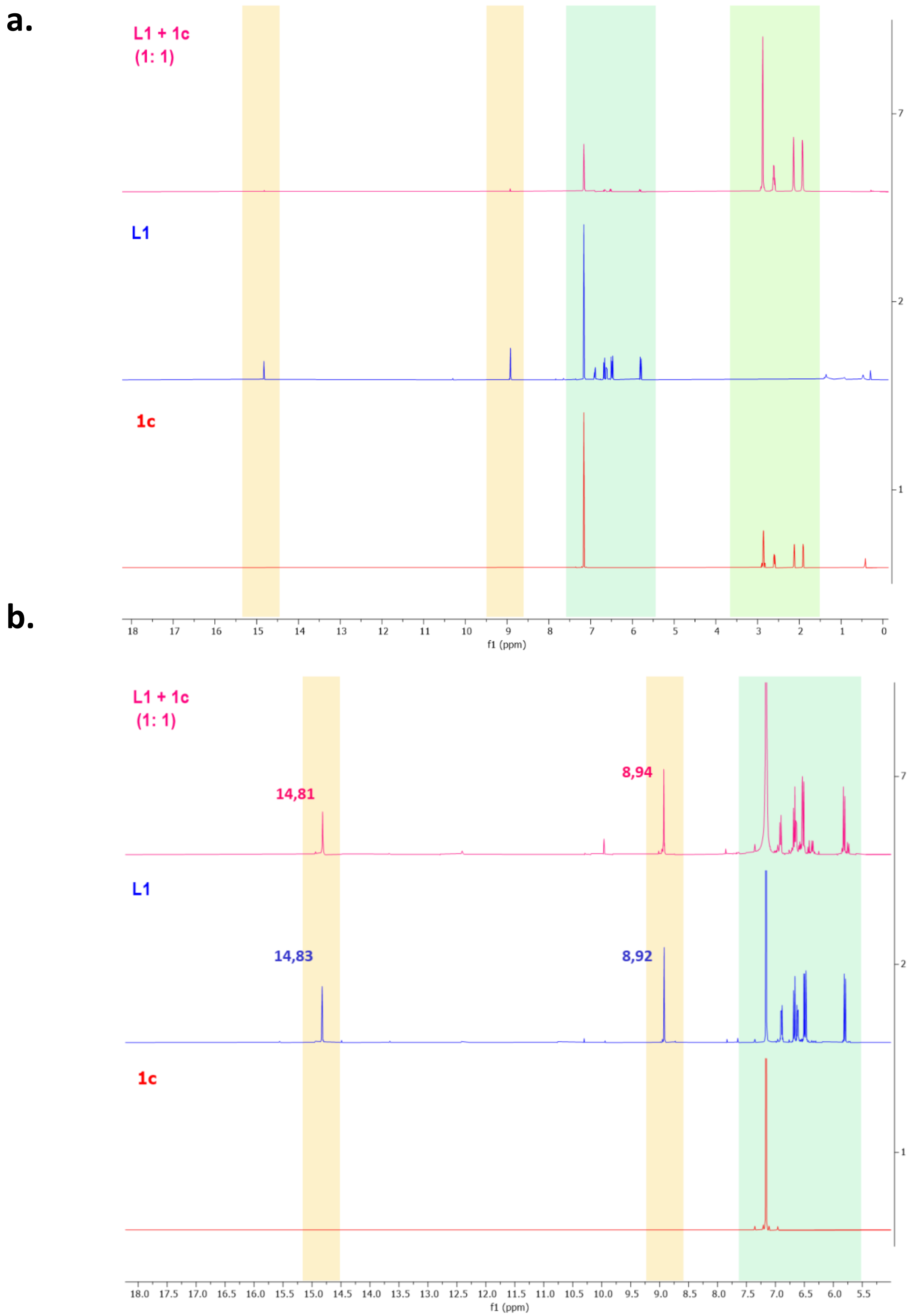

Figure S44. Comparative ^{1}H-NMR of **L1**: **1c (1:1)** in C_6D_6 at 80 °C. (**a**) Phenolic signals and imine signals of compound (**L1**) (orange), aromatic signals of compound (**L1**) (blue), and epoxide (**1c**) signals (green); (**b**) ^{1}H-NMR spectrum of the change of phenolic and imine signals (range 5.5-18 ppm).

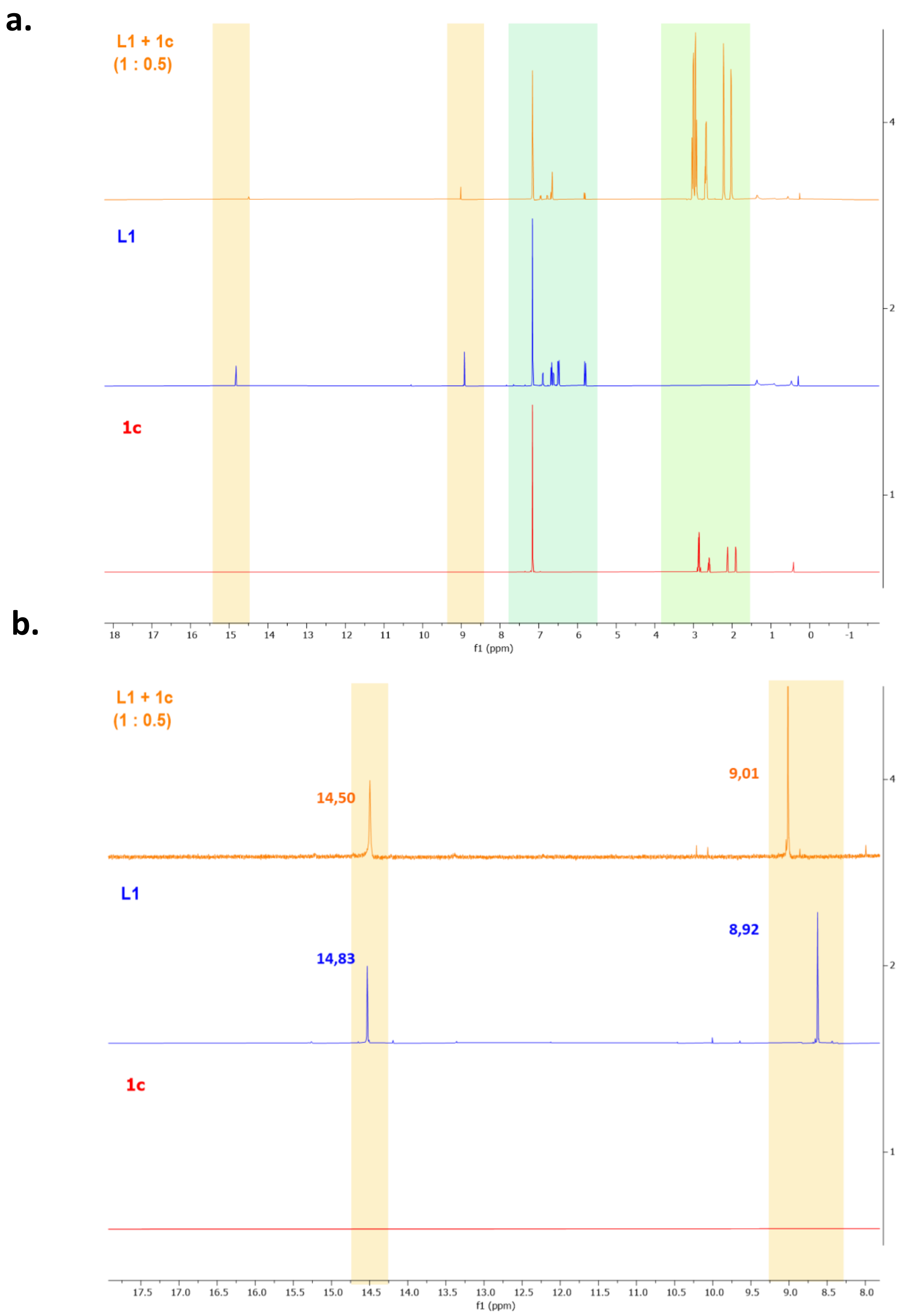

Figure S45. Comparative ^{1}H-NMR of **L1**: **1c (1:0.5)** in C_6D_6 at 80 °C . (**a**) Phenolic signals and imine signals of compound (**L1**) (orange), aromatic signals of compound (**L1**) (blue), and epoxide (**1c**) signals (green); (**b**) ^{1}H-NMR spectrum of the change of phenolic and imine signals (range 5.5-18 ppm).

a.

b.

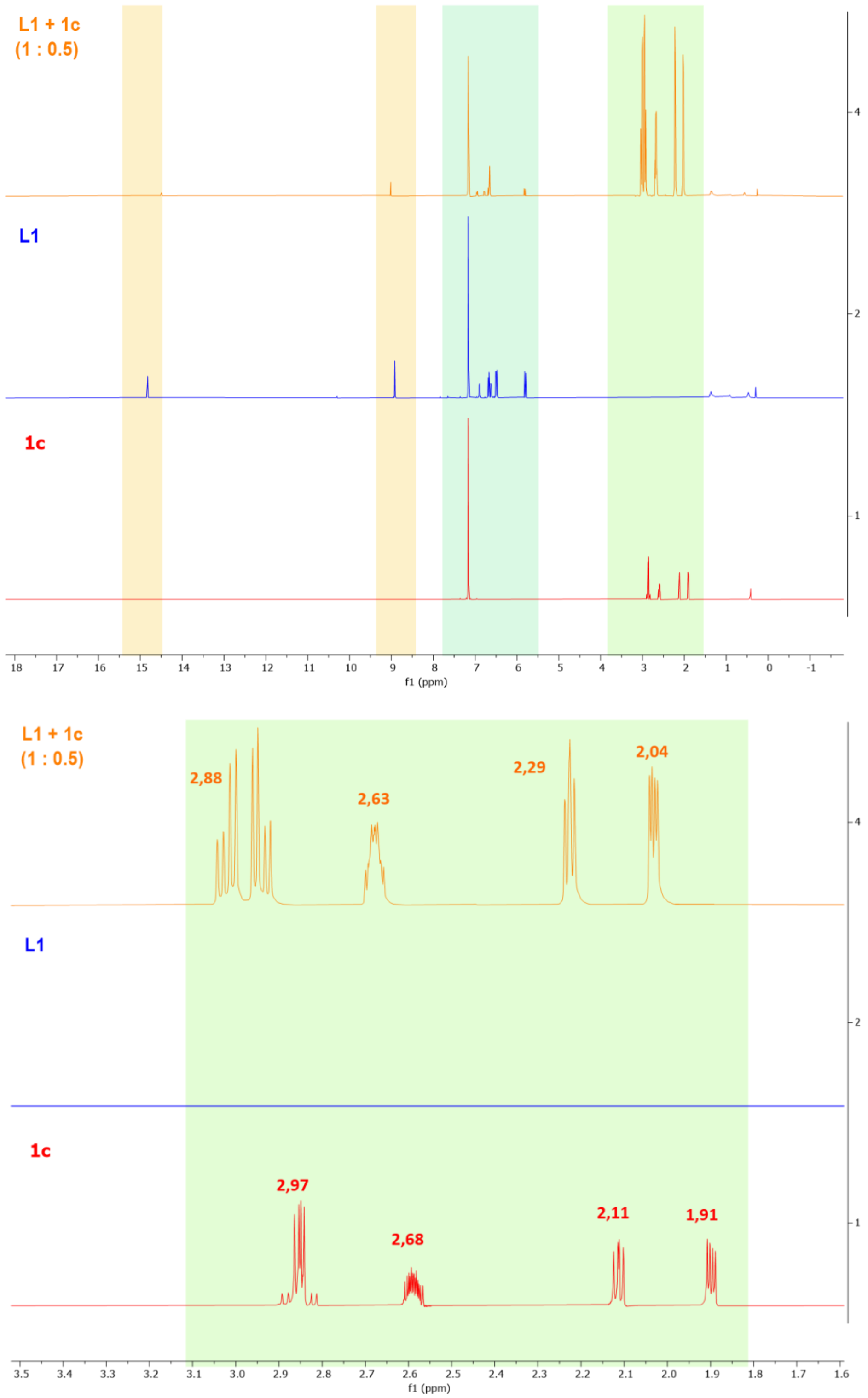

Figure S46. Comparative ^{1}H-NMR of **L1**: **1c (1:0.5)** in C_6D_6 at 80 °C .(**a**) Phenolic signals and imine signals of compound (**L1**) (orange), aromatic signals of compound (**L1**) (blue), and epoxide (**1c**) signals (green); (**b**) ^{1}H-NMR spectrum of the change of epoxide signals (range 1.6-3.5 ppm).

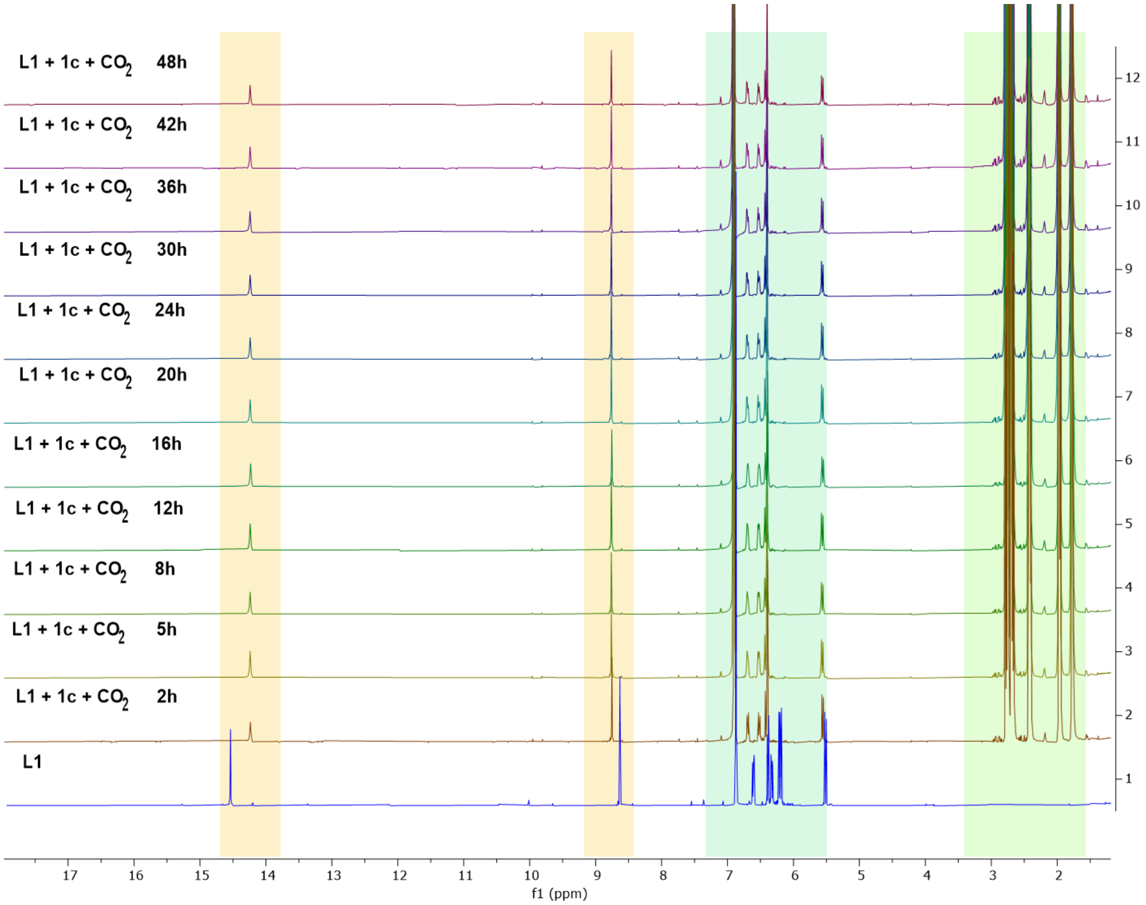

Figure S47. Comparative ^{1}H-NMR of **L1**: **1c (1:0.5)** in C_6D_6 after bubbling CO_2 for up to 48h no change in the spectrum was observed. Phenolic signals and imine signals of compound (**L1**) (orange), aromatic signals of compound (**L1**) (blue), and epoxide (**1c**) signals (green).

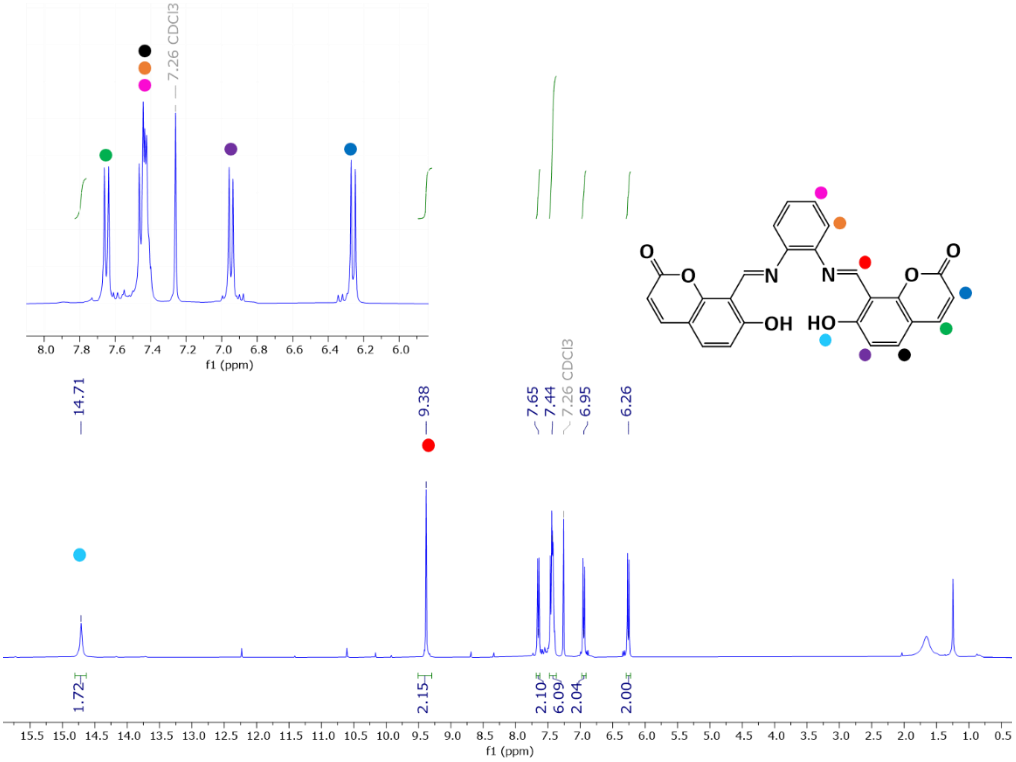

Figure S48. ^{1}H-NMR of **L1** in $CDCl_3$.

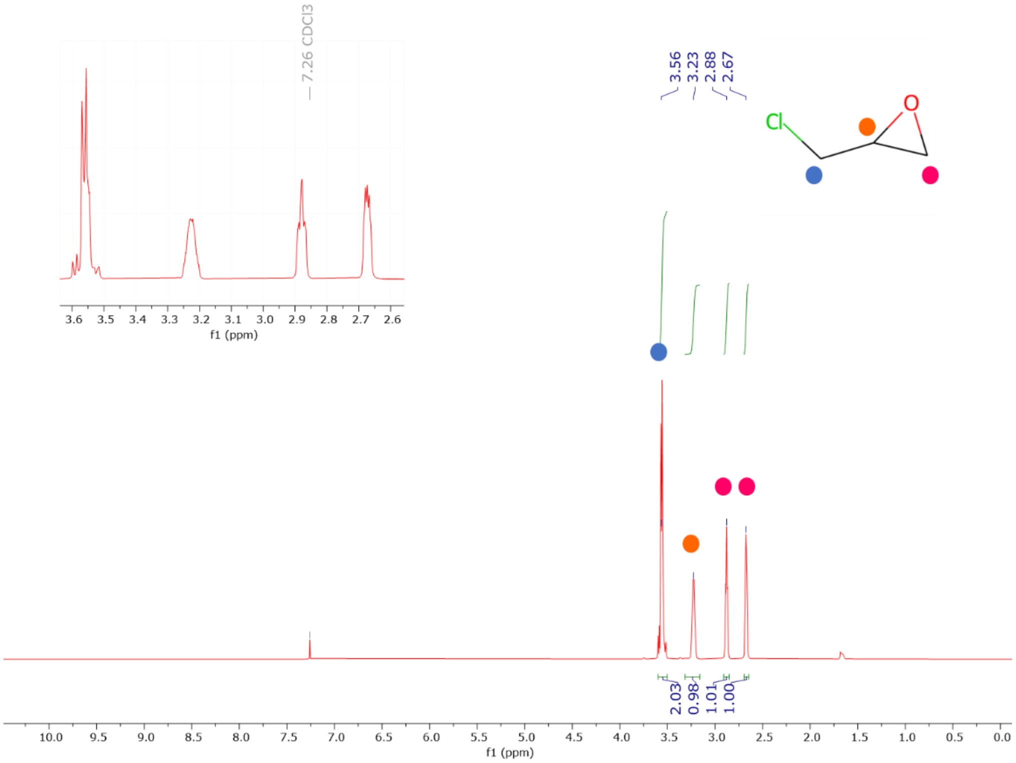

Figure S49. ^{1}H-NMR of **1c** in $CDCl_3$.

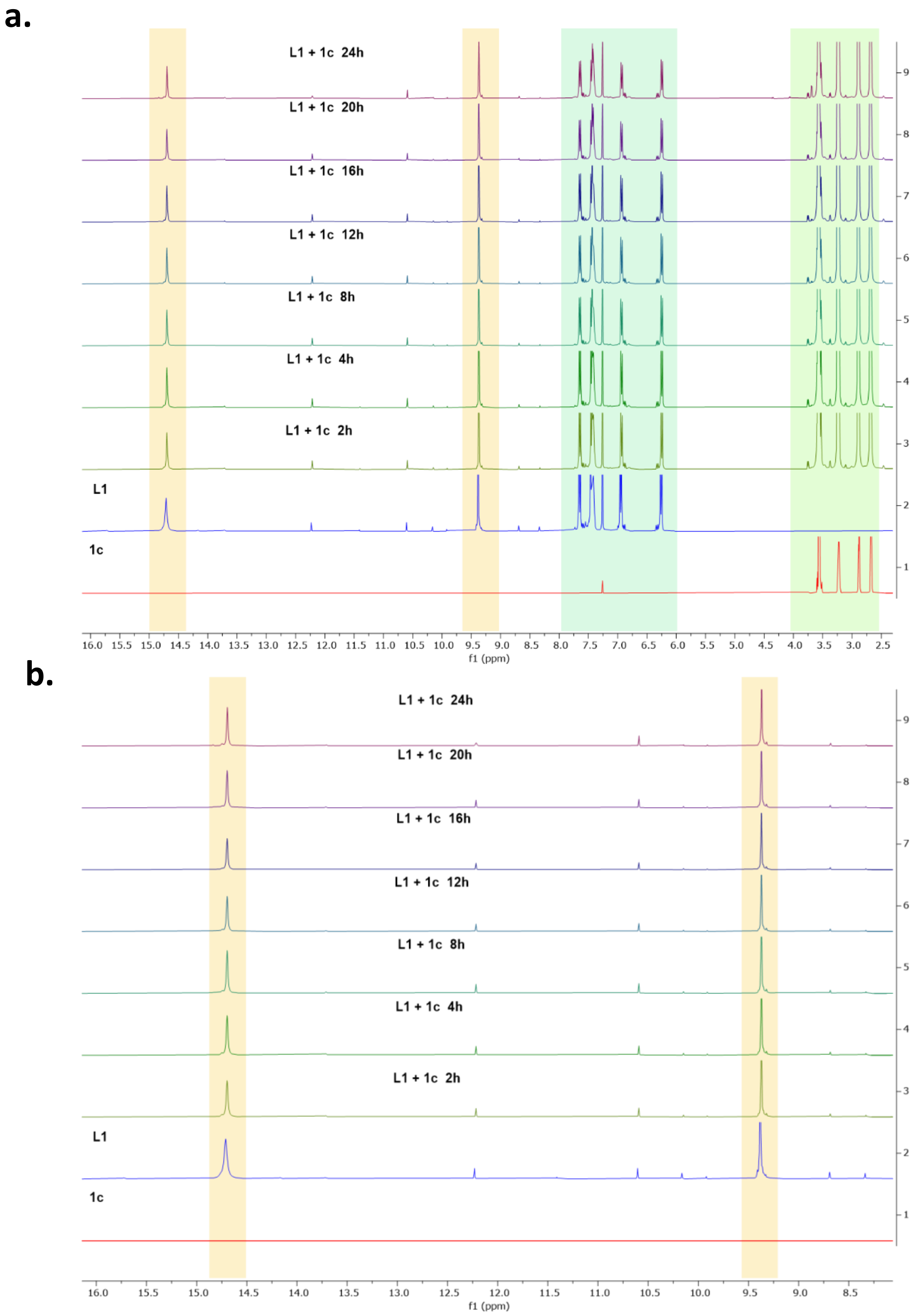

Figure S50. Comparative ^{1}H-NMR of **L1**: **1c (1:1)** in $CDCl_3$ for 24h at r.t. and no changes in the signals were observed. (**a**) Phenolic signals and imine signals of compound (**L1**) (orange), aromatic signals of compound (**L1**) (blue), and epoxide (**1c**) signals (green). (**b**) ^{1}H-NMR spectrum of the change of phenolic and imine signals (range 8.5-16 ppm).

a.

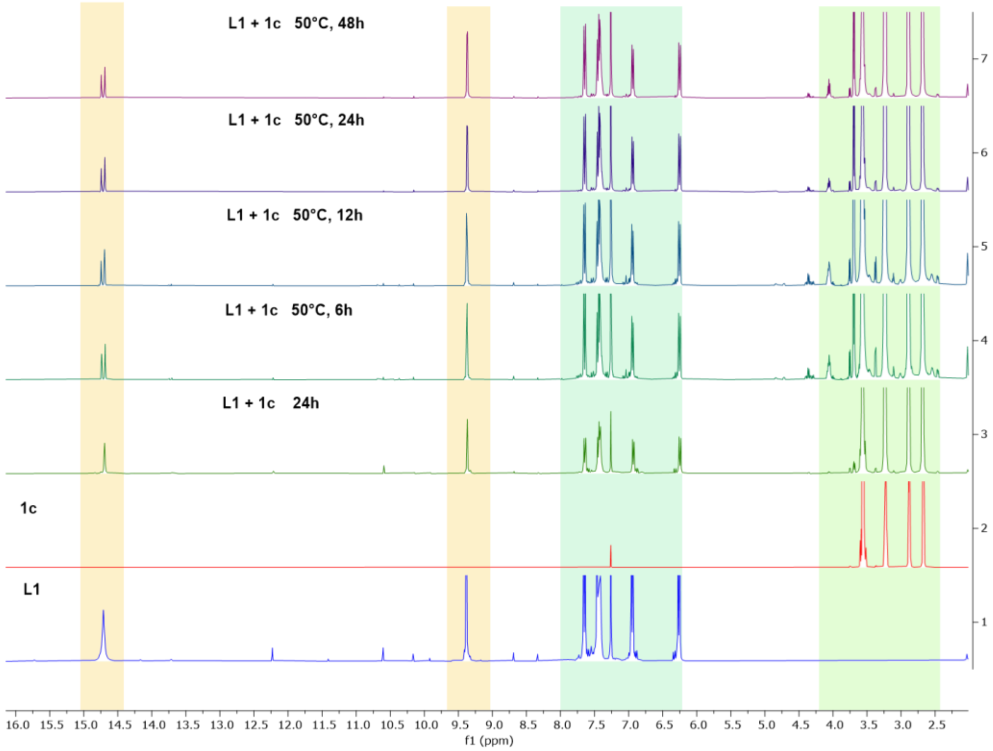

b.

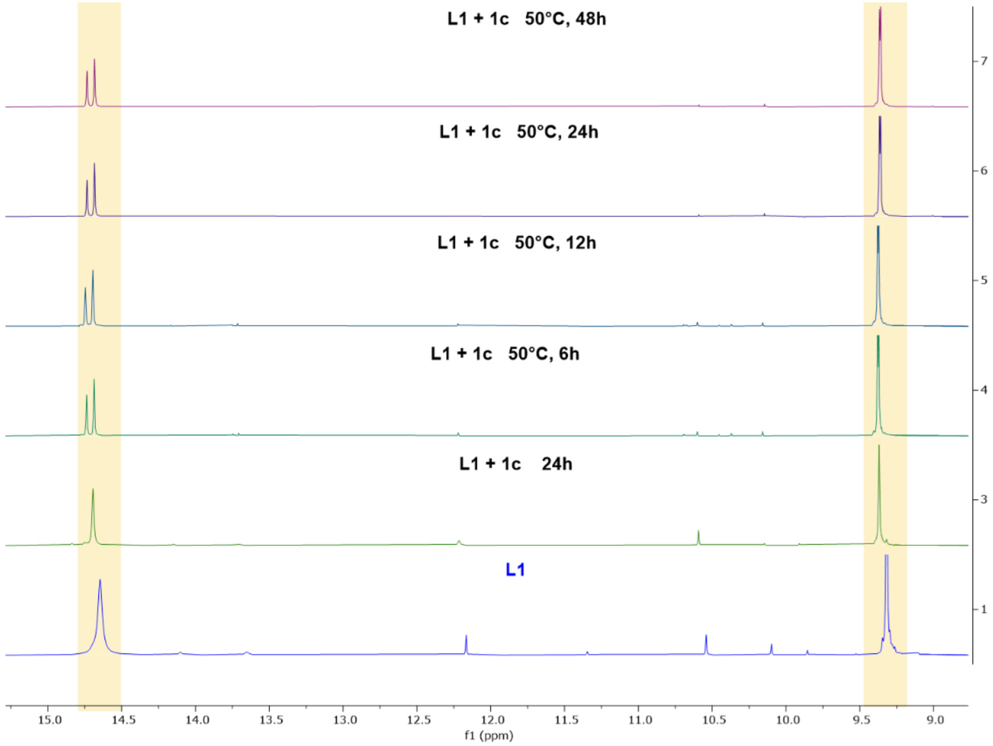

Figure S51. Comparative ^{1}H-NMR of **L1**: **1c (1:1)** in $CDCl_3$ at 50 °C for 48h and a change in the phenolic signals was observed. (**a**) Phenolic signals and imine signals of compound (**L1**) (orange), aromatic signals of compound (**L1**) (blue), and epoxide (**1c**) signals (green). (**b**) ^{1}H-NMR spectrum of the change of phenolic and imine signals (range 8-16 ppm).

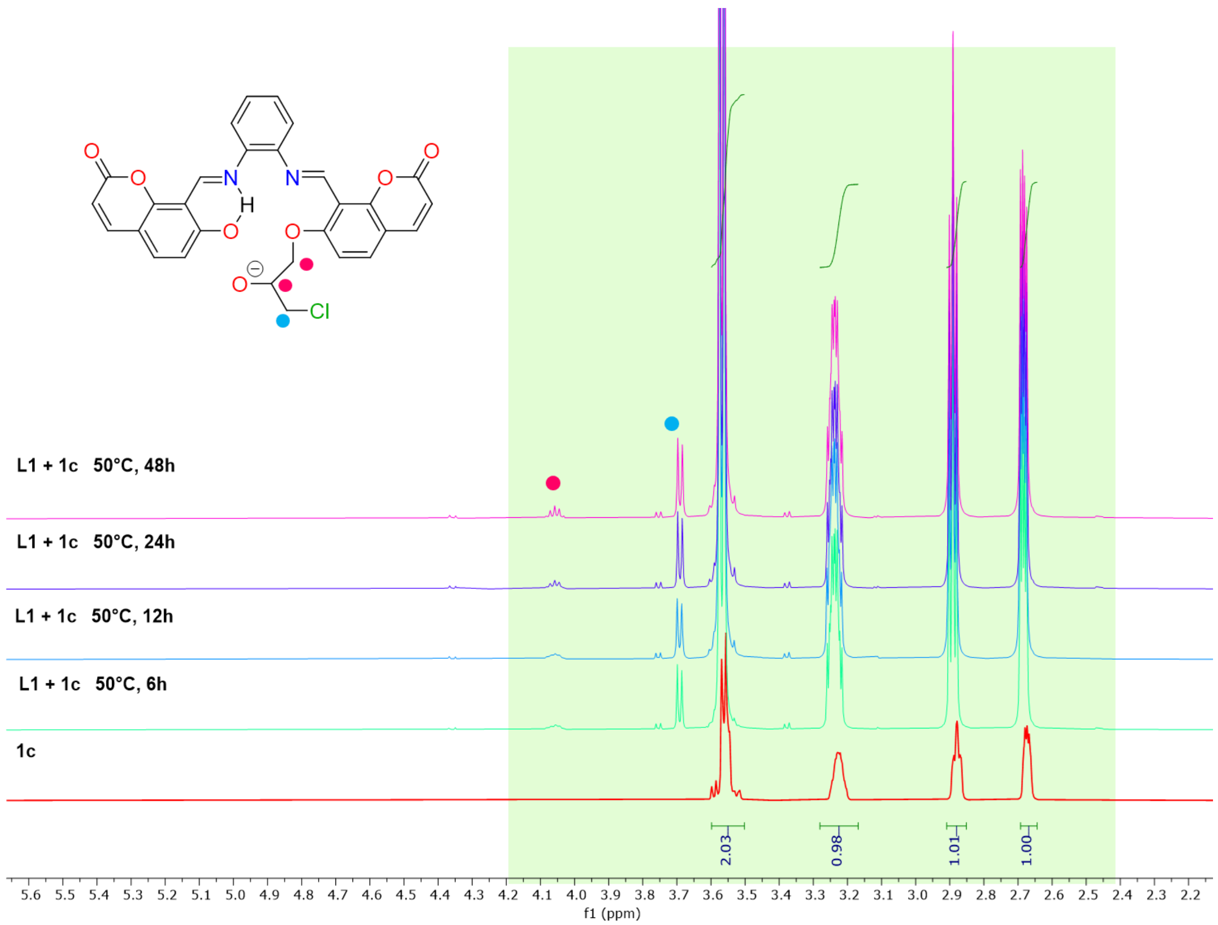

Figure S52. Comparative ^{1}H-NMR of **L1**: **1c (1:1)** in $CDCl_3$ at 50 °C for 48h and the apparition of new signals (doublet and triplet at 3.70 and 4.05 ppm, respectively) in the downfield were observed. Epoxide (**1c**) signals (green).

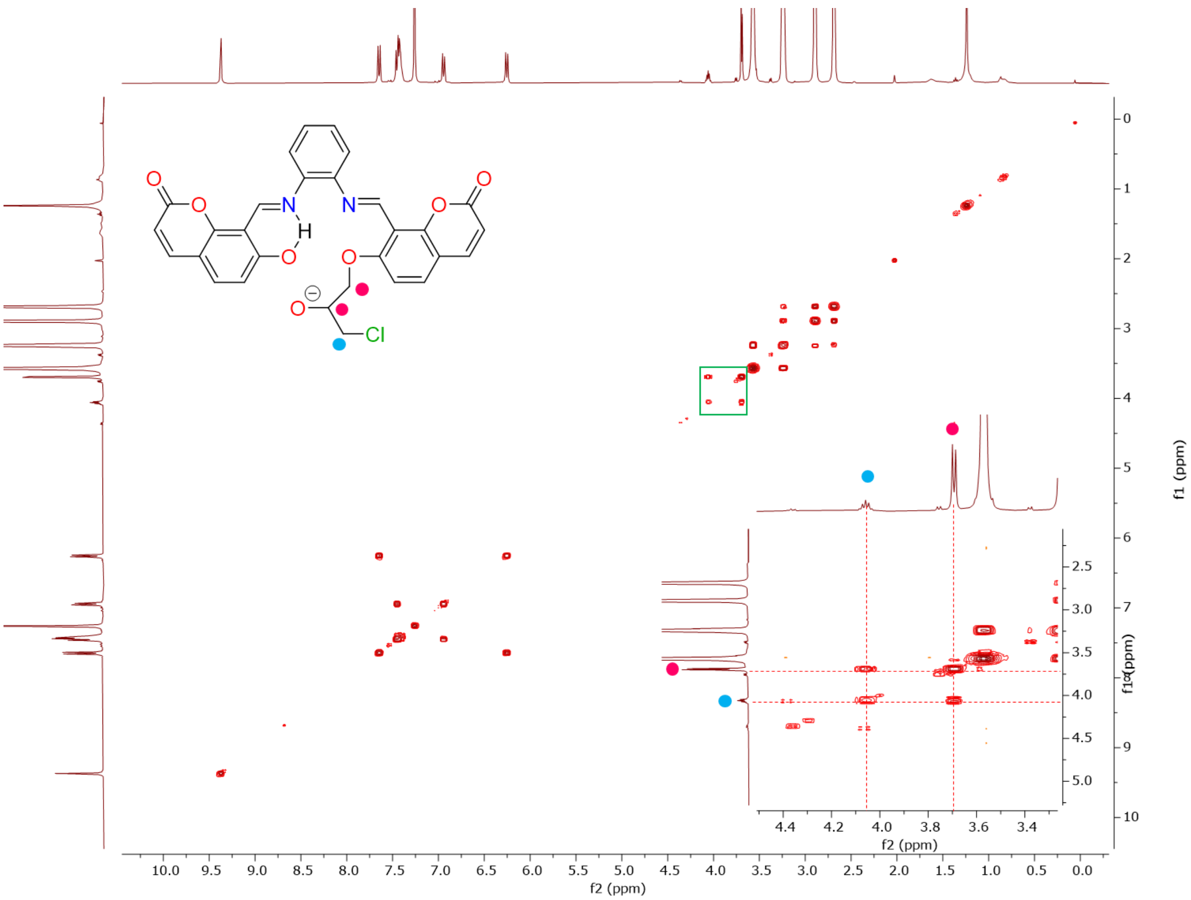

Figure S53. COSY ^{1}H-NMR of **L1**: **1c (1:1)** in $CDCl_3$ at 50 °C for 48h to confirm the coupling of the doublet and triplet at 3.70 and 4.05 ppm, respectively.

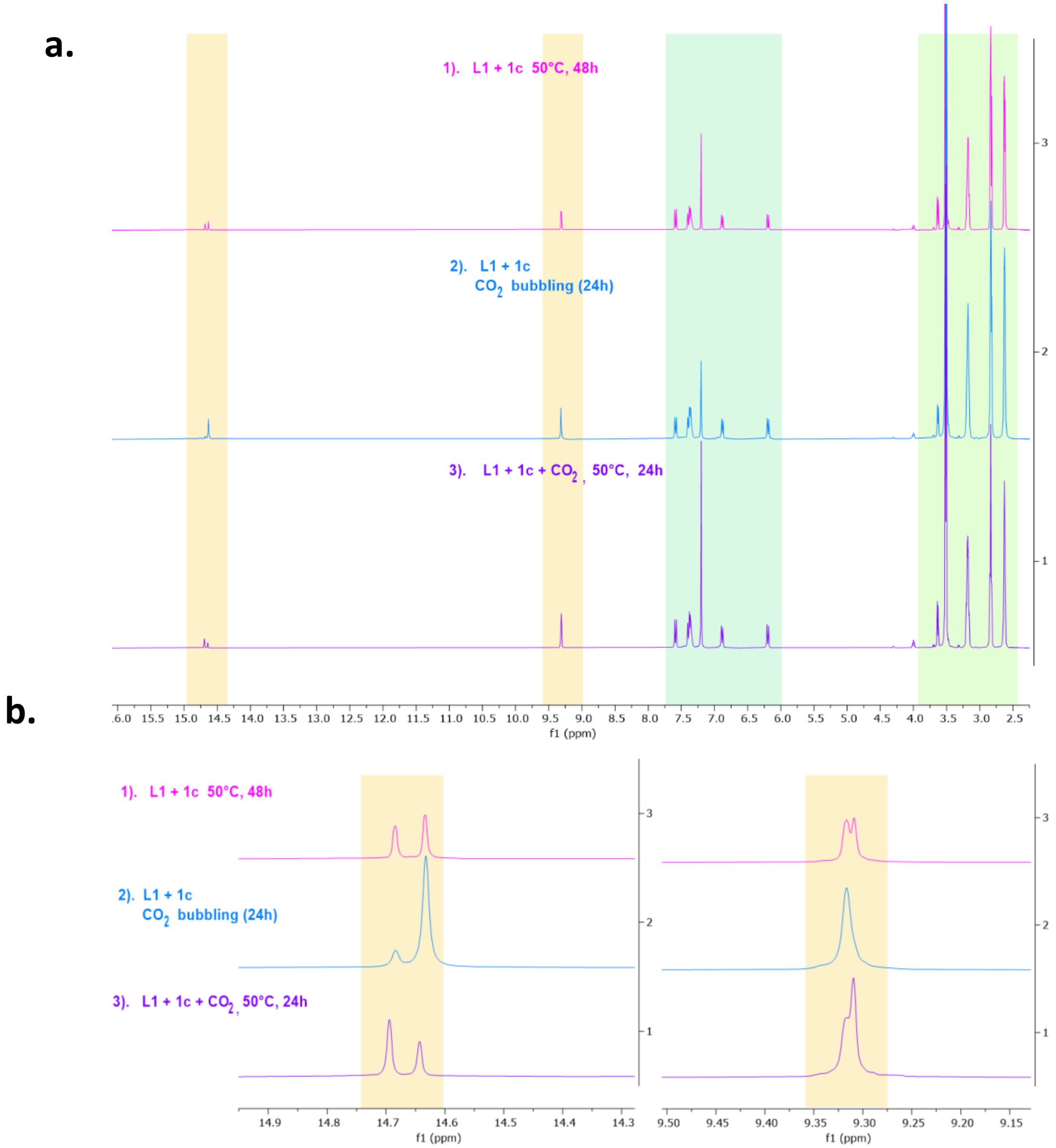

Figure S54. Comparative ^{1}H-NMR of **L1**: **1a (1:1)** in $CDCl_3$ at 50 °C for 48h after bubbling CO_2 for 24 h and the change in the phenolic and imine signals was observed. (**a**) Phenolic signals and imine signals of compound (**L1**) (orange), aromatic signals of compound (**L1**) (blue), and epoxide (**1c**) signals (green). (**b**) ^{1}H-NMR spectrum of the change of phenolic and imine signals (range 9.15-14.9 ppm).

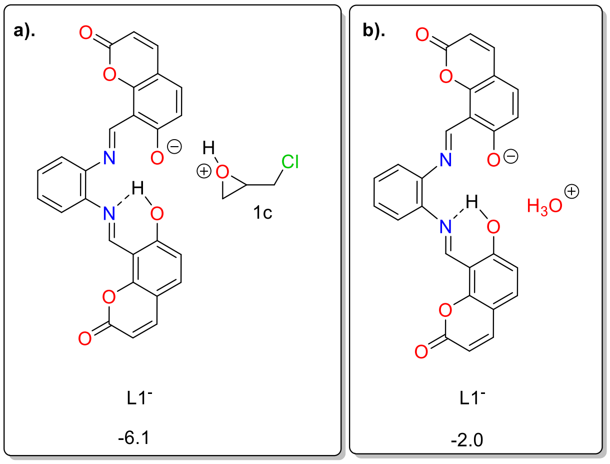

Figure S55. Different schemes for **L1** ionization (kcal·mol^{-1}).

Optimized geometries and negative frequencies of TSs.

L1$^-$

Coordinates from ORCA-job l1.opt

O	13.57517892487903	14.11464691817736	9.20489810791617
C	13.74826788669608	15.10422386099719	10.07718361067563
C	14.16972094882900	16.35614619572563	9.59749034367135
C	14.36746969131978	17.39792063114986	10.46996218427813
H	14.69140182737017	18.36335197035749	10.10160966743593
C	14.15674157225471	17.24027238921746	11.85324742590841
C	14.34246405524096	18.28198388182671	12.81026079378407
H	14.66531309222678	19.25508193483912	12.45911345933635
C	14.12302176320184	18.06861342253998	14.12784810557693
H	14.25643730747714	18.84078006759953	14.87114634391498
C	13.69376541060710	16.78039360310994	14.60644777646938
O	13.46547237677151	16.46548430474651	15.75532697179106
O	13.52177351944639	15.79042179469316	13.64795294802742
C	13.73955110233254	15.99062492598275	12.32049375786509
H	14.33241391225366	16.47234829331310	8.53458431408084
C	13.52320132195720	14.90095400695240	11.46330271759161
C	13.10801806334134	13.61105295125834	11.97256583992250

H	13.02672543269455	13.49413725196415	13.05207559604062
N	12.87328356829617	12.64305284013568	11.16073214599724
C	12.43969108161123	11.38273302729650	11.57898965440911
C	11.67685876328573	11.18153137929784	12.73275390468734
H	11.38648035888922	12.03467837539257	13.33220929162911
C	11.26345260162653	9.90814559779407	13.09761232600282
H	10.65945267025057	9.77047078095292	13.98515813847048
C	11.60850858528867	8.81649312788622	12.30333285481193
H	11.27814497881183	7.82133528222578	12.57390332278375
C	12.35635110240183	9.00239819864377	11.14892737098159
H	12.59368744424137	8.15935800349324	10.51271586696708
C	12.78236623807789	10.28018364300897	10.76039700875235
O	13.82901205530062	11.24272889159871	6.80433158035466
C	14.85480499825487	10.52437037546131	6.86839006145244
C	15.73504102695613	10.41329777968978	5.71926425591730
H	15.45618622925751	10.98858540796520	4.84476526882294
C	16.84665079756212	9.63356783003070	5.71796348388552
H	17.47993218045177	9.57418931132374	4.83961134617836
C	17.21321220166971	8.87447916669500	6.86062809619819
C	18.35408162093221	8.04561691531300	6.92169989512771
H	18.99030814755126	7.98063615821754	6.04576981962959
C	18.66759942471159	7.33458110174893	8.04244958017888
H	19.53674268247390	6.69643621244265	8.10132541641324
C	17.83775762837867	7.41684256553027	9.20098349038856
O	17.98778772933329	6.84542495551337	10.26895544816882
O	16.72722982015117	8.24744025235468	9.10806052987168
C	16.38945232512231	8.96205266990428	7.99233221170302
C	15.23044597291610	9.75587297193837	8.05433213973282
C	14.46703975927480	9.74121492156133	9.27831832156266
H	14.80087981612697	9.00392728031015	10.01202559102561

N	13.47861690623188	10.50949055554936	9.57826206783420
H	13.26778680966141	13.29996475437268	9.73344490687396

1a

Coordinates from ORCA-job epy.opt

O	16.76993980981679	5.44588233980285	10.39149484305392
C	16.55576295637757	5.89540229528235	9.04512225370594
H	15.54369271673696	6.22931988871093	8.83643555293954
C	16.78841276799685	4.48044075679534	9.31987083652794
H	15.95161797282760	3.79087974695014	9.31354184612817
H	17.76393986389951	4.05499173663998	9.10773897761325
C	17.65016921566052	6.76247830475148	8.50118796136710
Cl	17.55007467752342	6.79989097981108	6.68840497067121
H	17.55477327432381	7.79360751481711	8.83161514656230
H	18.63112674483689	6.37102643643873	8.75646761143060

TS1

Freq: -451.41 cm^{-1}

Coordinates from ORCA-job ts1.scants

O	12.35550426704145	13.95056119887563	8.99805347750873
C	12.67585610388737	14.95303829472103	9.68982570504913
C	12.72850712035163	16.27638481850346	9.12183537077153
C	13.10391098312493	17.35244970442511	9.86136529255726
H	13.14887390835134	18.33618964542390	9.40854589537735
C	13.45406626424307	17.23797344063502	11.23805699776079
C	13.85518630595956	18.33265552103947	12.04859143111453
H	13.90162467491006	19.31873148010892	11.60103924890508
C	14.17709528736941	18.16317434195243	13.35698208253037
H	14.48489085012488	18.98303790641080	13.98890724423629
C	14.12017517352594	16.86539072077270	13.96251795855551

O	14.37354665352619	16.56773989751203	15.11223342687449
O	13.72601924346071	15.81681017279175	13.12942311382854
C	13.40237195438568	15.97940948130621	11.82396071820687
H	12.47299903106653	16.37490980492342	8.07515391536418
C	13.02492391198351	14.82282493796411	11.10002647437770
C	12.97992125759942	13.57728800856751	11.74856253423314
H	13.17316721510480	13.52241150267882	12.81189829956655
N	12.72117008947944	12.45132660343375	11.12934500386361
C	12.54958624248697	11.19058456590490	11.73234967580560
C	11.99394002775697	11.06683524613482	13.00244135281608
H	11.67739756548972	11.95193970392609	13.53834776309395
C	11.81180603365420	9.80918462736977	13.56079182282450
H	11.36663024924181	9.71675667915543	14.54248002889655
C	12.16544772178561	8.67356660055959	12.83628752125622
H	11.99672967050552	7.68929142951483	13.25434590769479
C	12.72038951872224	8.79485559072351	11.56990640618644
H	12.97597442288273	7.90931035813354	11.00309903768250
C	12.94504115293494	10.05390961088396	10.99970136617132
O	14.30066779695464	11.57786083404836	7.35450854376430
C	15.13705983262474	10.62637771470081	7.19308563142171
C	16.02885171888666	10.60149590889053	6.06992730247711
H	15.97262499022500	11.41403310718129	5.36010474398886
C	16.91640930284061	9.58562557874703	5.87516057098890
H	17.56983535233846	9.58761428388081	5.01043351182319
C	17.00582926710425	8.50609918001502	6.78306556512622
C	17.90288217320820	7.41886297925879	6.63277698156689
H	18.56052295387631	7.40883314784130	5.77084318555209
C	17.95216630428214	6.40738879766582	7.53870993623145
H	18.62920560106369	5.57171722910611	7.44125085737019
C	17.08717116226395	6.41419419409294	8.68043171901451

O	17.02533764506090	5.58175780338793	9.56679656925825
O	16.21554743140320	7.48966646779945	8.78962230336707
C	16.15587526298297	8.51909550008871	7.89491823674577
C	15.22432910507233	9.54391734854851	8.14569316941551
C	14.48660304620422	9.49440623593299	9.38690624416055
H	14.84925468318286	8.73989561620597	10.08703541549502
N	13.51204386768039	10.25633152862817	9.74015478407165
C	14.73562238331193	13.43078175334663	6.75482751965006
H	14.68566494989686	13.19252862591030	5.70727263700127
C	16.01446847379402	13.93770873024050	7.26432766260590
H	13.84110646015518	13.75401689774409	7.26020232860209
C	16.15427195039931	14.15553311038208	8.75908261226405
O	15.66567922943649	15.11795142625047	6.62409368514923
H	16.90510678181947	13.42976930848281	6.86982191792172
Cl	16.67177542787317	12.65526229944262	9.65959144298665
H	15.21901545332114	14.47317872622414	9.20824634711595
H	16.92793976954228	14.89604459027203	8.94670966322661
H	12.61688872223143	12.53382918132571	10.10900383852287

Int1

Coordinates from ORCA-job int1.opt

O	11.69076745846749	13.78625816371111	9.07199509768844
C	12.26318243720120	14.81859484720291	9.69136117773071
C	12.28099730035271	16.06243195454026	9.04023181749573
C	12.89202025500314	17.13968054565432	9.63375601534604
H	12.91889443478629	18.09687953374894	9.12855435617593
C	13.50509499119723	17.02556479489965	10.89539311389794
C	14.17324625846493	18.10036086020481	11.55549940074554
H	14.20870937560094	19.06598975154116	11.06512791517334
C	14.75469812310212	17.92430217334072	12.76332676196524

H	15.26847318998198	18.72045990721566	13.28166574690469
C	14.71554682568462	16.64526444502593	13.42403150466006
O	15.19541597555986	16.36338771994587	14.50120520666169
O	14.05585441066512	15.62320630932024	12.75393886511152
C	13.47071866960700	15.78502054964625	11.53848541046381
H	11.82047436390803	16.14148957937294	8.06511997855394
C	12.85790203419831	14.66107982009029	10.96617664090408
C	12.87161756457762	13.37680022047176	11.63722248515029
H	13.33020493898331	13.32264802268161	12.62322835940688
N	12.38275128873771	12.33725167780047	11.06992307069353
C	12.35064367403030	11.09492962852538	11.71861249789325
C	11.75521904703495	10.93682097659416	12.96990370733389
H	11.32759180559351	11.80260734832365	13.45963061557830
C	11.68000066334986	9.68110567798544	13.56133336308781
H	11.19865436069087	9.56862368778949	14.52426452194271
C	12.20518883150685	8.57278745569716	12.90423396143714
H	12.13280161191099	7.58936436240811	13.35096779958679
C	12.81885138155632	8.72348801180586	11.66653376165732
H	13.22040035172500	7.86149963464402	11.14930141046566
C	12.90783602635286	9.97998888767767	11.06100800203738
O	14.62817079309572	11.92966222968856	7.89545768842053
C	15.38690862621452	10.90757200114944	7.50577399871352
C	16.19609883068154	10.95094821347536	6.35236492037332
H	16.21436518920282	11.83888237913733	5.74122020912910
C	16.97608999170760	9.87508793935887	6.00289554408910
H	17.58926264644084	9.91906302525037	5.11143719872949
C	17.00228412444713	8.71212465218981	6.78690943143022
C	17.79113043674551	7.56265388197460	6.48745291551317
H	18.41132656128596	7.57894406180759	5.59884408391694
C	17.77273854855888	6.47365533191770	7.28990004554995

H	18.36165684841085	5.59149043761504	7.08621006176880
C	16.95109475964489	6.44295905815353	8.47168598237527
O	16.85317923991155	5.53331666501705	9.26947344433161
O	16.18546080382517	7.57084893966833	8.72414025846848
C	16.20067188894104	8.68213253421661	7.93351828389733
C	15.37496451847477	9.74615734735566	8.31870774353035
C	14.63031806749993	9.62175134191344	9.57251619412796
H	15.10772650249180	8.98914866279803	10.32503344423743
N	13.51661012250091	10.19199269589975	9.81638140523027
C	14.76961057745189	13.23417564208554	7.27868321347930
H	14.72094238549905	13.13770383754658	6.19389042585148
C	16.04355615971977	14.02433654081347	7.63706122266494
H	13.89000346369326	13.77756955785904	7.61631397578836
C	16.04939204368072	14.52026988072425	9.08835785469164
O	16.10464595148079	15.12974498157976	6.84967117797658
H	16.90641304020652	13.32785605680362	7.53368014051658
Cl	16.18767393938198	13.21685793998494	10.40282419561524
H	15.13966020780902	15.06661232432106	9.32052221204045
H	16.91830665094543	15.15176666293528	9.24427559786111
H	11.82746943021785	12.97443862889275	9.65979456393105

TS2

Freq: -153.36 cm^{-1}

Coordinates from ORCA-job ts2.scants

O	14.67286890449824	14.08227507306121	9.38160472222304
C	14.51854334753301	15.06846118398873	10.22625142472062
C	14.94797365010556	16.38103341680411	9.90189809220509
C	14.87834463288479	17.39776235956016	10.81833566018545
H	15.21569515586195	18.39404920841671	10.55866207427720
C	14.38225916963238	17.17033880846331	12.11660200675388

C	14.28792721857890	18.17095998726715	13.12249210056116
H	14.62711280046647	19.17349158304778	12.88788001432040
C	13.78561592343176	17.89040608455699	14.35027302264268
H	13.70529495740263	18.63481102130845	15.12865857666370
C	13.32331172512493	16.56794744605826	14.67002439058457
O	12.85095281370855	16.19263549141069	15.72595824790080
O	13.42012555173757	15.61752025685770	13.66616862713072
C	13.93886758848775	15.87812386876421	12.42920617638108
H	15.34857655800979	16.55256926687542	8.91178181410578
C	13.96165068333392	14.82246443106155	11.51345728619430
C	13.42900857081272	13.51597014731411	11.90790453512801
H	13.52772275870179	13.24978628854416	12.96485463181448
N	12.87523528903649	12.72398972442209	11.07914813519489
C	12.36398031479004	11.49197525434167	11.48855243638095
C	11.53345223247299	11.33991145275254	12.60159416240624
H	11.27988204317900	12.21414084015446	13.18811422179958
C	11.01542453591379	10.09369595535744	12.93623788285026
H	10.35869609072886	9.99639105060549	13.79148847384882
C	11.33243367634015	8.98008948172800	12.16472956999045
H	10.92488554264265	8.00869683365397	12.41468333106307
C	12.16633662063897	9.11481958612918	11.05986450011466
H	12.40411402474310	8.25540397379081	10.44561131992693
C	12.68266973673934	10.36247826536802	10.70278920216850
O	14.53781673347361	11.62648756883555	7.19210364846388
C	15.45126111806838	10.64696323952520	7.20320485397506
C	16.39447073953756	10.48382449688910	6.17485359932944
H	16.39941880545731	11.15271957189848	5.33139238940172
C	17.34684184666230	9.49506055167991	6.23956485076193
H	18.07286267050770	9.39201443022424	5.44281666036535
C	17.40514292705786	8.62058111964659	7.33055038422951

C	18.37210653620170	7.57800114683764	7.46077454661975
H	19.10677450655016	7.45573417716160	6.67372487168493
C	18.37708050856635	6.75964436679188	8.53541551379686
H	19.09689626253661	5.96397570978642	8.65897932121751
C	17.40000930057399	6.91740976715856	9.58149033739287
O	17.29664579787149	6.25123854306029	10.58954813426979
O	16.47831698211522	7.93986174748239	9.41905708346888
C	16.45760735594069	8.78127108654743	8.34729036426038
C	15.45669059752609	9.77266973043988	8.32281798199302
C	14.52198598226820	9.79645722266279	9.44773132051181
H	14.77112236860471	9.08995043863336	10.23992577758873
N	13.49305673172731	10.54137006259033	9.57133916096587
C	14.24212989861825	12.39044737088855	6.00206714769219
H	14.57919421673582	11.86501891788049	5.11055652715597
C	14.77141766272247	13.82673026365840	6.03672795817806
H	13.15475340706496	12.44308854497874	5.97669358394914
C	16.29045776807223	13.95924391330273	6.13217254551568
O	14.21383983282881	14.58489198519672	7.06615311523335
H	14.45970850842521	14.25422908163032	5.07435439479093
Cl	17.11744265978806	13.53447989103393	4.56479626094244
H	16.72656420774750	13.32067919397429	6.89639220337280
H	16.54804604284012	14.99521916024473	6.32640764439936
H	14.52180994653798	14.32888035282508	8.29761879488210
C	12.38963852316067	15.29735117556700	6.77108568944089
O	12.09945478756448	15.65681069603768	7.85705317065081
O	12.20557264909070	15.13584613325081	5.61503352395060

Int2

Coordinates from ORCA-job int2.2.opt

O	11.59229245945189	13.73244437258825	9.12210277318965
C	12.13575559390459	14.77877743749784	9.74460291792211
C	12.11301282986682	16.02578884427156	9.10004399756877
C	12.69855670581802	17.11706807436602	9.69457297065376
H	12.69469893418688	18.07703977343521	9.19398258969088
C	13.32480047112270	17.01418479809941	10.95061958901290
C	13.96770803446854	18.10450420664098	11.61139263286066
H	13.97263292645357	19.07305672036152	11.12560716604369
C	14.56215479687700	17.93888968446344	12.81407414968646
H	15.05704775927075	18.74661043991668	13.33289931118852
C	14.56385689431599	16.65592383703861	13.46887088818985
O	15.05891780799039	16.38226679411930	14.54086090139743
O	13.92867555563446	15.61848516288083	12.79765961790153
C	13.33019901761991	15.77018870606010	11.58773467836202
H	11.64302367172005	16.09714573995914	8.12884005738501
C	12.74479606352517	14.63195229993339	11.01405739427499
C	12.79557781278908	13.34636932812187	11.68085675018470
H	13.25762020841795	13.30182485312215	12.66584041172917
N	12.33463235507365	12.29527769535881	11.11127423881267
C	12.33675671110747	11.05184542487382	11.75819108646893
C	11.76129288781523	10.87828286338086	13.01676485235507
H	11.31868851884497	11.73272659527876	13.51301072832888
C	11.72430879653188	9.62057174540886	13.60744685018818
H	11.25730407892778	9.49547392109385	14.57583676463558
C	12.26947965748968	8.52624012184027	12.94331467323811
H	12.22760597875834	7.54120465529610	13.39030696768539
C	12.86359313270475	8.69281873886060	11.69813218298182
H	13.28057979469349	7.84210992804756	11.17450098203607

C	12.91136915988414	9.95117270943343	11.09233718959963
O	14.45519386049886	11.89664502259608	7.83675526516996
C	15.27645806531307	10.90373321906239	7.46367959390023
C	16.06067182163524	10.96053128142917	6.29885975653670
H	16.01231243290188	11.82164211400818	5.65178116442475
C	16.90256732320357	9.92231420792467	5.97187723930539
H	17.49867273870369	9.97405964288366	5.06959130455258
C	17.00928965432296	8.79239212331452	6.79256893763961
C	17.86498124597698	7.68239889476952	6.51766047223024
H	18.47128915893268	7.70781113585424	5.61990160472528
C	17.92277954639272	6.62315020199955	7.35458831154154
H	18.56179045109955	5.77176572013202	7.17185242887978
C	17.11851672472715	6.58143268012629	8.54988030723345
O	17.08673814964035	5.69406619116548	9.37592087032778
O	16.28914008211568	7.66865733629588	8.77743115040062
C	16.22696109914518	8.75119806799676	7.95170425201972
C	15.34562418719941	9.77795698442743	8.31697620004278
C	14.62786677095052	9.64893633837146	9.58675699257167
H	15.14415637469307	9.05215125185279	10.34234334345406
N	13.49448588233118	10.17422704668815	9.83725747909337
C	14.52188084666185	13.17632298262282	7.19449983692442
H	14.46492776826240	13.06953851931029	6.11119108145859
C	15.77803432518843	13.97014149644704	7.55258857044462
H	13.62769624644790	13.69324349178238	7.53457516912464
C	15.81301192932039	14.53027515376599	8.96870347242398
O	15.76584194649486	15.08485983379234	6.66864893186078
H	16.67208262772643	13.37182338576679	7.38395380394419
Cl	16.07145840349767	13.25658568672306	10.23404939248208
H	14.88577817299456	15.03645998979504	9.21985169514802
H	16.64952232481421	15.21516225254340	9.06376080966219

H	11.75489526152738	12.92209143163770	9.70541022375528
C	17.02658838202399	15.61867837691938	6.24974439719516
O	16.88961510855079	16.60092719515904	5.50665612654579
O	18.04703669693777	15.04892680168486	6.66632376830664

TS3

Freq: -497.81 cm-1

Coordinates from ORCA-job ts3.2.scants

O	12.05308420463002	13.78277324226842	9.20007308695917
C	12.42437565607625	14.83943363649263	9.92121191631480
C	12.43952342183540	16.09942293288801	9.30248759061195
C	12.83905960505790	17.20688245535692	10.00994635180425
H	12.85904306599440	18.17891090796557	9.53330624604381
C	13.23428873582306	17.10704252192109	11.35703472710949
C	13.66489276027039	18.21691233456257	12.14403133759614
H	13.68889098462826	19.19813156950798	11.68477740187731
C	14.03763976468097	18.05640320179666	13.43407315708806
H	14.36774207571795	18.88014691057173	14.04969893613165
C	14.01116393324040	16.75759305672471	14.05530349784332
O	14.31808821162413	16.48609033896382	15.19667167566478
O	13.58789919721099	15.70038648934781	13.26048299885734
C	13.20996685129740	15.84710548316376	11.96276963513403
H	12.14538569480034	16.16959738801059	8.26447226620480
C	12.81183404519924	14.69176253885092	11.27509707090945
C	12.83603020745319	13.39122588204316	11.91077949147213
H	13.15459029162349	13.34054652501885	12.95095468043732
N	12.52172779605159	12.33453097743243	11.25296490410563
C	12.48506518543242	11.07282913100347	11.85896571214982
C	11.93795123079270	10.88233190739571	13.13012983522684
H	11.53688261147700	11.73562290351759	13.66218099991735

C	11.86906955390841	9.61383747173089	13.69068562589991
H	11.42426793449368	9.48030689971135	14.66836594022417
C	12.34817947180861	8.51787543910818	12.97856201099100
H	12.27962727954656	7.52257168830495	13.39925654132559
C	12.90007038413057	8.69498687658751	11.71663388834299
H	13.25186850952725	7.83974313288441	11.15426338000293
C	12.98549715091970	9.96654633821107	11.13671767852054
O	14.18053510332316	11.62551598445230	7.50694258274611
C	15.00687154858369	10.67725437723545	7.25972363964286
C	15.75258031663075	10.63433494580261	6.03771424690757
H	15.57402308767344	11.40601299784742	5.30224297867336
C	16.65859577388991	9.64497140699096	5.77894900113705
H	17.20307774202385	9.63370576142353	4.84206877756760
C	16.90274608648959	8.61685000398300	6.71241059870403
C	17.82703781382636	7.56035272830034	6.50433945135858
H	18.38663840954466	7.54088884133965	5.57604275614422
C	18.01766044338373	6.59152356713806	7.43606584248856
H	18.71649866835346	5.78005188841576	7.29666226146080
C	17.27660218390401	6.60728141240440	8.66355846305915
O	17.34601899448464	5.80589134795119	9.57690639531633
O	16.37381390569256	7.64764356605765	8.82909867112414
C	16.17729591529973	8.64003361980944	7.91099343904043
C	15.23170729884709	9.62886117845680	8.22390584025763
C	14.58053529374297	9.56340719418198	9.52081107418759
H	15.08403410571451	8.92024784635634	10.24614484197669
N	13.51164469614860	10.18331345169577	9.85998404402561
C	14.26841541399104	13.44528027332403	6.69705430206629
H	14.04228923736435	13.02732289173257	5.73457146641447
C	15.69768560389901	13.79589310596122	7.03112039160450
H	13.46651940428703	13.64303673135711	7.38183378065024

C	15.88200401468757	14.55309395473671	8.34256259396639
O	16.21244535490328	14.63387698184336	5.98339958673274
H	16.32428481335196	12.90760995279477	7.02717805884382
Cl	15.77769338106312	13.47004785467177	9.78233431077445
H	15.12951625019405	15.32613556387380	8.47300680875365
H	16.87506558754909	14.99134229356312	8.36104692152643
C	15.24389280087790	15.49497091341534	5.48361535247567
O	15.60981394590521	16.36575550363057	4.70671838337690
O	14.07096779197363	15.23967833221445	5.91149068334032
H	12.16077319713769	12.96553334569054	9.79175386888503

2a

Coordinates from ORCA-job cc.opt

C	-2.18042375023779	-0.88781076690185	2.60281893968408
C	-1.70726596617894	0.49459258909873	2.13015247604258
O	-3.30001421940678	-0.57151768046138	3.45989034348978
C	-3.63414748855806	0.72509144668630	3.33506707300168
O	-4.58206680883145	1.22972456065781	3.86815314048947
O	-2.76897937664294	1.37898524406839	2.54817429372009
H	-1.60782268023882	0.56590909862975	1.05020317976459
H	-0.78734008506381	0.80813126631960	2.61818856263527
H	-1.42873041613583	-1.38568760918822	3.21546198849489
C	-2.60550147396240	-1.85199998677741	1.52095238461854
Cl	-3.86676415614474	-1.14424617288055	0.43398616359613
H	-3.03513958573064	-2.75139261717563	1.95314702795700
H	-1.75259323586773	-2.10949491957555	0.89711588360585

5. References

(1) Nuñez-Dallos, N.; Cuadrado, C.; Hurtado, J.; Nagles, E.; García-Beltran, O. In Situ-Mercury Film Electrode for Simultaneous Determination of Lead and Cadmium Using Nafion Coated New Coumarin Schiff Base as Chelating-Adsorbent. *Int. J. Electrochem. Sci.* **2016**, *11*, 9855–9867. https://doi.org/10.20964/2016.12.02.

(2) Nuñez-Dallos, N.; Posada, A. F.; Hurtado, J. Coumarin Salen-Based Zinc Complex for Solvent-Free Ring Opening Polymerization of ε-Caprolactone. *Tetrahedron Letters* **2017**, *58* (10), 977–980. https://doi.org/10.1016/j.tetlet.2017.01.088.

(3) Kilic, A.; Koyuncu, I.; Durgun, M.; Ozaslan, I.; Kaya, İ. H.; Gönel, A. Synthesis and Characterization of the Hemi-Salen Ligands and Their Triboron Complexes: Spectroscopy and Examination of Anticancer Properties. *Chemistry & Biodiversity* **2018**, *15* (1), e1700428. https://doi.org/10.1002/cbdv.201700428.

(4) Sharma, V.; Arora, E. K.; Cardoza, S. Synthesis, Antioxidant, Antibacterial, and DFT Study on a Coumarin Based Salen-Type Schiff Base and Its Copper Complex. *Chemical Papers* **2016**, *70* (11), 1493–1502. https://doi.org/10.1515/chempap-2016-0083.

(5) Jones, R. N.; Angell, C. L.; Ito, T.; Smith, R. J. D. The Carbonyl Stretching Bands in the Infrared Spectra of Unsaturated Lactones. *100 Years of CSC in the Pages of CJC* **2017**, *01* (01), 2007–2022. https://doi.org/10.1139/v59-293@cjc-csc.issue01.

(6) Winston, A.; Kemper, R. N. The Split Carbonyl Band in the Infrared Spectra of Halogen Derivatives of 4-Hydroxy-2,4-Pentadienoic Acid Lactone. *Tetrahedron* **1971**, *27* (3), 543–548. https://doi.org/10.1016/S0040-4020(01)90723-9.

(7) Larkin, P. *Infrared and Raman Spectroscopy: Principles and Spectral Interpretation*; Elsevier, 2017.

(8) Kajal, A.; Bala, S.; Kamboj, S.; Sharma, N.; Saini, V. Schiff Bases: A Versatile Pharmacophore. *Journal of Catalysts* **2013**, *2013*, e893512. https://doi.org/10.1155/2013/893512.

(9) Aranha, P. E.; dos Santos, M. P.; Romera, S.; Dockal, E. R. Synthesis, Characterization, and Spectroscopic Studies of Tetradentate Schiff Base Chromium(III) Complexes. *Polyhedron* **2007**, *26* (7), 1373–1382. https://doi.org/10.1016/j.poly.2006.11.005.
